# Association between Metabolic Obesity Phenotypes and the Burden of Hospitalized Postmenopausal Patients Concomitant with Osteoporosis: A Retrospective Cohort Study Based on the National Readmission Database

**DOI:** 10.3390/jcm12041623

**Published:** 2023-02-17

**Authors:** Jie Jiang, Chao Xu, Zinuo Yuan, Junming Han, Zhixiang Wang, Yang Tian, Yingchun Dong, Weibo Xia, Xiude Fan, Jiajun Zhao

**Affiliations:** 1 Department of Endocrinology, Shandong Provincial Hospital, Shandong University, Jinan 250021, China; 2 Department of Endocrinology, Shandong Provincial Hospital Affiliated to Shandong First Medical University, Jinan 250021, China; 3 Shandong Clinical Research Center of Diabetes and Metabolic Diseases, Jinan 250021, China; 4 Shandong Key Laboratory of Endocrinology and Lipid Metabolism, Jinan 250021, China; 5 Shandong Prevention and Control Engineering Laboratory of Endocrine and Metabolic Diseases, Jinan 250021, China; 6 Department of Endocrinology, Key Laboratory of Endocrinology, National Commission of Health, State Key Laboratory of Complex Severe and Rare Diseases, Peking Union Medical College Hospital, Chinese Academy of Medical Sciences, Beijing 100730, China

**Keywords:** metabolic obesity phenotype, postmenopausal osteoporosis, hospitalized burden, 30-day readmission, 90-day readmission

## Abstract

Background: The present definition of obesity based on body mass index (BMI) is not accurate and effective enough to identify hospitalized patients with a heavier burden, especially for postmenopausal hospitalized patients concomitant with osteoporosis. The link between common concomitant disorders of major chronic diseases such as osteoporosis, obesity, and metabolic syndrome (MS) remains unclear. Here, we aim to evaluate the impact of different metabolic obesity phenotypes on the burden of postmenopausal hospitalized patients concomitant with osteoporosis in view of unplanned readmissions. Methods: Data was acquired from the National Readmission Database 2018. The study population was classified into metabolically healthy non-obese (MHNO), metabolically unhealthy non-obese (MUNO), metabolically healthy obese (MHO), and metabolically unhealthy obese (MUO) patients. We estimated the associations between metabolic obesity phenotypes and 30- and 90-day unplanned readmissions. A multivariate Cox Proportional Hazards (PH) model was used to assess the effect of factors on endpoints, with results expressed as HR and 95% CI. Results: The 30-day and 90-day readmission rates for the MUNO and MUO phenotypes were higher than that of the MHNO group (all *p* < 0.05), whereas no significant difference was found between the MHNO and MHO groups. For 30-day readmissions, MUNO raised the risk mildly (hazard ratio [HR] = 1.110, *p* < 0.001), MHO had a higher risk (HR = 1.145, *p* = 0.002), and MUO further elevated this risk (HR = 1.238, *p* < 0.001). As for 90-day readmissions, both MUNO and MHO raised the risk slightly (HR = 1.134, *p* < 0.001; HR = 1.093, *p* = 0.014, respectively), and MUO had the highest risk (HR = 1.263, *p* < 0.001). Conclusions: Metabolic abnormalities were associated with elevated rates and risks of 30- or 90-day readmission among postmenopausal hospitalized women complicated with osteoporosis, whereas obesity did not seem to be innocent, and the combination of these factors led to an additional burden on healthcare systems and individuals. These findings indicate that clinicians and researchers should focus not only on weight management but also metabolism intervention among patients with postmenopausal osteoporosis.

## 1. Introduction

Hospital admissions cause a significant burden on healthcare systems worldwide, and unplanned readmissions have become major safety and medical economic issues as indicators of healthcare quality, leading to additional healthcare expenditure and an increased burden on medical systems, society, and individuals [1]. The World Health Organization (WHO) has described osteoporosis as a bone mineral density (BMD) less than 2.5 standard deviations (SD) (T score ≤ −2.5) compared to pre-menopausal women [2]. It was estimated that 10.2 million adults were diagnosed with osteoporosis and 43.4 million adults had low bone mass in the US in 2010 [3]. Osteoporosis is a common concomitant disorder of major chronic diseases such as cardiovascular disease, chronic obstructive pulmonary disease (COPD), and Type 2 diabetes mellitus (T2DM) [4]. Patients suffering from at least one major chronic disease accompanied with osteoporosis face 13–23% higher healthcare costs than those without osteoporosis [4]. The prevalence of osteoporosis among postmenopausal women is approximately 30%, which increases gradually as their age grows [2,5]. Nevertheless, osteoporosis tends to be neglected as it usually keeps silent before it leads to one or more fractures [6]. In fact, more than half of postmenopausal women will develop fractures during their lifetimes, resulting in increased chances of disability and death, with subsequent raised expenditure [7]. Statistics have shown that the annual cost for osteoporotic fracture caring exceeds that for myocardial infarction, breast cancer, or stroke in women ≥55 years old [7,8]. As life expectancy around the world’s population increases, the burden of hospitalized postmenopausal patients complicated with osteoporosis also rises.

Obesity has become a substantial public health threat among developed and developing countries, accompanied by a growing incidence, serious negative impact on individual health, and a considerable socioeconomic burden [9]. The link between obesity and osteoporosis has been controversial for a long time. Historically, obesity was identified as a protective factor for bone health, as heavier individuals have a higher BMD and lower risks for osteoporosis [10]. However, recent studies have indicated that obesity was associated with a reduced BMD and elevated risk of fracture in some cases [11]. Adipose tissue deposited in the abdomen, viscera, and bone marrow are negatively associated with poor bone health [11,12]. It is proposed that the metabolic state plays a role in these conflicted observations [11].

Menopause is considered as a high-risk factor for weight gain as well as osteoporosis [9,13,14]. Clinical studies have shown that postmenopausal women are more susceptible to developing MS, including obesity, dyslipidemia, hypertension, or impaired glucose tolerance, compared with pre-menopausal women [15,16,17]. Another report proved that dietary intervention and exercise guidance were associated with considerable weight loss and MS alleviation in postmenopausal women [17]. In addition, obese women were at a somewhat higher risk of MS when they entered menopause [18,19]. These findings together showed the complicated associations between obesity, metabolic health, and menopause. However, the present definition of obesity based on BMI lumps individuals with different clinical characteristics into the same category, which is not accurate and effective enough to identify hospitalized patients with a heavier burden, especially for postmenopausal hospitalized patients concomitant with osteoporosis. Here, we sought to estimate the impact of different metabolic obesity phenotypes on the burden of hospitalized postmenopausal patients concomitant with osteoporosis in the view of readmission, length of stay (LOS), and total charges (TOTCHG).

## 2. Materials and Methods

### 2.1. Data Source

We performed this retrospective cohort study using the National Readmission Database (NRD) 2018 [20]. The NRD is a collection of hospitalized patient databases of the Health Cost and Utilization Project (HCUP) family, which is designed for analyses of readmission. The Agency of Healthcare Research and Quality (AHRQ) supports HCUP via a Federal–State–Industry partnership. Data from the NRD are generated annually and contains approximately 57.8% of all-age, all-payer discharges across 28 geographically separated states in the US [21]. The NRD provides International Classification of Diseases, Tenth Revision, Clinical Modification (ICD-10-CM). It also provides patient-related demographic data and hospital-related information, as well as discharge information. Institutional Review Board (IRB) approval is not required as the NRD is a national database. Information provided by the NRD is deidentified to protect the privacy of the patient, physician, and hospital. Additionally, these NRD datasets were accessed in compliance with the Health Insurance Portability and Accountability Act of 1996. Henceforth, this study was exempted from Institutional Review Board (IRB) approval. This study was based on the Strengthening the Reporting of Observation Studies in Epidemiology (STROBE) recommendations and complied with the United States Agency for Healthcare Research and Quality’s Healthcare Cost and Utilization Project Data Use Agreement and was exempt from research ethics board review.

### 2.2. Data Collection

We estimated the burden of hospitalized postmenopausal women concomitant with osteoporosis in view of the rates of unplanned 30- and 90-day readmissions, and associations between metabolic obesity phenotypes and readmissions. We included hospitalized female patients above 55 years old diagnosed with primary osteoporosis according to the ICD-10-CM codes, containing M80 and M81 (Table S1). We excluded patients who were underweight (BMI < 20) or died during their initial hospitalization. Individuals with missing data of baseline characteristics were also excluded. Considering that patients from one calendar year cannot be tracked to the previous or next year, patients discharged during December (Dec) were excluded from the 30-day readmissions study. Similarly, patients discharged during October (Oct) to Dec were not taken into study for 90-day readmissions.

### 2.3. Definitions

Patients were classified into non-obese (BMI < 25 kg/m^2^) status and obese (BMI ≥ 25 kg/m^2^, including overweight and obesity) status based on prior studies [22]. MS was defined using the harmonized American Heart Association/National Heart, Lung, and Blood Institute (AHA/NHLBI) and International Diabetes Federation (IDF) criteria [23,24]. Waist circumference was excluded from the MS definition, as it is strongly collinear with BMI. Metabolically unhealthy status was defined as having two or more components of MS: (1) hyperglycemia, fast plasma glucose (FPG) ≥ 6.1 mmol/L; (2) hypertension, systolic blood pressure (SBP) ≥ 130 mmHg, or diastolic blood pressure (DBP) ≥ 85 mmHg; (3) dyslipidemia, triglycerides (TGs) ≥ 1.7 mmol/L, or high-density lipoprotein cholesterol (HDL-C) < 1.0 mmol/L.

According to BMI categories and metabolic statuses, patients were classified into four metabolic obesity types: (1) metabolically healthy non-obese (MHNO); (2) metabolically unhealthy non-obese (MUNO); (3) metabolically healthy obese (MHO); and (4) metabolically unhealthy obese (MUO). We also divided the study population into eight groups based on specific metabolic disorder and BMI: (1) non-obese with no metabolic disorder; (2) non-obese with dyslipidemia; (3) non-obese with hypertension; (4) non-obese with hyperglycemia; (5) obese with no metabolic disorder; (6) obese with dyslipidemia; (7) obese with hypertension; (8) obese with hyperglycemia. In addition, patients were also classified into another eight groups on the basis of BMI and their number of MS risk factors: (1) non-obese, no MS risk factor; (2) non-obese, 1 MS risk factor; (3) non-obese, 2 MS risk factors; (4) non-obese, 3 MS risk factors; (5) obese, no MS risk factor; (6) obese, 1 MS risk factor; (7) obese, 2 MS risk factors; (8) obese, 3 MS risk factors.

### 2.4. Outcome

The outcome of our study was non-elective 30- and 90-day readmissions of hospitalized postmenopausal patients complicated with osteoporosis among different metabolic obesity phenotypes. Readmission was defined as any unplanned all-cause readmissions concomitant with osteoporosis over the given period of our study. We also investigated the prevalence of fragility fractures at baseline, and the LOS and TOTCHG of non-elective readmissions at 30 and 90 days among the four metabolic obesity phenotypes. The ICD-10-CM codes for fragility fractures are summarized in Table S1.

### 2.5. Statistical Analysis

The data normality test was conducted using a Kolmogorov–Smirnov test. Continuous variables were presented as medians and inter-quartile ranges (IQR) as appropriate, while categorical variables were presented as numbers (percentage). Continuous variables were then compared by Kruskal–Wallis test, while categorical variables were analyzed using the chi-square (χ^2^) test. Parameters with *p* values < 0.05 at univariate analysis were entered into the multivariate Cox Proportional Hazards (PH) model to assess the effect of factors on endpoints, with results expressed as HR and 95% CI. In addition, a sensitivity analysis was performed in postmenopausal woman who were more than 75 years old of age. A two-tailed *p* value < 0.05 was identified as statistically significant. All statistical analysis was run with SPSS version 25 (SPSS, Chicago, IL, USA).

## 3. Results

### 3.1. Baseline Characteristics of Participants According to Metabolic Obesity Phenotypes

**Figure 1:**
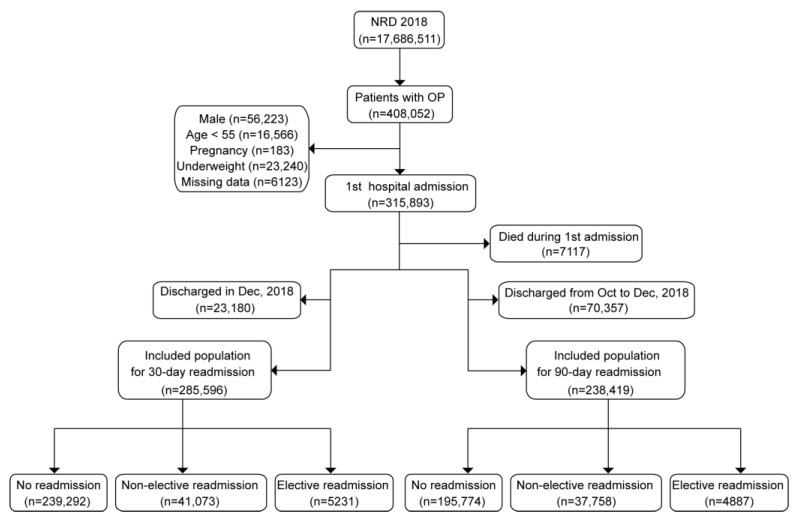
Inclusion flow chart of study population.

Our research identified 408,052 potentially eligible patients; after ruling out patients who met the exclusion criteria, 285,596 were left for 30-day readmission analysis and 238,419 were left for 90-day readmission analysis. A detailed description for inclusion and exclusion is shown in the flow chart (Figure 1). The baseline characteristics and demographics of 30-day readmission participants are shown in Table S2. For 30-day readmissions, MHO and MUO patients were younger at first admission, with a longer LOS and greater TOTCHG during hospitalization, compared with MHNO and MUNO participants (*p* < 0.05). At baseline, elective admission was more common among obese (33.7% for MHO and 26.6% for MUO) patients (*p* < 0.05), compared to non-obese (20.9% for MHNO and 16.7% for MUNO) patients. A higher proportion of MUNO (83.3%) had non-elective admissions than that of MHNO (79.1%) in the non-obese population, with a similar result seen among obese patients (73.4% for MUO, and 66.3% for MHO) (*p* < 0.05). As for the median household income, the proportion of obese patients was higher in the inferior quartile (23.5% for MHO and 24.7% for MUO) and lower in the superior quartile (21.8% for MHO and 20.5% for MUO) (*p* < 0.05). MUNO and MUO patients were older and included fewer smokers or drinkers than MHNO and MHO patients, respectively (*p* < 0.05), with no difference found in physical activity among the four groups. MHO and MUO participants had lower percentages of not meeting any HCUP emergency department record (HCUP ED) criteria than MHNO and MUNO patients, while MUNO and MUO patients had significantly lower proportions of the above criteria than MHNO and MHO patients (*p* < 0.05). MHO and MUO participants had lower percentages of HCUP emergency department records (HCUP ED) than MHNO and MUNO patients, while MUNO and MUO patients had significantly lower proportions of the HCUP ED records than MHNO and MHO patients (*p* < 0.05). As for the primary expected payer, 90.9% of MUNO patients had Medicare, which was higher than that of MHNO patients (85.5%), while a higher percentage of MUO patients (85.8%) had Medicare than MHO patients (80.3%) (*p* < 0.05). In “central” counties of metro areas of ≥1 million population, the proportions of MUNO and MUO participants were higher than that of MHNO and MHO patients (*p* < 0.05). More obese (2.6% for MHO and 3.0% for MUO, respectively) patients had a combined record involving rehab transfer, compared to non-obese (1.9% for MNHO and 2.2% for MUNO) participants (*p* < 0.05). The proportion of MUNO and MUO participants whose state was the same as the hospital state (resident) was higher than that of MHO and MUO patients (*p* < 0.05). The baseline characteristics of 90-day readmissions are summarized in Table S3, with the results similar to that of 30-day readmissions.

### 3.2. Metabolically Unhealthy Statuses Rather Than Obesity Elevated Unplanned Readmission Rates

**Figure 2:**
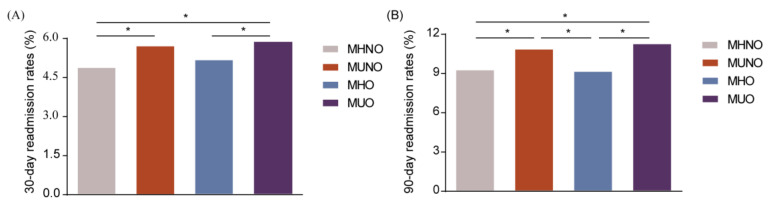
The 30- and 90-day readmission rates of metabolic obesity phenotypes among hospitalized postmenopausal women concomitant with osteoporosis. (**A**) The 30-day readmission rates among metabolic obesity phenotypes for the study population. (**B**) The 90-day readmission rates among metabolic obesity phenotypes for the study population. * *p* < 0.05. MHNO: metabolically healthy non-obese, MUNO: metabolically unhealthy non-obese, MHO: metabolically healthy obese, MUO: metabolically unhealthy obese.

Compared to the MHNO group, the MUNO group had a significantly higher rate of 30-day readmission (5.7% vs. 4.9%, *p* < 0.05), the MUO group had a higher 30-day readmission rate than that of the MHO group (5.9% vs. 5.2%, *p* < 0.05), and no statistical differences were found between the MUNO and MHO participants (Figure 2A). A higher proportion of MUNO patients was readmitted to hospital at 90 days, compared to that of MHNO patients (10.9% vs. 9.3%, *p* < 0.05), even higher than that of the MHO group (10.9% vs. 9.2%, *p* < 0.05) (Figure 2B). The MUO group had a higher 90-day readmission rate than the MHO group (11.3% vs. 9.2%, *p* < 0.05) (Figure 2B). There were no differences in 30- or 90-day readmission incidence among the subgroups of metabolically healthy (MHO vs. MHNO) or metabolically unhealthy (MUO vs. MUNO) (Figure 2A,B).

### 3.3. Associations of Metabolically Obesity Phenotypes with 30- or 90-Day Readmissions

**Figure 3:**
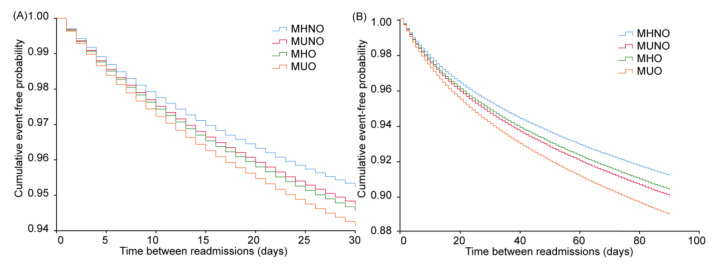
Cumulative event-free probability of different metabolic obesity phenotypes among hospitalized postmenopausal women concomitant with osteoporosis. (**A**) The 30-day and (**B**) 90-day readmission of different metabolic obesity phenotypes among hospitalized postmenopausal women concomitant with osteoporosis. MHNO: metabolically healthy non-obese, MUNO: metabolically unhealthy non-obese, MHO: metabolically healthy obese, MUO: metabolically unhealthy obese.

Overall, MUNO or MHO patients had mildly higher risks of either 30-day or 90-day readmissions, compared with MHNO participants, with risks were significantly higher for MUO participants. For 30-day readmissions, the MUNO phenotype raised the risk mildly (HR = 1.110, 95% CI = 1.073–1.149, *p* < 0.001), the MHO phenotype had a higher risk (HR = 1.145, 95% CI = 1.050–1.250, *p* = 0.002), and MUO further elevated this risk (HR = 1.238, 95% CI = 1.166–1.316, *p* < 0.001) (Table S4 and Figure 3). As for 90-day readmissions, MUNO or MHO status raised the risk slightly (HR = 1.134, 95% CI = 1.104–1.166, *p* < 0.001; HR = 1.093, 95% CI = 1.018–1.174, *p* = 0.014, respectively), while the MUO phenotype had the highest risk (HR = 1.263, 95% CI = 1.204–1.325, *p* < 0.001) (Table S4 and Figure 3).

### 3.4. Associations between Obesity Categories with/without Specific Metabolic Disorder and 30-Day or 90-Day Readmissions

**Figure 4:**
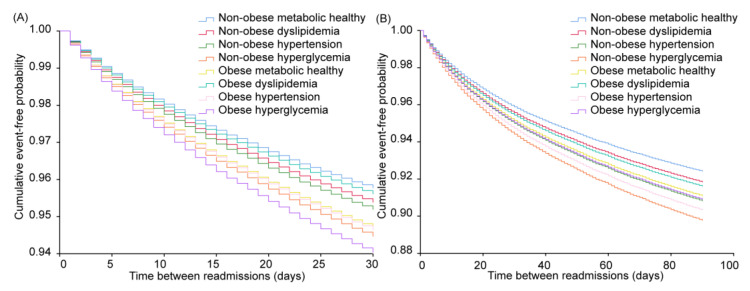
Cumulative event-free probability of obesity categories with/without specific metabolic disorder among hospitalized postmenopausal osteoporotic women. (**A**) The 30-day and (**B**) 90-day readmission of different obesity categories with/without specific metabolic disorder subgroups among hospitalized postmenopausal women concomitant with osteoporosis, including non-obese metabolic healthy, non-obese dyslipidemia, non-obese hypertension, non-obese hyperglycemia, obese metabolic healthy, obese dyslipidemia, obese hypertension, and obese hyperglycemia.

In the non-obese population, any single metabolic disorder was related to the elevated risk of 30- or 90-day readmissions. Results were sorted in descending order as follows: single hyperglycemia (HR = 1.317, 95% CI = 1.141–1.520, *p* < 0.001), hypertension (HR = 1.140, 95% CI = 1.072–1.212, *p* < 0.001), and dyslipidemia (HR = 1.093, 95% CI = 1.006–1.187, *p* = 0.035) raised the risks of 30-day readmission, compared to the phenotype without metabolic disorders (Table S5 and Figure 4). The results of 90-day readmissions were similar to those of 30-day readmissions. The hyperglycemia subgroup had the highest risk (HR = 1.368, 95% CI = 1.220–1.535, *p* < 0.001), followed by hypertension (HR = 1.220, 95% CI = 1.162–1.282, *p* < 0.001) and dyslipidemia (HR = 1.079, 95% CI = 1.009–1.155, *p* = 0.027). In terms of obese patients, compared to the group without metabolic disorders, hyperglycemia showed the highest risk (HR = 1.423, 95% CI = 1.035–1.955, *p* = 0.030) in 30-day readmissions, but did not change the risk in 90-day readmissions (*p* = 0.180) (Table S5 and Figure 4). The risk of hypertension was increased (HR = 1.274, 95% CI = 1.131–1.435, *p* < 0.001) at 30-day readmissions, as well as at 90-day readmissions (HR = 1.290, 95% CI = 1.172–1.420, *p* < 0.001) (Table S5 and Figure 4).

### 3.5. Associations between Obesity Categories Combined with Number of MS Risks and 30-Day or 90-Day Readmissions

**Figure 5:**
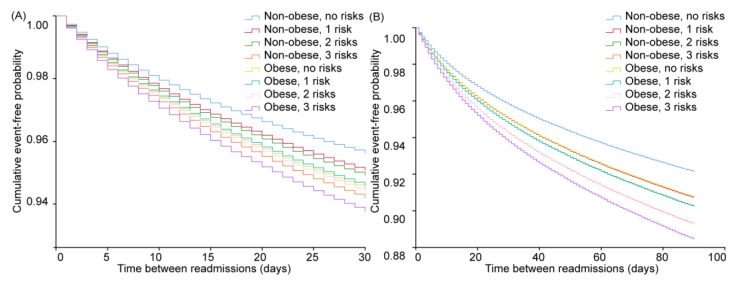
Cumulative event-free probability of number of MS disorders among hospitalized postmenopausal women concomitant with osteoporosis. (**A**) The 30-day and (**B**) 90-day readmission of subgroups of number of MS disorders among hospitalized postmenopausal women concomitant with osteoporosis, including non-obese with no risk factor, non-obese with 1 risk factor, non-obese with 2 risk factors, non-obese with 3 risk factors, obese with no risk factor, obese with 1 risk factor, obese with 2 risk factors, and obese with 3 risk factors.

As shown in Table S6 and Figure 5, the risks of 30-day or 90-day readmissions were elevated as the numbers of metabolic disorder increased among obese and non-obese participants. For non-obese participants, the risks of 30-day readmissions increased as the number of MS risk factors grew, expressed by HR (95% CI, *p* value) of 1.132 (1.068–1.199, *p* < 0.001) (1 MS risk factor), 1.169 (1.104–1.238, *p* < 0.001) (2 MS risk factors), and 1.339 (1.255–1.429, *p* < 0.001) (3 MS risk factors), respectively. Simple obesity with no MS disorder had a higher risk (HR = 1.267, 95% CI = 1.057–1.518, *p* = 0.010) for 30-day readmissions, with risks further raised as the number of MS disorders increased, indicated by HR (95% CI, *p* value) of 1.247 (1.121–1.387, *p* < 0.001) (1 MS risk factor), 1.289 (1.179–1.409, *p* < 0.001) (2 MS risk factors), and 1.440 (1.311–1.583, *p* < 0.001) (3 MS risk factors), respectively. The risks of 90-day readmissions for non-obese participants was elevated with the number of MS disorders, expressed by HR (95% CI, *p* value) of 1.192 (1.137–1.249, *p* < 0.001) (1 MS risk factor), 1.256 (1.199–1.315, *p* < 0.001) (2 MS risk factors), and 1.386 (1.315–1.460, *p* < 0.001) (3 MS risk factors), respectively. Simple obesity without the MS disorder subgroup had a higher risk of 90-day readmissions (HR, 1.187; 95% CI, 1.019–1.383, *p* = 0.027), while this risk was lower than the non-obese subgroups mentioned above (Table S6 and Figure 5). As the number of MS disorders increased, the risks of 90-day readmissions were raised, ranging from 1.254 (1.150–1.367, *p* < 0.001) (1 MS risk factor) to 1.500 (1.391–1.617, *p* < 0.001) (3 MS risk factors).

### 3.6. Prevalence of Fragility Fractures at Initial Admission, and LOS and TOTCHG among Metabolically Obesity Phenotypes during Unplanned 30- and 90-Day Readmissions

**Figure 6:**
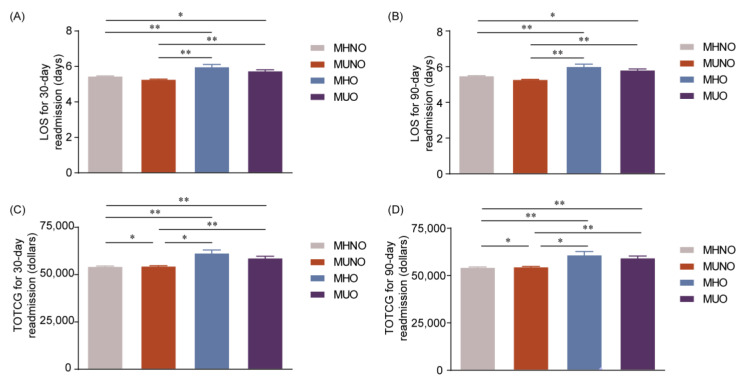
LOS and TOTCHG among metabolically obesity phenotypes during unplanned 30- and 90-day readmissions. (**A**) Length of stay (LOS) at 30-day readmissions, (**B**) LOS at 90-day readmissions, (**C**) total charge (TOTCHG) at 30-day readmissions, and (**D**) TOTCHG at 90-day readmissions. * *p* < 0.05, ** *p* < 0.01. MHNO: metabolically healthy non-obese, MUNO: metabolically unhealthy non-obese, MHO: metabolically healthy obese, MUO: metabolically unhealthy obese.

Non-obese patients had a significantly higher prevalence of fragility fractures (46.0% for MHNO and 45.2% for MUNO) at initial admission, compared to obese patients (3.1% for MHO and 5.7% for MUO). In addition, metabolic unhealthy patients had a lower prevalence of fragility fractures in non-obese patients but a higher prevalence of fragility fractures in obese participants (Figure S1). However, the most common reason for unplanned 30- and 90-day readmission was sepsis (data not shown), which was not influenced by the metabolic healthy status or obesity. Moreover, obese (MHO and MUO) statuses increased the LOS of 30-day and 90-day readmissions, compared with non-obese (MHNO and MUNO) statuses (all *p* < 0.05) (Figure 6A,B), regardless of metabolic statuses. Obesity (MHO and MUO) increased TOTCHG of 30-day and 90-day readmission participants, compared with non-obesity (MHNO and MUNO) (all *p* < 0.05), with the TOTCHG of the MUNO group higher than that of the MHNO group (*p* < 0.05) (Figure 6C,D).

## 4. Discussion

In this retrospective cohort study amassed from NRD 2018, there were four main findings: (1) the 30-day readmission rate of MHNO, MUNO, MHO, and MUO was 4.9%, 5.7%, 5.2%, and 5.9%, respectively. (2) The 90-day readmission rate of MHNO, MUNO, MHO, and MUO was 9.3%, 10.9%, 9.2%, and 11.3%, respectively. (3) Metabolically unhealthy profiles with or without obesity were found to be associated with increased risks of 30- and 90-day readmissions. (4) Metabolically healthy phenotypes were also related to the LOS and TOTCHG during unplanned readmission at 30 and 90 days.

Hospital admissions cause a major burden on healthcare systems worldwide, and unplanned hospital readmissions exacerbate this burden significantly [1,25]. Readmissions refer to emergency/unplanned admissions that occur within a certain interval after initial hospital admissions [1]. An estimated one fifth of all hospitalized patients were readmitted in 2008, with dramatic expenses [25]. Since then, the US Hospital Readmission Reduction Program (HRRP) was implemented by the Patient Protection and Affordable Care Act in 2012 to reduce the payments to the hospital in order to alleviate the financial burden and, meanwhile, readmission was considered an event to avoid by HRRP [26,27]. Readmission can be caused by patient factors (e.g., accidents, natural proceeding of disease, or poor compliance), while it can also result from inadequate care (e.g., insufficient monitoring of adverse events, inappropriate discharge) [1,28,29,30]. Clinical studies for readmission rates for holistic osteoporosis (with or without fractures) are scanty, and the one published clinical research was conducted by Ruff et al. with readmission rates of 5.6% (30 days) and 10.9% (90 days) [1]. In our research, the readmission rates of metabolically healthy phenotypes were lower, while the rates of metabolically unhealthy phenotypes were higher, compared to the results of Ruff’s study, regardless of obesity [1]. Our results indicated that the metabolic condition, to some extent, might play a more important role in readmission rates for postmenopausal osteoporosis than obesity.

Osteoporosis is a type of common bone disorder defined by WHO, characterized by low BMD and the deterioration of bone micro-architecture, leading to raised bone fragility and subsequent increased fracture risks [2]. Clinicians care more about conditions after osteoporotic fractures have occurred; however, it is more important to prevent and detect osteoporosis before fractures happen, in order to avoid unnecessary readmissions, additional healthcare costs, and an increased burden on medical systems, society, and individuals [4,31]. Osteoporosis is common among postmenopausal women, and pain, frailty or fall-associated fractures usually cause readmission, which can be avoided effectively by adequate pharmacotherapy and favorable management [32]. A recently postmenopausal woman has an average bone loss rate of 1%/year at the hip, 2–3%/year at the spine, and a lower rate in elder women [33]. Although obesity was regarded as a protective factor for osteoporosis for a long time [10], there was growing evidence indicating the negative effect of obesity on bone health, with obesity considered to be associated with increased risks of fracture in osteoporotic patients [11]. The distribution of adipose tissue was related to skeletal health, with abdominal adipose tissue, visceral adipose tissue, and bone marrow adipose tissue all negatively related to bone health [11,12,34]. Menopause was found to be associated with increased risk of MS, including obesity, dyslipidemia, hypertension, and impaired glucose tolerance [15,16,17]. A study from Germany demonstrated that dietary intervention and exercise guidance were associated with considerable weight loss and alleviation of MS in postmenopausal women [17]. Another observation pointed out that obesity combined with T2DM had poorer bone status in males, compared to those who were obese but without T2DM [10]. In the present study, we found that metabolic abnormalities were associated with elevated rates and risks of 30- or 90-day readmission among postmenopausal hospitalized women complicated with osteoporosis, which suggested that metabolic abnormities might play an important role in bone metabolism among postmenopausal women.

Recently, growing interest has focused on a subgroup of metabolic obesity phenotypes known as MHO, comprising approximately 10–30% of the obese population, and which is considered as reducing the risk of obesity-related comorbidities [35,36]. However, whether postmenopausal women with MHO are protected from these risks is still unknown. In addition, the combined impact of obesity and metabolic status on the burden of postmenopausal women diagnosed with osteoporosis remains unclear. Our observations indicated that MUNO, MHO, and MUO were risk factors of 30- and 90-day readmissions. Notably, HR of MUNO was lower than that of MHO at 30 days, while HR of MUNO was higher than that of MHO at 90 days, suggesting metabolic status has a greater effect on patients’ prognosis than obesity per se, especially in the long term. Therefore, obesity was not seen to be innocent in the readmission of postmenopausal hospitalized women complicated with osteoporosis, and the combination of metabolic abnormities and obesity led to an additional burden on healthcare systems and individuals.

We then took a closer look at the specified metabolic disorder. Results indicated that any type of metabolic abnormality was associated with elevated risks of both 30- and 90-day readmissions among non-obese patients. Unexpectedly, obesity with no metabolic disorder and obesity with only hypertension were stable risk factors of 30- and 90-day readmissions, hyperglycemia was related to an increased risk of 30-day readmissions, and no significant correlation was shown for dyslipidemia and readmissions. Previous studies have reported contradictory effects of dyslipidemia, hyperglycemia, and hypertension on osteoporosis [37,38,39,40,41,42,43]. Our study further demonstrated the different association between each feature of MS and osteoporosis. Next, we analyzed the association between the number of MS risk factors and readmissions. Whether in the non-obese or obese population, the risk of 30-day and 90-day readmissions increased as the number of MS disorders grew. The greater the number of MS disorders that one had, the greater the risk of hospital readmission, no matter whether one was obese or not. The results of our study showed not only associations between MS and readmissions in postmenopausal women with osteoporosis, but also an increased risk of readmissions with the numbers of MS elements in this population.

Finally, we compared the prevalence of fragility fractures at initial admission, and the LOS and TOTCHG at 30- and 90-day readmissions among different metabolic obesity phenotypes. Our present study showed that obese patients had significantly lower prevalence of fragility fractures, which may be caused by the elevated mechanical load on bone in obese individuals that stimulates the release of obesity-related hormones, such as leptin, estrogen, and insulin, and contributed to the bone growth and remodeling in this population [10]. However, in our study, the most common reason for the 30- and 90-day readmissions in participants with four different metabolic obesity phenotypes was sepsis and not fragility fractures. A recently published cohort study proved the association between osteoporosis and sepsis, which found that people with low BMD, particularly those with osteoporosis, were at a higher risk of infections and sepsis than those with normal BMD [44]. The above findings suggest that clinicians should not only focus on fragility fractures but also on other complications of osteoporosis, such as sepsis, among postmenopausal women. In addition, and different from the rates and risks of readmissions, the LOS was longer in obese patients than the non-obese population. The results of TOTCHG were similar to the LOS, with obese patients spending more than non-obese patients during unplanned re-hospitalization. In addition, the difference between the MUNO and MHNO subgroups was statistically significant, though it was not obvious. MUNO expended more than MHNO during the unplanned readmission of postmenopausal osteoporotic patients.

The strength of our study lies in the use of a large national readmission database (NRD). This allowed us to acquire a large-scale cohort, with a diverse range of geographical locations, different hospital systems, and socio-economic backgrounds in the US. In addition, this is the first study about the association of metabolic obesity phenotypes and readmissions among hospitalized postmenopausal patients concomitant with osteoporosis. There are several limitations in our study. The NRD’s clinical details (e.g., severity of osteoporosis, laboratory data, medications, outpatient care, and quality of life, etc.) have a lack of depth. The NRD does not provide specific demographic baseline data, including ethnicity, education levels, or geographical distribution. In addition, the NRD does not track patient data back to previous years or over consecutive years, so we excluded patients admitted in the corresponding part of the year to avoid missing data from the 30-day or 90-day readmissions of patients. As with any retrospective cohort study, our findings can only prove the correlations but not the causality.

## 5. Conclusions

Metabolic abnormalities were associated with elevated rates and risks of 30- or 90-day readmission among postmenopausal hospitalized women complicated with osteoporosis, whereas obesity was not seen to be innocent, and the combination of these led to an additional burden on healthcare systems and individuals. These findings suggest that clinicians and researchers should focus not only on weight management but also metabolism intervention among patients with postmenopausal osteoporosis. The early detection and intervention of osteoporosis play vital roles in reducing the burden of healthcare, and the establishment of an integrated pathway for bone health care is aligned with the strategy of the United Nations’ (UN) Decade of Healthy Ageing 2021–2030 [4]. Further studies are urgently needed to confirm the generalizability of our findings.

## Data Availability

The data presented in this study are available within the article or in Supplementary Materials.

## Supplementary Materials

The following supporting information can be downloaded at: https://www.mdpi.com/article/10.3390/jcm12041623/s1, Table S1: ICD-10 Codes used for inclusion, exclusion, and classification; Table S2: Baseline characteristics of study population according to different metabolic obesity phenotypes (30-day readmission); Table S3: Baseline characteristics of study population according to different metabolic obesity phenotypes (90-day readmission). Table S4: Associations between metabolic obesity phenotypes with 30-day and 90-day readmission; Table S5: Associations between single metabolic disorder phenotypes with 30-day and 90-day readmission; Table S6: Associations between the number of metabolic risk factors with 30-day and 90-day readmission; Figure S1: Prevalence of fragility fractures among different metabolic obesity phenotypes at initial admission.
