# Supplementary material for: Association between Metabolic Obesity Phenotypes and the Burden of Hospitalized Postmenopausal Patients Concomitant with Osteoporosis: A Retrospective Cohort Study Based on the National Readmission Database
Source: J Clin Med. 2023 Feb 17;12(4):1623. doi: 10.3390/jcm12041623 (PMC9959570; doi:10.3390/jcm12041623)
Supplement: Supplementary file 1 [file jcm-12-01623-s001.zip › jcm-2081137-supplementary.pdf]

Supplementary Table S1. ICD-10 Codes used for inclusion, exclusion, and classification.

Diagnoses	ICD-10-CM Codes
**Primary osteoporosis**	M8000XA, M80011A, M80012A, M80019A, M80021A, M80022A, M80029A, M80031A, M80032A, M80039A, M80041A, M80042A, M80049A, M80051A, M80052A, M80059A, M80061A, M80062A, M80069A, M80071A, M80072A, M80079A, M8008XA, M800AXA, M8080XA, M80811A, M80812A, M80819A, M80821A, M80822A, M80829A, M80831A, M80832A, M80839A, M80841A, M80842A, M80849A, M80851A, M80852A, M80859A, M80861A, M80862A, M80869A, M80871A, M80872A, M80879A, M8088XA, M808AXA, M810, M816, M818
**Pregnancy**	Z331, Z332, Z333, Z3400, Z3401, Z3402, Z3403, Z3480, Z3481, Z3482, Z3483, Z3491, Z3492, Z3493, Z36, Z360, Z361, Z362, Z363, Z364, Z365, Z3681, Z3682, Z3683, Z3684, Z3685, Z3686, Z3687, Z3688, Z3689, Z368A, Z369, Z3A00, Z3A01, Z3A08, Z3A09, Z3A10, Z3A11, Z3A12, Z3A13, Z3A14, Z3A15, Z3A16, Z3A17, Z3A19, Z3A18, Z3A20, Z3A21, Z3A22, Z3A23, Z3A24, Z3A25, Z3A26, Z3A27, Z3A28, Z3A29, Z3A30, Z3A31, Z3A32, Z3A33, Z3A34, Z3A35, Z3A36, Z3A37, Z3A38, Z3A39, Z3A40, Z3A41, Z3A42, Z3A49, O000, O0000, O0001
**Low body weight**	Z681
**Normal body weight**	Z6820, Z6821, Z6822, Z6823, Z6824
**Overweight**	Z6825, Z6826, Z6827, Z6828, Z6829, E663, DE660A, E669O
**Obesity of grade 1**	Z6830, Z6831, Z6832, Z6833, Z6834, DE660B
**Obesity of grade 2**	Z6835, Z6836, Z6837, Z6838, Z6839, DE660C
**Obesity of grade 3**	Z6841, Z6842, Z6843, Z6844, Z6845, E6601, E662, DE660E, DE660F, DE660G, DE660H
**Dyslipidemia**	Hypercholesteremia: E780, E7800, E7801<br>Hypertriglyceridemia: E781<br>Others: E782, E784, E7849, E785, E783, E7841
**Hypertension**	H35031, H35032, H35033, H35039, G932, I10, I110, I119, I120, I129, I130, I1310, I1311, I132, I150, I151, I152, I158, I159, I160, I161, I169, I674, R030, I973
**Hyperglycemia**	Prediabetes: R7301, R7302, R7303<br>T1DM: E108, E109, E1010, E1011, E1021, E1022, E1029, E10311, E10319, E10321, E103213, E103212, E103211, E103219, E10329, E103293, E103292, E103291, E103299, E10331, E103313, E103312, E103311, E103319, E10339, E103393, E103392, E103391, E103399, E10341, E103413, E103412, E103411, E103419, E10349, E103493, E103492, E103491, E103499, E10351, E103513, E103512, E103511, E103519, E103523, E103522, E103521, E103529, E103533, E103532, E103531, E103539, E103543, E103542, E103541, E103549, E103553, E103552, E103551, E103559, E10359, E103593, E103592, E103591, E103599, E1036, E1039, E1037X3, E1037X2, E1037X1, E1037X9, E1040, E1041, E1042, E1044, E1043, E1049, E1051, E1052, E1059, E10610,

	E10618, E10620, E10621, E10622, E10628, E10630, E10638, E10641, E10649, E1065, E1069<br>T2DM: E1100, E1101, E1111, E1110, E1122, E1121, E1129, E11311, E11319, E11321, E113213, E113212, E113211, E113219, E11329, E113293, E113292, E113291, E113299, E11331, E113313, E113312, E113311, E113319, E11339, E113393, E113392, E113391, E113399, E11341, E113413, E113412, E113411, E113419, E11349, E113493, E113492, E113491, E113499, E11351, E113513, E113512, E113511, E113519, E113523, E113522, E113521, E113529, E113533, E113532, E113531, E113539, E113543, E113542, E113541, E113549, E113553, E113552, E113551, E113559, E11359, E113593, E113592, E113591, E113599, E1136, E1139, E1144, E1143, E1141, E1140, E1142, E1149, E1152, E1151, E1159, E11620, E11621, E11622, E11628, E11610, E11618, E11630, E11638, E1137X3, E1137X2, E1137X1, E1137X9, E11641, E11649, E1165, E1169, E118, E119<br>Diabetic complication: E0800, E0801, E0810, E0811, E0821, E0829, E08311, E08319, E08321, E083211, E083212, E083213, E083219, E08329, E083291, E083292, E083293, E083299, E08331, E083311, E083312, E083313, E083319, E08339, E083391, E083392, E083393, E083399, E08341, E083411, E083412, E083413, E083419, E08349, E083491, E083492, E083493, E083499, E08351, E083511, E083512, E083513, E083519, E083521, E083522, E083523, E083529, E083531, E083532, E083543, E083549, E083551, E083552, E083553, E083559, E08359, E083591, E083592, E083593, E083599, E0836, E0837X1, E0837X2, E0837X3, E0837X9, E0839, E0840, E0841, E0842, E0843, E0844, E0849, E0851, E0852, E0859, E08610, E08618, E08620, E08621, E08622, E08628, E08630, E08638, E08641, E08649, E0865, E0869, E088, E089<br>Others: E1300, E1301, E1310, E1311, E1321, E1322, E1329, E13311, E13319, E13321, E133213, E133212, E133211, E133219, E13329, E133293, E133292, E133291, E133299,E13331, E133313, E133312, E133311, E133319, E13339, E133393, E133392, E133391, E133399, E13341, E133413, E133412, E133411, E133419, E13349, E133493, E133492, E133491, E133499, E13351, E133513, E133512, E133511, E133519, E133523, E133522, E133521, E133529, E133533, E133532, E133531, E133539, E133543, E133542, E133541, E133549, E133553, E133552, E133551, E133559, E13359, E133593, E133592, E133591, E133599, E1340, E1341, E1342, E1343, E1344, E1349, E1336, E1339, E1351, E1352, E1359, E13610, E13618, E13620, E13621, E13622, E13630, E13638, E13641, E13649, E1365, E1369, E1337X3, E1337X2, E1337X1, E1337X9, E138, E139
**Fragility fractures (Inclusion)**	Femur:<br>S72301A, S72301B, S72301C, S72301D, S72301E, S72301F, S72301G, S72301H, S72301J, S72301K, S72301M, S72301N, S72301P, S72301Q,

	S72301R, S72301S, S72302A, S72302B, S72302C, S72302D, S72302E, S72302F, S72302G, S72302H, S72302J, S72302K, S72302M, S72302N, S72302P, S72302Q, S72302R, S72302S, S72309A, S72309B, S72309C, S72309D, S72309E, S72309F, S72309G, S72309H, S72309J, S72309K, S72309M, S72309N, S72309P, S72309Q, S72309R, S72309S, S72321A, S72321B, S72321C, S72321D, S72321E, S72321F, S72321G, S72321H, S72321J, S72321K, S72321M, S72321N, S72321P, S72321Q, S72321R, S72321S, S72322A, S72322B, S72322C, S72322D, S72322E, S72322F, S72322G, S72322H, S72322J, S72322K, S72322M, S72322N, S72322P, S72322Q, S72322R, S72322S, S72323A, S72323B, S72323C, S72323D, S72323E, S72323F, S72323G, S72323H, S72323J, S72323K, S72323M, S72323N, S72323P, S72323Q, S72323R, S72323S, S72324A, S72324B, S72324C, S72324D, S72324E, S72324F, S72324G, S72324H, S72324J, S72324K, S72324M, S72324N, S72324P, S72324Q, S72324R, S72324S, S72325A, S72325B, S72325C, S72325D, S72325E, S72325F, S72325G, S72325H, S72325J, S72325K, S72325M, S72325N, S72325P, S72325Q, S72325R, S72325S, S72326A, S72326B, S72326C, S72326D, S72326E, S72326F, S72326G, S72326H, S72326J, S72326K, S72326M, S72326N, S72326P, S72326Q, S72326R, S72326S, S72331A, S72331B, S72331C, S72331D, S72331E, S72331F, S72331G, S72331H, S72331J, S72331K, S72331M, S72331N, S72331P, S72331Q, S72331R, S72331S, S72332A, S72332B, S72332C, S72332D, S72332E, S72332F, S72332G, S72332H, S72332J, S72332K, S72332M, S72332N, S72332P, S72332Q, S72332R, S72332S, S72333A, S72333B, S72333C, S72333D, S72333E, S72333F, S72333G, S72333H, S72333J, S72333K, S72333M, S72333N, S72333P, S72333Q, S72333R, S72333S, S72334A, S72334B, S72334C, S72334D, S72334E, S72334F, S72334G, S72334H, S72334J, S72334K, S72334M, S72334N, S72334P, S72334Q, S72334R, S72334S, S72335A, S72335B, S72335C, S72335D, S72335E, S72335F, S72335G, S72335H, S72335J, S72335K, S72335M, S72335N, S72335P, S72335Q, S72335R, S72335S, S72336A, S72336B, S72336C, S72336D, S72336E, S72336F, S72336G, S72336H, S72336J, S72336K, S72336M, S72336N, S72336P, S72336Q, S72336R, S72336S, S72341A, S72341B, S72341C, S72341D, S72341E, S72341F, S72341G, S72341H, S72341J, S72341K, S72341M, S72341N, S72341P, S72341Q, S72341R, S72341S, S72342A, S72342B, S72342C, S72342D, S72342E, S72342F, S72342G, S72342H, S72342J, S72342K, S72342M, S72342N, S72342P, S72342Q, S72342R, S72342S, S72343A, S72343B, S72343C, S72343D, S72343E, S72343F, S72343G, S72343H, S72343J, S72343K, S72343M, S72343N, S72343P, S72343Q, S72343R, S72343S, S72344A, S72344B, S72344C, S72344D, S72344E, S72344F, S72344G, S72344H, S72344J, S72344K, S72344M, S72344N, S72344P, S72344Q, S72344R, S72344S, S72345A, S72345B, S72345C, S72345D, S72345E, S72345F, S72345G, S72345H, S72345J, S72345K, S72345M, S72345N, S72345P, S72345Q, S72345R, S72345S, S72346A, S72346B,

	S72346C, S72346D, S72346E, S72346F, S72346G, S72346H, S72346J, S72346K, S72346M, S72346N, S72346P, S72346Q, S72346R, S72346S, S72351A, S72351B, S72351C, S72351D, S72351E, S72351F, S72351G, S72351H, S72351J, S72351K, S72351M, S72351N, S72351P, S72351Q, S72351R, S72351S, S72352A, S72352B, S72352C, S72352D, S72352E, S72352F, S72352G, S72352H, S72352J, S72352K, S72352M, S72352N, S72352P, S72352Q, S72352R, S72352S, S72353A, S72353B, S72353C, S72353D, S72353E, S72353F, S72353G, S72353H, S72353J, S72353K, S72353M, S72353N, S72353P, S72353Q, S72353R, S72353S, S72354A, S72354B, S72354C, S72354D, S72354E, S72354F, S72354G, S72354H, S72354J, S72354K, S72354M, S72354N, S72354P, S72354Q, S72354R, S72354S, S72355A, S72355B, S72355C, S72355D, S72355E, S72355F, S72355G, S72355H, S72355J, S72355K, S72355M, S72355N, S72355P, S72355Q, S72355R, S72355S, S72356A, S72356B, S72356C, S72356D, S72356E, S72356F, S72356G, S72356H, S72356J, S72356K, S72356M, S72356N, S72356P, S72356Q, S72356R, S72356S, S72361A, S72361B, S72361C, S72361D, S72361E, S72361F, S72361G, S72361H, S72361J, S72361K, S72361M, S72361N, S72361P, S72361Q, S72361R, S72361S, S72362A, S72362B, S72362C, S72362D, S72362E, S72362F, S72362G, S72362H, S72362J, S72362K, S72362M, S72362N, S72362P, S72362Q, S72362R, S72362S, S72363A, S72363B, S72363C, S72363D, S72363E, S72363F, S72363G, S72363H, S72363J, S72363K, S72363M, S72363N, S72363P, S72363Q, S72363R, S72363S, S72364A, S72364B, S72364C, S72364D, S72364E, S72364F, S72364G, S72364H, S72364J, S72364K, S72364M, S72364N, S72364P, S72364Q, S72364R, S72364S, S72365A, S72365B, S72365C, S72365D, S72365E, S72365F, S72365G, S72365H, S72365J, S72365K, S72365M, S72365N, S72365P, S72365Q, S72365R, S72365S, S72366A, S72366B, S72366C, S72366D, S72366E, S72366F, S72366G, S72366H, S72366J, S72366K, S72366M, S72366N, S72366P, S72366Q, S72366R, S72366S, S72391A, S72391B, S72391C, S72391D, S72391E, S72391F, S72391G, S72391H, S72391J, S72391K, S72391M, S72391N, S72391P, S72391Q, S72391R, S72391S, S72392A, S72392B, S72392C, S72392D, S72392E, S72392F, S72392G, S72392H, S72392J, S72392K, S72392M, S72392N, S72392P, S72392Q, S72392R, S72392S, S72399A, S72399B, S72399C, S72399D, S72399E, S72399F, S72399G, S72399H, S72399J, S72399K, S72399M, S72399N, S72399P, S72399Q, S72399R, S72399S, S72401A, S72401B, S72401C, S72401D, S72401E, S72401F, S72401G, S72401H, S72401J, S72401K, S72401M, S72401N, S72401P, S72401Q, S72401R, S72401S, S72402A, S72402B, S72402C, S72402D, S72402E, S72402F, S72402G, S72402H, S72402J, S72402K, S72402M, S72402N, S72402P, S72402Q, S72402R, S72402S, S72409A, S72409B, S72409C, S72409D, S72409E, S72409F, S72409G, S72409H, S72409J, S72409K, S72409M, S72409N, S72409P, S72409Q, S72409R, S72409S, S72411A, S72411B, S72411C, S72411D, S72411E, S72411F,

	S72411G, S72411H, S72411J, S72411K, S72411M, S72411N, S72411P, S72411Q, S72411R, S72411S, S72412A, S72412B, S72412C, S72412D, S72412E, S72412F, S72412G, S72412H, S72412J, S72412K, S72412M, S72412N, S72412P, S72412Q, S72412R, S72412S, S72413A, S72413B, S72413C, S72413D, S72413E, S72413F, S72413G, S72413H, S72413J, S72413K, S72413M, S72413N, S72413P, S72413Q, S72413R, S72413S, S72414A, S72414B, S72414C, S72414D, S72414E, S72414F, S72414G, S72414H, S72414J, S72414K, S72414M, S72414N, S72414P, S72414Q, S72414R, S72414S, S72415A, S72415B, S72415C, S72415D, S72415E, S72415F, S72415G, S72415H, S72415J, S72415K, S72415M, S72415N, S72415P, S72415Q, S72415R, S72415S, S72416A, S72416B, S72416C, S72416D, S72416E, S72416F, S72416G, S72416H, S72416J, S72416K, S72416M, S72416N, S72416P, S72416Q, S72416R, S72416S, S72421A, S72421B, S72421C, S72421D, S72421E, S72421F, S72421G, S72421H, S72421J, S72421K, S72421M, S72421N, S72421P, S72421Q, S72421R, S72421S, S72422A, S72422B, S72422C, S72422D, S72422E, S72422F, S72422G, S72422H, S72422J, S72422K, S72422M, S72422N, S72422P, S72422Q, S72422R, S72422S, S72423A, S72423B, S72423C, S72423D, S72423E, S72423F, S72423G, S72423H, S72423J, S72423K, S72423M, S72423N, S72423P, S72423Q, S72423R, S72423S, S72424A, S72424B, S72424C, S72424D, S72424E, S72424F, S72424G, S72424H, S72424J, S72424K, S72424M, S72424N, S72424P, S72424Q, S72424R, S72424S, S72425A, S72425B, S72425C, S72425D, S72425E, S72425F, S72425G, S72425H, S72425J, S72425K, S72425M, S72425N, S72425P, S72425Q, S72425R, S72425S, S72426A, S72426B, S72426C, S72426D, S72426E, S72426F, S72426G, S72426H, S72426J, S72426K, S72426M, S72426N, S72426P, S72426Q, S72426R, S72426S, S72431A, S72431B, S72431C, S72431D, S72431E, S72431F, S72431G, S72431H, S72431J, S72431K, S72431M, S72431N, S72431P, S72431Q, S72431R, S72431S, S72432A, S72432B, S72432C, S72432D, S72432E, S72432F, S72432G, S72432H, S72432J, S72432K, S72432M, S72432N, S72432P, S72432Q, S72432R, S72432S, S72433A, S72433B, S72433C, S72433D, S72433E, S72433F, S72433G, S72433H, S72433J, S72433K, S72433M, S72433N, S72433P, S72433Q, S72433R, S72433S, S72434A, S72434B, S72434C, S72434D, S72434E, S72434F, S72434G, S72434H, S72434J, S72434K, S72434M, S72434N, S72434P, S72434Q, S72434R, S72434S, S72435A, S72435B, S72435C, S72435D, S72435E, S72435F, S72435G, S72435H, S72435J, S72435K, S72435M, S72435N, S72435P, S72435Q, S72435R, S72435S, S72436A, S72436B, S72436C, S72436D, S72436E, S72436F, S72436G, S72436H, S72436J, S72436K, S72436M, S72436N, S72436P, S72436Q, S72436R, S72436S, S72441A, S72441B, S72441C, S72441D, S72441E, S72441F, S72441G, S72441H, S72441J, S72441K, S72441M, S72441N, S72441P, S72441Q, S72441R, S72441S, S72442A, S72442B, S72442C, S72442D, S72442E, S72442F, S72442G, S72442H, S72442J, S72442K,

	S72442M, S72442N, S72442P, S72442Q, S72442R, S72442S, S72443A, S72443B, S72443C, S72443D, S72443E, S72443F, S72443G, S72443H, S72443J, S72443K, S72443M, S72443N, S72443P, S72443Q, S72443R, S72443S, S72444A, S72444B, S72444C, S72444D, S72444E, S72444F, S72444G, S72444H, S72444J, S72444K, S72444M, S72444N, S72444P, S72444Q, S72444R, S72444S, S72445A, S72445B, S72445C, S72445D, S72445E, S72445F, S72445G, S72445H, S72445J, S72445K, S72445M, S72445N, S72445P, S72445Q, S72445R, S72445S, S72446A, S72446B, S72446C, S72446D, S72446E, S72446F, S72446G, S72446H, S72446J, S72446K, S72446M, S72446N, S72446P, S72446Q, S72446R, S72446S, S72451A, S72451B, S72451C, S72451D, S72451E, S72451F, S72451G, S72451H, S72451J, S72451K, S72451M, S72451N, S72451P, S72451Q, S72451R, S72451S, S72452A, S72452B, S72452C, S72452D, S72452E, S72452F, S72452G, S72452H, S72452J, S72452K, S72452M, S72452N, S72452P, S72452Q, S72452R, S72452S, S72453A, S72453B, S72453C, S72453D, S72453E, S72453F, S72453G, S72453H, S72453J, S72453K, S72453M, S72453N, S72453P, S72453Q, S72453R, S72453S, S72454A, S72454B, S72454C, S72454D, S72454E, S72454F, S72454G, S72454H, S72454J, S72454K, S72454M, S72454N, S72454P, S72454Q, S72454R, S72454S, S72455A, S72455B, S72455C, S72455D, S72455E, S72455F, S72455G, S72455H, S72455J, S72455K, S72455M, S72455N, S72455P, S72455Q, S72455R, S72455S, S72456A, S72456B, S72456C, S72456D, S72456E, S72456F, S72456G, S72456H, S72456J, S72456K, S72456M, S72456N, S72456P, S72456Q, S72456R, S72456S, S72461A, S72461B, S72461C, S72461D, S72461E, S72461F, S72461G, S72461H, S72461J, S72461K, S72461M, S72461N, S72461P, S72461Q, S72461R, S72461S, S72462A, S72462B, S72462C, S72462D, S72462E, S72462F, S72462G, S72462H, S72462J, S72462K, S72462M, S72462N, S72462P, S72462Q, S72462R, S72462S, S72463A, S72463B, S72463C, S72463D, S72463E, S72463F, S72463G, S72463H, S72463J, S72463K, S72463M, S72463N, S72463P, S72463Q, S72463R, S72463S, S72464A, S72464B, S72464C, S72464D, S72464E, S72464F, S72464G, S72464H, S72464J, S72464K, S72464M, S72464N, S72464P, S72464Q, S72464R, S72464S, S72465A, S72465B, S72465C, S72465D, S72465E, S72465F, S72465G, S72465H, S72465J, S72465K, S72465M, S72465N, S72465P, S72465Q, S72465R, S72465S, S72466A, S72466B, S72466C, S72466D, S72466E, S72466F, S72466G, S72466H, S72466J, S72466K, S72466M, S72466N, S72466P, S72466Q, S72466R, S72466S, S72471A, S72471D, S72471G, S72471K, S72471P, S72471S, S72472A, S72472D, S72472G, S72472K, S72472P, S72472S, S72479A, S72479D, S72479G, S72479K, S72479P, S72479S, S72491A, S72491B, S72491C, S72491D, S72491E, S72491F, S72491G, S72491H, S72491J, S72491K, S72491M, S72491N, S72491P, S72491Q, S72491R, S72491S, S72492A, S72492B, S72492C, S72492D, S72492E, S72492F, S72492G, S72492H, S72492J, S72492K, S72492M, S72492N,

	S72492P, S72492Q, S72492R, S72492S, S72499A, S72499B, S72499C, S72499D, S72499E, S72499F, S72499G, S72499H, S72499J, S72499K, S72499M, S72499N, S72499P, S72499Q, S72499R, S72499S, S728X1A, S728X1B, S728X1C, S728X1D, S728X1E, S728X1F, S728X1G, S728X1H, S728X1J, S728X1K, S728X1M, S728X1N, S728X1P, S728X1Q, S728X1R, S728X1S, S728X2A, S728X2B, S728X2C, S728X2D, S728X2E, S728X2F, S728X2G, S728X2H, S728X2J, S728X2K, S728X2M, S728X2N, S728X2P, S728X2Q, S728X2R, S728X2S, S728X9A, S728X9B, S728X9C, S728X9D, S728X9E, S728X9F, S728X9G, S728X9H, S728X9J, S728X9K, S728X9M, S728X9N, S728X9P, S728X9Q, S728X9R, S728X9S, S7290XA, S7290XB, S7290XC, S7290XD, S7290XE, S7290XF, S7290XG, S7290XH, S7290XJ, S7290XK, S7290XM, S7290XN, S7290XP, S7290XQ, S7290XR, S7290XS, S7291XA, S7291XB, S7291XC, S7291XD, S7291XE, S7291XF, S7291XG, S7291XH, S7291XJ, S7291XK, S7291XM, S7291XN, S7291XP, S7291XQ, S7291XR, S7291XS, S7292XA, S7292XB, S7292XC, S7292XD, S7292XE, S7292XF, S7292XG, S7292XH, S7292XJ, S7292XK, S7292XM, S7292XN, S7292XP, S7292XQ, S7292XR, S7292XS<br>Hip:<br>S72001A, S72001B, S72001C, S72001D, S72001E, S72001F, S72001G, S72001H, S72001J, S72001K, S72001M, S72001N, S72001P, S72001Q, S72001R, S72001S, S72002A, S72002B, S72002C, S72002D, S72002E, S72002F, S72002G, S72002H, S72002J, S72002K, S72002M, S72002N, S72002P, S72002Q, S72002R, S72002S, S72009A, S72009B, S72009C, S72009D, S72009E, S72009F, S72009G, S72009H, S72009J, S72009K, S72009M, S72009N, S72009P, S72009Q, S72009R, S72009S, S72011A, S72011B, S72011C, S72011D, S72011E, S72011F, S72011G, S72011H, S72011J, S72011K, S72011M, S72011N, S72011P, S72011Q, S72011R, S72011S, S72012A, S72012B, S72012C, S72012D, S72012E, S72012F, S72012G, S72012H, S72012J, S72012K, S72012M, S72012N, S72012P, S72012Q, S72012R, S72012S, S72019A, S72019B, S72019C, S72019D, S72019E, S72019F, S72019G, S72019H, S72019J, S72019K, S72019M, S72019N, S72019P, S72019Q, S72019R, S72019S, S72021A, S72021B, S72021C, S72021D, S72021E, S72021F, S72021G, S72021H, S72021J, S72021K, S72021M, S72021N, S72021P, S72021Q, S72021R, S72021S, S72022A, S72022B, S72022C, S72022D, S72022E, S72022F, S72022G, S72022H, S72022J, S72022K, S72022M, S72022N, S72022P, S72022Q, S72022R, S72022S, S72023A, S72023B, S72023C, S72023D, S72023E, S72023F, S72023G, S72023H, S72023J, S72023K, S72023M, S72023N, S72023P, S72023Q, S72023R, S72023S, S72024A, S72024B, S72024C, S72024D, S72024E, S72024F, S72024G, S72024H, S72024J, S72024K, S72024M, S72024N, S72024P, S72024Q, S72024R, S72024S, S72025A, S72025B, S72025C, S72025D, S72025E, S72025F, S72025G, S72025H, S72025J, S72025K, S72025M, S72025N, S72025P, S72025Q, S72025R, S72025S, S72026A, S72026B, S72026C, S72026D, S72026E, S72026F,

	S72026G, S72026H, S72026J, S72026K, S72026M, S72026N, S72026P, S72026Q, S72026R, S72026S, S72031A, S72031B, S72031C, S72031D, S72031E, S72031F, S72031G, S72031H, S72031J, S72031K, S72031M, S72031N, S72031P, S72031Q, S72031R, S72031S, S72032A, S72032B, S72032C, S72032D, S72032E, S72032F, S72032G, S72032H, S72032J, S72032K, S72032M, S72032N, S72032P, S72032Q, S72032R, S72032S, S72033A, S72033B, S72033C, S72033D, S72033E, S72033F, S72033G, S72033H, S72033J, S72033K, S72033M, S72033N, S72033P, S72033Q, S72033R, S72033S, S72034A, S72034B, S72034C, S72034D, S72034E, S72034F, S72034G, S72034H, S72034J, S72034K, S72034M, S72034N, S72034P, S72034Q, S72034R, S72034S, S72035A, S72035B, S72035C, S72035D, S72035E, S72035F, S72035G, S72035H, S72035J, S72035K, S72035M, S72035N, S72035P, S72035Q, S72035R, S72035S, S72036A, S72036B, S72036C, S72036D, S72036E, S72036F, S72036G, S72036H, S72036J, S72036K, S72036M, S72036N, S72036P, S72036Q, S72036R, S72036S, S72041A, S72041B, S72041C, S72041D, S72041E, S72041F, S72041G, S72041H, S72041J, S72041K, S72041M, S72041N, S72041P, S72041Q, S72041R, S72041S, S72042A, S72042B, S72042C, S72042D, S72042E, S72042F, S72042G, S72042H, S72042J, S72042K, S72042M, S72042N, S72042P, S72042Q, S72042R, S72042S, S72043A, S72043B, S72043C, S72043D, S72043E, S72043F, S72043G, S72043H, S72043J, S72043K, S72043M, S72043N, S72043P, S72043Q, S72043R, S72043S, S72044A, S72044B, S72044C, S72044D, S72044E, S72044F, S72044G, S72044H, S72044J, S72044K, S72044M, S72044N, S72044P, S72044Q, S72044R, S72044S, S72045A, S72045B, S72045C, S72045D, S72045E, S72045F, S72045G, S72045H, S72045J, S72045K, S72045M, S72045N, S72045P, S72045Q, S72045R, S72045S, S72046A, S72046B, S72046C, S72046D, S72046E, S72046F, S72046G, S72046H, S72046J, S72046K, S72046M, S72046N, S72046P, S72046Q, S72046R, S72046S, S72051A, S72051B, S72051C, S72051D, S72051E, S72051F, S72051G, S72051H, S72051J, S72051K, S72051M, S72051N, S72051P, S72051Q, S72051R, S72051S, S72052A, S72052B, S72052C, S72052D, S72052E, S72052F, S72052G, S72052H, S72052J, S72052K, S72052M, S72052N, S72052P, S72052Q, S72052R, S72052S, S72059A, S72059B, S72059C, S72059D, S72059E, S72059F, S72059G, S72059H, S72059J, S72059K, S72059M, S72059N, S72059P, S72059Q, S72059R, S72059S, S72061A, S72061B, S72061C, S72061D, S72061E, S72061F, S72061G, S72061H, S72061J, S72061K, S72061M, S72061N, S72061P, S72061Q, S72061R, S72061S, S72062A, S72062B, S72062C, S72062D, S72062E, S72062F, S72062G, S72062H, S72062J, S72062K, S72062M, S72062N, S72062P, S72062Q, S72062R, S72062S, S72063A, S72063B, S72063C, S72063D, S72063E, S72063F, S72063G, S72063H, S72063J, S72063K, S72063M, S72063N, S72063P, S72063Q, S72063R, S72063S, S72064A, S72064B, S72064C, S72064D, S72064E, S72064F, S72064G, S72064H, S72064J, S72064K,

	S72064M, S72064N, S72064P, S72064Q, S72064R, S72064S, S72065A, S72065B, S72065C, S72065D, S72065E, S72065F, S72065G, S72065H, S72065J, S72065K, S72065M, S72065N, S72065P, S72065Q, S72065R, S72065S, S72066A, S72066B, S72066C, S72066D, S72066E, S72066F, S72066G, S72066H, S72066J, S72066K, S72066M, S72066N, S72066P, S72066Q, S72066R, S72066S, S72091A, S72091B, S72091C, S72091D, S72091E, S72091F, S72091G, S72091H, S72091J, S72091K, S72091M, S72091N, S72091P, S72091Q, S72091R, S72091S, S72092A, S72092B, S72092C, S72092D, S72092E, S72092F, S72092G, S72092H, S72092J, S72092K, S72092M, S72092N, S72092P, S72092Q, S72092R, S72092S, S72099A, S72099B, S72099C, S72099D, S72099E, S72099F, S72099G, S72099H, S72099J, S72099K, S72099M, S72099N, S72099P, S72099Q, S72099R, S72099S, S72101A, S72101B, S72101C, S72101D, S72101E, S72101F, S72101G, S72101H, S72101J, S72101K, S72101M, S72101N, S72101P, S72101Q, S72101R, S72101S, S72102A, S72102B, S72102C, S72102D, S72102E, S72102F, S72102G, S72102H, S72102J, S72102K, S72102M, S72102N, S72102P, S72102Q, S72102R, S72102S, S72109A, S72109B, S72109C, S72109D, S72109E, S72109F, S72109G, S72109H, S72109J, S72109K, S72109M, S72109N, S72109P, S72109Q, S72109R, S72109S, S72111A, S72111B, S72111C, S72111D, S72111E, S72111F, S72111G, S72111H, S72111J, S72111K, S72111M, S72111N, S72111P, S72111Q, S72111R, S72111S, S72112A, S72112B, S72112C, S72112D, S72112E, S72112F, S72112G, S72112H, S72112J, S72112K, S72112M, S72112N, S72112P, S72112Q, S72112R, S72112S, S72113A, S72113B, S72113C, S72113D, S72113E, S72113F, S72113G, S72113H, S72113J, S72113K, S72113M, S72113N, S72113P, S72113Q, S72113R, S72113S, S72114A, S72114B, S72114C, S72114D, S72114E, S72114F, S72114G, S72114H, S72114J, S72114K, S72114M, S72114N, S72114P, S72114Q, S72114R, S72114S, S72115A, S72115B, S72115C, S72115D, S72115E, S72115F, S72115G, S72115H, S72115J, S72115K, S72115M, S72115N, S72115P, S72115Q, S72115R, S72115S, S72116A, S72116B, S72116C, S72116D, S72116E, S72116F, S72116G, S72116H, S72116J, S72116K, S72116M, S72116N, S72116P, S72116Q, S72116R, S72116S, S72121A, S72121B, S72121C, S72121D, S72121E, S72121F, S72121G, S72121H, S72121J, S72121K, S72121M, S72121N, S72121P, S72121Q, S72121R, S72121S, S72122A, S72122B, S72122C, S72122D, S72122E, S72122F, S72122G, S72122H, S72122J, S72122K, S72122M, S72122N, S72122P, S72122Q, S72122R, S72122S, S72123A, S72123B, S72123C, S72123D, S72123E, S72123F, S72123G, S72123H, S72123J, S72123K, S72123M, S72123N, S72123P, S72123Q, S72123R, S72123S, S72124A, S72124B, S72124C, S72124D, S72124E, S72124F, S72124G, S72124H, S72124J, S72124K, S72124M, S72124N, S72124P, S72124Q, S72124R, S72124S, S72125A, S72125B, S72125C, S72125D, S72125E, S72125F, S72125G, S72125H, S72125J, S72125K, S72125M, S72125N, S72125P, S72125Q,

	S72125R, S72125S, S72126A, S72126B, S72126C, S72126D, S72126E, S72126F, S72126G, S72126H, S72126J, S72126K, S72126M, S72126N, S72126P, S72126Q, S72126R, S72126S, S72131A, S72131B, S72131C, S72131D, S72131E, S72131F, S72131G, S72131H, S72131J, S72131K, S72131M, S72131N, S72131P, S72131Q, S72131R, S72131S, S72132A, S72132B, S72132C, S72132D, S72132E, S72132F, S72132G, S72132H, S72132J, S72132K, S72132M, S72132N, S72132P, S72132Q, S72132R, S72132S, S72133A, S72133B, S72133C, S72133D, S72133E, S72133F, S72133G, S72133H, S72133J, S72133K, S72133M, S72133N, S72133P, S72133Q, S72133R, S72133S, S72134A, S72134B, S72134C, S72134D, S72134E, S72134F, S72134G, S72134H, S72134J, S72134K, S72134M, S72134N, S72134P, S72134Q, S72134R, S72134S, S72135A, S72135B, S72135C, S72135D, S72135E, S72135F, S72135G, S72135H, S72135J, S72135K, S72135M, S72135N, S72135P, S72135Q, S72135R, S72135S, S72136A, S72136B, S72136C, S72136D, S72136E, S72136F, S72136G, S72136H, S72136J, S72136K, S72136M, S72136N, S72136P, S72136Q, S72136R, S72136S, S72141A, S72141B, S72141C, S72141D, S72141E, S72141F, S72141G, S72141H, S72141J, S72141K, S72141M, S72141N, S72141P, S72141Q, S72141R, S72141S, S72142A, S72142B, S72142C, S72142D, S72142E, S72142F, S72142G, S72142H, S72142J, S72142K, S72142M, S72142N, S72142P, S72142Q, S72142R, S72142S, S72143A, S72143B, S72143C, S72143D, S72143E, S72143F, S72143G, S72143H, S72143J, S72143K, S72143M, S72143N, S72143P, S72143Q, S72143R, S72143S, S72144A, S72144B, S72144C, S72144D, S72144E, S72144F, S72144G, S72144H, S72144J, S72144K, S72144M, S72144N, S72144P, S72144Q, S72144R, S72144S, S72145A, S72145B, S72145C, S72145D, S72145E, S72145F, S72145G, S72145H, S72145J, S72145K, S72145M, S72145N, S72145P, S72145Q, S72145R, S72145S, S72146A, S72146B, S72146C, S72146D, S72146E, S72146F, S72146G, S72146H, S72146J, S72146K, S72146M, S72146N, S72146P, S72146Q, S72146R, S72146S, S7221XA, S7221XB, S7221XC, S7221XD, S7221XE, S7221XF, S7221XG, S7221XH, S7221XJ, S7221XK, S7221XM, S7221XN, S7221XP, S7221XQ, S7221XR, S7221XS, S7222XA, S7222XB, S7222XC, S7222XD, S7222XE, S7222XF, S7222XG, S7222XH, S7222XJ, S7222XK, S7222XM, S7222XN, S7222XP, S7222XQ, S7222XR, S7222XS, S7223XA, S7223XB, S7223XC, S7223XD, S7223XE, S7223XF, S7223XG, S7223XH, S7223XJ, S7223XK, S7223XM, S7223XN, S7223XP, S7223XQ, S7223XR, S7223XS, S7224XA, S7224XB, S7224XC, S7224XD, S7224XE, S7224XF, S7224XG, S7224XH, S7224XJ, S7224XK, S7224XM, S7224XN, S7224XP, S7224XQ, S7224XR, S7224XS, S7225XA, S7225XB, S7225XC, S7225XD, S7225XE, S7225XF, S7225XG, S7225XH, S7225XJ, S7225XK, S7225XM, S7225XN, S7225XP, S7225XQ, S7225XR, S7225XS, S7226XA, S7226XB, S7226XC, S7226XD, S7226XE, S7226XF, S7226XG, S7226XH, S7226XJ, S7226XK, S7226XM, S7226XN, S7226XP, S7226XQ, S7226XR, S7226XS

	Humerus-shoulder: S42201A, S42201B, S42201D, S42201G, S42201K, S42201P, S42201S, S42202A, S42202B, S42202D, S42202G, S42202K, S42202P, S42202S, S42209A, S42209B, S42209D, S42209G, S42209K, S42209P, S42209S, S42211A, S42211B, S42211D, S42211G, S42211K, S42211P, S42211S, S42212A, S42212B, S42212D, S42212G, S42212K, S42212P, S42212S, S42213A, S42213B, S42213D, S42213G, S42213K, S42213P, S42213S, S42214A, S42214B, S42214D, S42214G, S42214K, S42214P, S42214S, S42215A, S42215B, S42215D, S42215G, S42215K, S42215P, S42215S, S42216A, S42216B, S42216D, S42216G, S42216K, S42216P, S42216S, S42221A, S42221B, S42221D, S42221G, S42221K, S42221P, S42221S, S42222A, S42222B, S42222D, S42222G, S42222K, S42222P, S42222S, S42223A, S42223B, S42223D, S42223G, S42223K, S42223P, S42223S, S42224A, S42224B, S42224D, S42224G, S42224K, S42224P, S42224S, S42225A, S42225B, S42225D, S42225G, S42225K, S42225P, S42225S, S42226A, S42226B, S42226D, S42226G, S42226K, S42226P, S42226S, S42231A, S42231B, S42231D, S42231G, S42231K, S42231P, S42231S, S42232A, S42232B, S42232D, S42232G, S42232K, S42232P, S42232S, S42239A, S42239B, S42239D, S42239G, S42239K, S42239P, S42239S, S42241A, S42241B, S42241D, S42241G, S42241K, S42241P, S42241S, S42242A, S42242B, S42242D, S42242G, S42242K, S42242P, S42242S, S42249A, S42249B, S42249D, S42249G, S42249K, S42249P, S42249S, S42251A, S42251B, S42251D, S42251G, S42251K, S42251P, S42251S, S42252A, S42252B, S42252D, S42252G, S42252K, S42252P, S42252S, S42253A, S42253B, S42253D, S42253G, S42253K, S42253P, S42253S, S42254A, S42254B, S42254D, S42254G, S42254K, S42254P, S42254S, S42255A, S42255B, S42255D, S42255G, S42255K, S42255P, S42255S, S42256A, S42256B, S42256D, S42256G, S42256K, S42256P, S42256S, S42261A, S42261B, S42261D, S42261G, S42261K, S42261P, S42261S, S42262A, S42262B, S42262D, S42262G, S42262K, S42262P, S42262S, S42263A, S42263B, S42263D, S42263G, S42263K, S42263P, S42263S, S42264A, S42264B, S42264D, S42264G, S42264K, S42264P, S42264S, S42265A, S42265B, S42265D, S42265G, S42265K, S42265P, S42265S, S42266A, S42266B, S42266D, S42266G, S42266K, S42266P, S42266S, S42271A, S42271D, S42271G, S42271K, S42271P, S42271S, S42272A, S42272D, S42272G, S42272K, S42272P, S42272S, S42279A, S42279D, S42279G, S42279K, S42279P, S42279S, S42291A, S42291B, S42291D, S42291G, S42291K, S42291P, S42291S, S42292A, S42292B, S42292D, S42292G, S42292K, S42292P, S42292S, S42293A, S42293B, S42293D, S42293G, S42293K, S42293P, S42293S, S42294A, S42294B, S42294D, S42294G, S42294K, S42294P, S42294S, S42295A, S42295B, S42295D, S42295G, S42295K, S42295P, S42295S, S42296A, S42296B, S42296D, S42296G, S42296K, S42296P, S42296S, S42301A, S42301B, S42301D, S42301G, S42301K, S42301P, S42301S, S42302A, S42302B, S42302D,

	S42302G, S42302K, S42302P, S42302S, S42309A, S42309B, S42309D, S42309G, S42309K, S42309P, S42309S, S42311A, S42311D, S42311G, S42311K, S42311P, S42311S, S42312A, S42312D, S42312G, S42312K, S42312P, S42312S, S42319A, S42319D, S42319G, S42319K, S42319P, S42319S, S42321A, S42321B, S42321D, S42321G, S42321K, S42321P, S42321S, S42322A, S42322B, S42322D, S42322G, S42322K, S42322P, S42322S, S42323A, S42323B, S42323D, S42323G, S42323K, S42323P, S42323S, S42324A, S42324B, S42324D, S42324G, S42324K, S42324P, S42324S, S42325A, S42325B, S42325D, S42325G, S42325K, S42325P, S42325S, S42326A, S42326B, S42326D, S42326G, S42326K, S42326P, S42326S, S42331A, S42331B, S42331D, S42331G, S42331K, S42331P, S42331S, S42332A, S42332B, S42332D, S42332G, S42332K, S42332P, S42332S, S42333A, S42333B, S42333D, S42333G, S42333K, S42333P, S42333S, S42334A, S42334B, S42334D, S42334G, S42334K, S42334P, S42334S, S42335A, S42335B, S42335D, S42335G, S42335K, S42335P, S42335S, S42336A, S42336B, S42336D, S42336G, S42336K, S42336P, S42336S, S42341A, S42341B, S42341D, S42341G, S42341K, S42341P, S42341S, S42342A, S42342B, S42342D, S42342G, S42342K, S42342P, S42342S, S42343A, S42343B, S42343D, S42343G, S42343K, S42343P, S42343S, S42344A, S42344B, S42344D, S42344G, S42344K, S42344P, S42344S, S42345A, S42345B, S42345D, S42345G, S42345K, S42345P, S42345S, S42346A, S42346B, S42346D, S42346G, S42346K, S42346P, S42346S, S42351A, S42351B, S42351D, S42351G, S42351K, S42351P, S42351S, S42352A, S42352B, S42352D, S42352G, S42352K, S42352P, S42352S, S42353A, S42353B, S42353D, S42353G, S42353K, S42353P, S42353S, S42354A, S42354B, S42354D, S42354G, S42354K, S42354P, S42354S, S42355A, S42355B, S42355D, S42355G, S42355K, S42355P, S42355S, S42356A, S42356B, S42356D, S42356G, S42356K, S42356P, S42356S, S42361A, S42361B, S42361D, S42361G, S42361K, S42361P, S42361S, S42362A, S42362B, S42362D, S42362G, S42362K, S42362P, S42362S, S42363A, S42363B, S42363D, S42363G, S42363K, S42363P, S42363S, S42364A, S42364B, S42364D, S42364G, S42364K, S42364P, S42364S, S42365A, S42365B, S42365D, S42365G, S42365K, S42365P, S42365S, S42366A, S42366B, S42366D, S42366G, S42366K, S42366P, S42366S, S42391A, S42391B, S42391D, S42391G, S42391K, S42391P, S42391S, S42392A, S42392B, S42392D, S42392G, S42392K, S42392P, S42392S, S42399A, S42399B, S42399D, S42399G, S42399K, S42399P, S42399S, S42401A, S42401B, S42401D, S42401G, S42401K, S42401P, S42401S, S42402A, S42402B, S42402D, S42402G, S42402K, S42402P, S42402S, S42409A, S42409B, S42409D, S42409G, S42409K, S42409P, S42409S, S42411A, S42411B, S42411D, S42411G, S42411K, S42411P, S42411S, S42412A, S42412B, S42412D, S42412G, S42412K, S42412P, S42412S, S42413A, S42413B, S42413D, S42413G, S42413K, S42413P, S42413S, S42414A, S42414B, S42414D, S42414G, S42414K, S42414P,

	S42414S, S42415A, S42415B, S42415D, S42415G, S42415K, S42415P, S42415S, S42416A, S42416B, S42416D, S42416G, S42416K, S42416P, S42416S, S42421A, S42421B, S42421D, S42421G, S42421K, S42421P, S42421S, S42422A, S42422B, S42422D, S42422G, S42422K, S42422P, S42422S, S42423A, S42423B, S42423D, S42423G, S42423K, S42423P, S42423S, S42424A, S42424B, S42424D, S42424G, S42424K, S42424P, S42424S, S42425A, S42425B, S42425D, S42425G, S42425K, S42425P, S42425S, S42426A, S42426B, S42426D, S42426G, S42426K, S42426P, S42426S, S42431A, S42431B, S42431D, S42431G, S42431K, S42431P, S42431S, S42432A, S42432B, S42432D, S42432G, S42432K, S42432P, S42432S, S42433A, S42433B, S42433D, S42433G, S42433K, S42433P, S42433S, S42434A, S42434B, S42434D, S42434G, S42434K, S42434P, S42434S, S42435A, S42435B, S42435D, S42435G, S42435K, S42435P, S42435S, S42436A, S42436B, S42436D, S42436G, S42436K, S42436P, S42436S, S42441A, S42441B, S42441D, S42441G, S42441K, S42441P, S42441S, S42442A, S42442B, S42442D, S42442G, S42442K, S42442P, S42442S, S42443A, S42443B, S42443D, S42443G, S42443K, S42443P, S42443S, S42444A, S42444B, S42444D, S42444G, S42444K, S42444P, S42444S, S42445A, S42445B, S42445D, S42445G, S42445K, S42445P, S42445S, S42446A, S42446B, S42446D, S42446G, S42446K, S42446P, S42446S, S42447A, S42447B, S42447D, S42447G, S42447K, S42447P, S42447S, S42448A, S42448B, S42448D, S42448G, S42448K, S42448P, S42448S, S42449A, S42449B, S42449D, S42449G, S42449K, S42449P, S42449S, S42451A, S42451B, S42451D, S42451G, S42451K, S42451P, S42451S, S42452A, S42452B, S42452D, S42452G, S42452K, S42452P, S42452S, S42453A, S42453B, S42453D, S42453G, S42453K, S42453P, S42453S, S42454A, S42454B, S42454D, S42454G, S42454K, S42454P, S42454S, S42455A, S42455B, S42455D, S42455G, S42455K, S42455P, S42455S, S42456A, S42456B, S42456D, S42456G, S42456K, S42456P, S42456S, S42461A, S42461B, S42461D, S42461G, S42461K, S42461P, S42461S, S42462A, S42462B, S42462D, S42462G, S42462K, S42462P, S42462S, S42463A, S42463B, S42463D, S42463G, S42463K, S42463P, S42463S, S42464A, S42464B, S42464D, S42464G, S42464K, S42464P, S42464S, S42465A, S42465B, S42465D, S42465G, S42465K, S42465P, S42465S, S42466A, S42466B, S42466D, S42466G, S42466K, S42466P, S42466S, S42471A, S42471B, S42471D, S42471G, S42471K, S42471P, S42471S, S42472A, S42472B, S42472D, S42472G, S42472K, S42472P, S42472S, S42473A, S42473B, S42473D, S42473G, S42473K, S42473P, S42473S, S42474A, S42474B, S42474D, S42474G, S42474K, S42474P, S42474S, S42475A, S42475B, S42475D, S42475G, S42475K, S42475P, S42475S, S42476A, S42476B, S42476D, S42476G, S42476K, S42476P, S42476S, S42481A, S42481D, S42481G, S42481K, S42481P, S42481S, S42482A, S42482D, S42482G, S42482K, S42482P, S42482S, S42489A, S42489D, S42489G, S42489K, S42489P, S42489S, S42491A, S42491B,

S42491D, S42491G, S42491K, S42491P, S42491S, S42492A, S42492B, S42492D, S42492G, S42492K, S42492P, S42492S, S42493A, S42493B, S42493D, S42493G, S42493K, S42493P, S42493S, S42494A, S42494B, S42494D, S42494G, S42494K, S42494P, S42494S, S42495A, S42495B, S42495D, S42495G, S42495K, S42495P, S42495S, S42496A, S42496B, S42496D, S42496G, S42496K, S42496P, S42496S, S4290XA, S4290XB, S4290XD, S4290XG, S4290XK, S4290XP, S4290XS, S4291XA, S4291XB, S4291XD, S4291XG, S4291XK, S4291XP, S4291XS, S4292XA, S4292XB, S4292XD, S4292XG, S4292XK, S4292XP, S4292XS

Pelvis:

S3210XA, S3210XB, S3210XD, S3210XG, S3210XK, S3210XS, S32110A, S32110B, S32110D, S32110G, S32110K, S32110S, S32111A, S32111B, S32111D, S32111G, S32111K, S32111S, S32112A, S32112B, S32112D, S32112G, S32112K, S32112S, S32119A, S32119B, S32119D, S32119G, S32119K, S32119S, S32120A, S32120B, S32120D, S32120G, S32120K, S32120S, S32121A, S32121B, S32121D, S32121G, S32121K, S32121S, S32122A, S32122B, S32122D, S32122G, S32122K, S32122S, S32129A, S32129B, S32129D, S32129G, S32129K, S32129S, S32130A, S32130B, S32130D, S32130G, S32130K, S32130S, S32131A, S32131B, S32131D, S32131G, S32131K, S32131S, S32132A, S32132B, S32132D, S32132G, S32132K, S32132S, S32139A, S32139B, S32139D, S32139G, S32139K, S32139S, S3214XA, S3214XB, S3214XD, S3214XG, S3214XK, S3214XS, S3215XA, S3215XB, S3215XD, S3215XG, S3215XK, S3215XS, S3216XA, S3216XB, S3216XD, S3216XG, S3216XK, S3216XS, S3217XA, S3217XB, S3217XD, S3217XG, S3217XK, S3217XS, S3219XA, S3219XB, S3219XD, S3219XG, S3219XK, S3219XS, S322XXA, S322XXB, S322XXD, S322XXG, S322XXK, S322XXS, S32301A, S32301B, S32301D, S32301G, S32301K, S32301S, S32302A, S32302B, S32302D, S32302G, S32302K, S32302S, S32309A, S32309B, S32309D, S32309G, S32309K, S32309S, S32311A, S32311B, S32311D, S32311G, S32311K, S32311S, S32312A, S32312B, S32312D, S32312G, S32312K, S32312S, S32313A, S32313B, S32313D, S32313G, S32313K, S32313S, S32314A, S32314B, S32314D, S32314G, S32314K, S32314S, S32315A, S32315B, S32315D, S32315G, S32315K, S32315S, S32316A, S32316B, S32316D, S32316G, S32316K, S32316S, S32391A, S32391B, S32391D, S32391G, S32391K, S32391S, S32392A, S32392B, S32392D, S32392G, S32392K, S32392S, S32399A, S32399B, S32399D, S32399G, S32399K, S32399S, S32401A, S32401B, S32401D, S32401G, S32401K, S32401S, S32402A, S32402B, S32402D, S32402G, S32402K, S32402S, S32409A, S32409B, S32409D, S32409G, S32409K, S32409S, S32411A, S32411B, S32411D, S32411G, S32411K, S32411S, S32412A, S32412B, S32412D, S32412G, S32412K, S32412S, S32413A, S32413B, S32413D, S32413G, S32413K, S32413S, S32414A, S32414B, S32414D, S32414G, S32414K, S32414S, S32415A, S32415B, S32415D, S32415G, S32415K, S32415S, S32416A, S32416B, S32416D,

	S32416G, S32416K, S32416S, S32421A, S32421B, S32421D, S32421G, S32421K, S32421S, S32422A, S32422B, S32422D, S32422G, S32422K, S32422S, S32423A, S32423B, S32423D, S32423G, S32423K, S32423S, S32424A, S32424B, S32424D, S32424G, S32424K, S32424S, S32425A, S32425B, S32425D, S32425G, S32425K, S32425S, S32426A, S32426B, S32426D, S32426G, S32426K, S32426S, S32431A, S32431B, S32431D, S32431G, S32431K, S32431S, S32432A, S32432B, S32432D, S32432G, S32432K, S32432S, S32433A, S32433B, S32433D, S32433G, S32433K, S32433S, S32434A, S32434B, S32434D, S32434G, S32434K, S32434S, S32435A, S32435B, S32435D, S32435G, S32435K, S32435S, S32436A, S32436B, S32436D, S32436G, S32436K, S32436S, S32441A, S32441B, S32441D, S32441G, S32441K, S32441S, S32442A, S32442B, S32442D, S32442G, S32442K, S32442S, S32443A, S32443B, S32443D, S32443G, S32443K, S32443S, S32444A, S32444B, S32444D, S32444G, S32444K, S32444S, S32445A, S32445B, S32445D, S32445G, S32445K, S32445S, S32446A, S32446B, S32446D, S32446G, S32446K, S32446S, S32451A, S32451B, S32451D, S32451G, S32451K, S32451S, S32452A, S32452B, S32452D, S32452G, S32452K, S32452S, S32453A, S32453B, S32453D, S32453G, S32453K, S32453S, S32454A, S32454B, S32454D, S32454G, S32454K, S32454S, S32455A, S32455B, S32455D, S32455G, S32455K, S32455S, S32456A, S32456B, S32456D, S32456G, S32456K, S32456S, S32461A, S32461B, S32461D, S32461G, S32461K, S32461S, S32462A, S32462B, S32462D, S32462G, S32462K, S32462S, S32463A, S32463B, S32463D, S32463G, S32463K, S32463S, S32464A, S32464B, S32464D, S32464G, S32464K, S32464S, S32465A, S32465B, S32465D, S32465G, S32465K, S32465S, S32466A, S32466B, S32466D, S32466G, S32466K, S32466S, S32471A, S32471B, S32471D, S32471G, S32471K, S32471S, S32472A, S32472B, S32472D, S32472G, S32472K, S32472S, S32473A, S32473B, S32473D, S32473G, S32473K, S32473S, S32474A, S32474B, S32474D, S32474G, S32474K, S32474S, S32475A, S32475B, S32475D, S32475G, S32475K, S32475S, S32476A, S32476B, S32476D, S32476G, S32476K, S32476S, S32481A, S32481B, S32481D, S32481G, S32481K, S32481S, S32482A, S32482B, S32482D, S32482G, S32482K, S32482S, S32483A, S32483B, S32483D, S32483G, S32483K, S32483S, S32484A, S32484B, S32484D, S32484G, S32484K, S32484S, S32485A, S32485B, S32485D, S32485G, S32485K, S32485S, S32486A, S32486B, S32486D, S32486G, S32486K, S32486S, S32491A, S32491B, S32491D, S32491G, S32491K, S32491S, S32492A, S32492B, S32492D, S32492G, S32492K, S32492S, S32499A, S32499B, S32499D, S32499G, S32499K, S32499S, S32501A, S32501B, S32501D, S32501G, S32501K, S32501S, S32502A, S32502B, S32502D, S32502G, S32502K, S32502S, S32509A, S32509B, S32509D, S32509G, S32509K, S32509S, S32511A, S32511B, S32511D, S32511G, S32511K, S32511S, S32512A, S32512B, S32512D, S32512G, S32512K, S32512S, S32519A, S32519B, S32519D, S32519G, S32519K,

	S32519S, S32591A, S32591B, S32591D, S32591G, S32591K, S32591S, S32592A, S32592B, S32592D, S32592G, S32592K, S32592S, S32599A, S32599B, S32599D, S32599G, S32599K, S32599S, S32810A, S32810B, S32810D, S32810G, S32810K, S32810S, S32811A, S32811B, S32811D, S32811G, S32811K, S32811S, S3282XA, S3282XB, S3282XD, S3282XG, S3282XK, S3282XS, S3289XA, S3289XB, S3289XD, S3289XG, S3289XK, S3289XS, S32601A, S32601B, S32601D, S32601G, S32601K, S32601S, S32602A, S32602B, S32602D, S32602G, S32602K, S32602S, S32609A, S32609B, S32609D, S32609G, S32609K, S32609S, S32611A, S32611B, S32611D, S32611G, S32611K, S32611S, S32612A, S32612B, S32612D, S32612G, S32612K, S32612S, S32613A, S32613B, S32613D, S32613G, S32613K, S32613S, S32614A, S32614B, S32614D, S32614G, S32614K, S32614S, S32615A, S32615B, S32615D, S32615G, S32615K, S32615S, S32616A, S32616B, S32616D, S32616G, S32616K, S32616S, S32691A, S32691B, S32691D, S32691G, S32691K, S32691S, S32692A, S32692B, S32692D, S32692G, S32692K, S32692S, S32699A, S32699B, S32699D, S32699G, S32699K, S32699S, S329XXA, S329XXB, S329XXD, S329XXG, S329XXK, S329XXS<br>Radius and ulna:<br>S52001A, S52001B, S52001C, S52001D, S52001E, S52001F, S52001G, S52001H, S52001J, S52001K, S52001M, S52001N, S52001P, S52001Q, S52001R, S52001S, S52002A, S52002B, S52002C, S52002D, S52002E, S52002F, S52002G, S52002H, S52002J, S52002K, S52002M, S52002N, S52002P, S52002Q, S52002R, S52002S, S52009A, S52009B, S52009C, S52009D, S52009E, S52009F, S52009G, S52009H, S52009J, S52009K, S52009M, S52009N, S52009P, S52009Q, S52009R, S52009S, S52011A, S52011D, S52011G, S52011K, S52011P, S52011S, S52012A, S52012D, S52012G, S52012K, S52012P, S52012S, S52019A, S52019D, S52019G, S52019K, S52019P, S52019S, S52021A, S52021B, S52021C, S52021D, S52021E, S52021F, S52021G, S52021H, S52021J, S52021K, S52021M, S52021N, S52021P, S52021Q, S52021R, S52021S, S52022A, S52022B, S52022C, S52022D, S52022E, S52022F, S52022G, S52022H, S52022J, S52022K, S52022M, S52022N, S52022P, S52022Q, S52022R, S52022S, S52023A, S52023B, S52023C, S52023D, S52023E, S52023F, S52023G, S52023H, S52023J, S52023K, S52023M, S52023N, S52023P, S52023Q, S52023R, S52023S, S52024A, S52024B, S52024C, S52024D, S52024E, S52024F, S52024G, S52024H, S52024J, S52024K, S52024M, S52024N, S52024P, S52024Q, S52024R, S52024S, S52025A, S52025B, S52025C, S52025D, S52025E, S52025F, S52025G, S52025H, S52025J, S52025K, S52025M, S52025N, S52025P, S52025Q, S52025R, S52025S, S52026A, S52026B, S52026C, S52026D, S52026E, S52026F, S52026G, S52026H, S52026J, S52026K, S52026M, S52026N, S52026P, S52026Q, S52026R, S52026S, S52031A, S52031B, S52031C, S52031D, S52031E, S52031F, S52031G, S52031H, S52031J, S52031K, S52031M, S52031N, S52031P,

	S52031Q, S52031R, S52031S, S52032A, S52032B, S52032C, S52032D, S52032E, S52032F, S52032G, S52032H, S52032J, S52032K, S52032M, S52032N, S52032P, S52032Q, S52032R, S52032S, S52033A, S52033B, S52033C, S52033D, S52033E, S52033F, S52033G, S52033H, S52033J, S52033K, S52033M, S52033N, S52033P, S52033Q, S52033R, S52033S, S52034A, S52034B, S52034C, S52034D, S52034E, S52034F, S52034G, S52034H, S52034J, S52034K, S52034M, S52034N, S52034P, S52034Q, S52034R, S52034S, S52035A, S52035B, S52035C, S52035D, S52035E, S52035F, S52035G, S52035H, S52035J, S52035K, S52035M, S52035N, S52035P, S52035Q, S52035R, S52035S, S52036A, S52036B, S52036C, S52036D, S52036E, S52036F, S52036G, S52036H, S52036J, S52036K, S52036M, S52036N, S52036P, S52036Q, S52036R, S52036S, S52041A, S52041B, S52041C, S52041D, S52041E, S52041F, S52041G, S52041H, S52041J, S52041K, S52041M, S52041N, S52041P, S52041Q, S52041R, S52041S, S52042A, S52042B, S52042C, S52042D, S52042E, S52042F, S52042G, S52042H, S52042J, S52042K, S52042M, S52042N, S52042P, S52042Q, S52042R, S52042S, S52043A, S52043B, S52043C, S52043D, S52043E, S52043F, S52043G, S52043H, S52043J, S52043K, S52043M, S52043N, S52043P, S52043Q, S52043R, S52043S, S52044A, S52044B, S52044C, S52044D, S52044E, S52044F, S52044G, S52044H, S52044J, S52044K, S52044M, S52044N, S52044P, S52044Q, S52044R, S52044S, S52045A, S52045B, S52045C, S52045D, S52045E, S52045F, S52045G, S52045H, S52045J, S52045K, S52045M, S52045N, S52045P, S52045Q, S52045R, S52045S, S52046A, S52046B, S52046C, S52046D, S52046E, S52046F, S52046G, S52046H, S52046J, S52046K, S52046M, S52046N, S52046P, S52046Q, S52046R, S52046S, S52091A, S52091B, S52091C, S52091D, S52091E, S52091F, S52091G, S52091H, S52091J, S52091K, S52091M, S52091N, S52091P, S52091Q, S52091R, S52091S, S52092A, S52092B, S52092C, S52092D, S52092E, S52092F, S52092G, S52092H, S52092J, S52092K, S52092M, S52092N, S52092P, S52092Q, S52092R, S52092S, S52099A, S52099B, S52099C, S52099D, S52099E, S52099F, S52099G, S52099H, S52099J, S52099K, S52099M, S52099N, S52099P, S52099Q, S52099R, S52099S, S52101A, S52101B, S52101C, S52101D, S52101E, S52101F, S52101G, S52101H, S52101J, S52101K, S52101M, S52101N, S52101P, S52101Q, S52101R, S52101S, S52102A, S52102B, S52102C, S52102D, S52102E, S52102F, S52102G, S52102H, S52102J, S52102K, S52102M, S52102N, S52102P, S52102Q, S52102R, S52102S, S52109A, S52109B, S52109C, S52109D, S52109E, S52109F, S52109G, S52109H, S52109J, S52109K, S52109M, S52109N, S52109P, S52109Q, S52109R, S52109S, S52111A, S52111D, S52111G, S52111K, S52111P, S52111S, S52112A, S52112D, S52112G, S52112K, S52112P, S52112S, S52119A, S52119D, S52119G, S52119K, S52119P, S52119S, S52121A, S52121B, S52121C, S52121D, S52121E, S52121F, S52121G, S52121H, S52121J, S52121K, S52121M, S52121N, S52121P, S52121Q, S52121R,

	S52121S, S52122A, S52122B, S52122C, S52122D, S52122E, S52122F, S52122G, S52122H, S52122J, S52122K, S52122M, S52122N, S52122P, S52122Q, S52122R, S52122S, S52123A, S52123B, S52123C, S52123D, S52123E, S52123F, S52123G, S52123H, S52123J, S52123K, S52123M, S52123N, S52123P, S52123Q, S52123R, S52123S, S52124A, S52124B, S52124C, S52124D, S52124E, S52124F, S52124G, S52124H, S52124J, S52124K, S52124M, S52124N, S52124P, S52124Q, S52124R, S52124S, S52125A, S52125B, S52125C, S52125D, S52125E, S52125F, S52125G, S52125H, S52125J, S52125K, S52125M, S52125N, S52125P, S52125Q, S52125R, S52125S, S52126A, S52126B, S52126C, S52126D, S52126E, S52126F, S52126G, S52126H, S52126J, S52126K, S52126M, S52126N, S52126P, S52126Q, S52126R, S52126S, S52131A, S52131B, S52131C, S52131D, S52131E, S52131F, S52131G, S52131H, S52131J, S52131K, S52131M, S52131N, S52131P, S52131Q, S52131R, S52131S, S52132A, S52132B, S52132C, S52132D, S52132E, S52132F, S52132G, S52132H, S52132J, S52132K, S52132M, S52132N, S52132P, S52132Q, S52132R, S52132S, S52133A, S52133B, S52133C, S52133D, S52133E, S52133F, S52133G, S52133H, S52133J, S52133K, S52133M, S52133N, S52133P, S52133Q, S52133R, S52133S, S52134A, S52134B, S52134C, S52134D, S52134E, S52134F, S52134G, S52134H, S52134J, S52134K, S52134M, S52134N, S52134P, S52134Q, S52134R, S52134S, S52135A, S52135B, S52135C, S52135D, S52135E, S52135F, S52135G, S52135H, S52135J, S52135K, S52135M, S52135N, S52135P, S52135Q, S52135R, S52135S, S52136A, S52136B, S52136C, S52136D, S52136E, S52136F, S52136G, S52136H, S52136J, S52136K, S52136M, S52136N, S52136P, S52136Q, S52136R, S52136S, S52181A, S52181B, S52181C, S52181D, S52181E, S52181F, S52181G, S52181H, S52181J, S52181K, S52181M, S52181N, S52181P, S52181Q, S52181R, S52181S, S52182A, S52182B, S52182C, S52182D, S52182E, S52182F, S52182G, S52182H, S52182J, S52182K, S52182M, S52182N, S52182P, S52182Q, S52182R, S52182S, S52189A, S52189B, S52189C, S52189D, S52189E, S52189F, S52189G, S52189H, S52189J, S52189K, S52189M, S52189N, S52189P, S52189Q, S52189R, S52189S, S52201A, S52201B, S52201C, S52201D, S52201E, S52201F, S52201G, S52201H, S52201J, S52201K, S52201M, S52201N, S52201P, S52201Q, S52201R, S52201S, S52202A, S52202B, S52202C, S52202D, S52202E, S52202F, S52202G, S52202H, S52202J, S52202K, S52202M, S52202N, S52202P, S52202Q, S52202R, S52202S, S52209A, S52209B, S52209C, S52209D, S52209E, S52209F, S52209G, S52209H, S52209J, S52209K, S52209M, S52209N, S52209P, S52209Q, S52209R, S52209S, S52211A, S52211D, S52211G, S52211K, S52211P, S52211S, S52212A, S52212D, S52212G, S52212K, S52212P, S52212S, S52219A, S52219D, S52219G, S52219K, S52219P, S52219S, S52221A, S52221B, S52221C, S52221D, S52221E, S52221F, S52221G, S52221H, S52221J, S52221K, S52221M, S52221N, S52221P, S52221Q, S52221R, S52221S, S52222A,

	S52222B, S52222C, S52222D, S52222E, S52222F, S52222G, S52222H, S52222J, S52222K, S52222M, S52222N, S52222P, S52222Q, S52222R, S52222S, S52223A, S52223B, S52223C, S52223D, S52223E, S52223F, S52223G, S52223H, S52223J, S52223K, S52223M, S52223N, S52223P, S52223Q, S52223R, S52223S, S52224A, S52224B, S52224C, S52224D, S52224E, S52224F, S52224G, S52224H, S52224J, S52224K, S52224M, S52224N, S52224P, S52224Q, S52224R, S52224S, S52225A, S52225B, S52225C, S52225D, S52225E, S52225F, S52225G, S52225H, S52225J, S52225K, S52225M, S52225N, S52225P, S52225Q, S52225R, S52225S, S52226A, S52226B, S52226C, S52226D, S52226E, S52226F, S52226G, S52226H, S52226J, S52226K, S52226M, S52226N, S52226P, S52226Q, S52226R, S52226S, S52231A, S52231B, S52231C, S52231D, S52231E, S52231F, S52231G, S52231H, S52231J, S52231K, S52231M, S52231N, S52231P, S52231Q, S52231R, S52231S, S52232A, S52232B, S52232C, S52232D, S52232E, S52232F, S52232G, S52232H, S52232J, S52232K, S52232M, S52232N, S52232P, S52232Q, S52232R, S52232S, S52233A, S52233B, S52233C, S52233D, S52233E, S52233F, S52233G, S52233H, S52233J, S52233K, S52233M, S52233N, S52233P, S52233Q, S52233R, S52233S, S52234A, S52234B, S52234C, S52234D, S52234E, S52234F, S52234G, S52234H, S52234J, S52234K, S52234M, S52234N, S52234P, S52234Q, S52234R, S52234S, S52235A, S52235B, S52235C, S52235D, S52235E, S52235F, S52235G, S52235H, S52235J, S52235K, S52235M, S52235N, S52235P, S52235Q, S52235R, S52235S, S52236A, S52236B, S52236C, S52236D, S52236E, S52236F, S52236G, S52236H, S52236J, S52236K, S52236M, S52236N, S52236P, S52236Q, S52236R, S52236S, S52241A, S52241B, S52241C, S52241D, S52241E, S52241F, S52241G, S52241H, S52241J, S52241K, S52241M, S52241N, S52241P, S52241Q, S52241R, S52241S, S52242A, S52242B, S52242C, S52242D, S52242E, S52242F, S52242G, S52242H, S52242J, S52242K, S52242M, S52242N, S52242P, S52242Q, S52242R, S52242S, S52243A, S52243B, S52243C, S52243D, S52243E, S52243F, S52243G, S52243H, S52243J, S52243K, S52243M, S52243N, S52243P, S52243Q, S52243R, S52243S, S52244A, S52244B, S52244C, S52244D, S52244E, S52244F, S52244G, S52244H, S52244J, S52244K, S52244M, S52244N, S52244P, S52244Q, S52244R, S52244S, S52245A, S52245B, S52245C, S52245D, S52245E, S52245F, S52245G, S52245H, S52245J, S52245K, S52245M, S52245N, S52245P, S52245Q, S52245R, S52245S, S52246A, S52246B, S52246C, S52246D, S52246E, S52246F, S52246G, S52246H, S52246J, S52246K, S52246M, S52246N, S52246P, S52246Q, S52246R, S52246S, S52251A, S52251B, S52251C, S52251D, S52251E, S52251F, S52251G, S52251H, S52251J, S52251K, S52251M, S52251N, S52251P, S52251Q, S52251R, S52251S, S52252A, S52252B, S52252C, S52252D, S52252E, S52252F, S52252G, S52252H, S52252J, S52252K, S52252M, S52252N, S52252P, S52252Q, S52252R, S52252S, S52253A, S52253B, S52253C, S52253D, S52253E,

	S52253F, S52253G, S52253H, S52253J, S52253K, S52253M, S52253N, S52253P, S52253Q, S52253R, S52253S, S52254A, S52254B, S52254C, S52254D, S52254E, S52254F, S52254G, S52254H, S52254J, S52254K, S52254M, S52254N, S52254P, S52254Q, S52254R, S52254S, S52255A, S52255B, S52255C, S52255D, S52255E, S52255F, S52255G, S52255H, S52255J, S52255K, S52255M, S52255N, S52255P, S52255Q, S52255R, S52255S, S52256A, S52256B, S52256C, S52256D, S52256E, S52256F, S52256G, S52256H, S52256J, S52256K, S52256M, S52256N, S52256P, S52256Q, S52256R, S52256S, S52261A, S52261B, S52261C, S52261D, S52261E, S52261F, S52261G, S52261H, S52261J, S52261K, S52261M, S52261N, S52261P, S52261Q, S52261R, S52261S, S52262A, S52262B, S52262C, S52262D, S52262E, S52262F, S52262G, S52262H, S52262J, S52262K, S52262M, S52262N, S52262P, S52262Q, S52262R, S52262S, S52263A, S52263B, S52263C, S52263D, S52263E, S52263F, S52263G, S52263H, S52263J, S52263K, S52263M, S52263N, S52263P, S52263Q, S52263R, S52263S, S52264A, S52264B, S52264C, S52264D, S52264E, S52264F, S52264G, S52264H, S52264J, S52264K, S52264M, S52264N, S52264P, S52264Q, S52264R, S52264S, S52265A, S52265B, S52265C, S52265D, S52265E, S52265F, S52265G, S52265H, S52265J, S52265K, S52265M, S52265N, S52265P, S52265Q, S52265R, S52265S, S52266A, S52266B, S52266C, S52266D, S52266E, S52266F, S52266G, S52266H, S52266J, S52266K, S52266M, S52266N, S52266P, S52266Q, S52266R, S52266S, S52271A, S52271B, S52271C, S52271D, S52271E, S52271F, S52271G, S52271H, S52271J, S52271K, S52271M, S52271N, S52271P, S52271Q, S52271R, S52271S, S52272A, S52272B, S52272C, S52272D, S52272E, S52272F, S52272G, S52272H, S52272J, S52272K, S52272M, S52272N, S52272P, S52272Q, S52272R, S52272S, S52279A, S52279B, S52279C, S52279D, S52279E, S52279F, S52279G, S52279H, S52279J, S52279K, S52279M, S52279N, S52279P, S52279Q, S52279R, S52279S, S52281A, S52281B, S52281C, S52281D, S52281E, S52281F, S52281G, S52281H, S52281J, S52281K, S52281M, S52281N, S52281P, S52281Q, S52281R, S52281S, S52282A, S52282B, S52282C, S52282D, S52282E, S52282F, S52282G, S52282H, S52282J, S52282K, S52282M, S52282N, S52282P, S52282Q, S52282R, S52282S, S52283A, S52283B, S52283C, S52283D, S52283E, S52283F, S52283G, S52283H, S52283J, S52283K, S52283M, S52283N, S52283P, S52283Q, S52283R, S52283S, S52291A, S52291B, S52291C, S52291D, S52291E, S52291F, S52291G, S52291H, S52291J, S52291K, S52291M, S52291N, S52291P, S52291Q, S52291R, S52291S, S52292A, S52292B, S52292C, S52292D, S52292E, S52292F, S52292G, S52292H, S52292J, S52292K, S52292M, S52292N, S52292P, S52292Q, S52292R, S52292S, S52299A, S52299B, S52299C, S52299D, S52299E, S52299F, S52299G, S52299H, S52299J, S52299K, S52299M, S52299N, S52299P, S52299Q, S52299R, S52299S, S52301A, S52301B, S52301C, S52301D, S52301E, S52301F, S52301G, S52301H, S52301J,

	S52301K, S52301M, S52301N, S52301P, S52301Q, S52301R, S52301S, S52302A, S52302B, S52302C, S52302D, S52302E, S52302F, S52302G, S52302H, S52302J, S52302K, S52302M, S52302N, S52302P, S52302Q, S52302R, S52302S, S52309A, S52309B, S52309C, S52309D, S52309E, S52309F, S52309G, S52309H, S52309J, S52309K, S52309M, S52309N, S52309P, S52309Q, S52309R, S52309S, S52311A, S52311D, S52311G, S52311K, S52311P, S52311S, S52312A, S52312D, S52312G, S52312K, S52312P, S52312S, S52319A, S52319D, S52319G, S52319K, S52319P, S52319S, S52321A, S52321B, S52321C, S52321D, S52321E, S52321F, S52321G, S52321H, S52321J, S52321K, S52321M, S52321N, S52321P, S52321Q, S52321R, S52321S, S52322A, S52322B, S52322C, S52322D, S52322E, S52322F, S52322G, S52322H, S52322J, S52322K, S52322M, S52322N, S52322P, S52322Q, S52322R, S52322S, S52323A, S52323B, S52323C, S52323D, S52323E, S52323F, S52323G, S52323H, S52323J, S52323K, S52323M, S52323N, S52323P, S52323Q, S52323R, S52323S, S52324A, S52324B, S52324C, S52324D, S52324E, S52324F, S52324G, S52324H, S52324J, S52324K, S52324M, S52324N, S52324P, S52324Q, S52324R, S52324S, S52325A, S52325B, S52325C, S52325D, S52325E, S52325F, S52325G, S52325H, S52325J, S52325K, S52325M, S52325N, S52325P, S52325Q, S52325R, S52325S, S52326A, S52326B, S52326C, S52326D, S52326E, S52326F, S52326G, S52326H, S52326J, S52326K, S52326M, S52326N, S52326P, S52326Q, S52326R, S52326S, S52331A, S52331B, S52331C, S52331D, S52331E, S52331F, S52331G, S52331H, S52331J, S52331K, S52331M, S52331N, S52331P, S52331Q, S52331R, S52331S, S52332A, S52332B, S52332C, S52332D, S52332E, S52332F, S52332G, S52332H, S52332J, S52332K, S52332M, S52332N, S52332P, S52332Q, S52332R, S52332S, S52333A, S52333B, S52333C, S52333D, S52333E, S52333F, S52333G, S52333H, S52333J, S52333K, S52333M, S52333N, S52333P, S52333Q, S52333R, S52333S, S52334A, S52334B, S52334C, S52334D, S52334E, S52334F, S52334G, S52334H, S52334J, S52334K, S52334M, S52334N, S52334P, S52334Q, S52334R, S52334S, S52335A, S52335B, S52335C, S52335D, S52335E, S52335F, S52335G, S52335H, S52335J, S52335K, S52335M, S52335N, S52335P, S52335Q, S52335R, S52335S, S52336A, S52336B, S52336C, S52336D, S52336E, S52336F, S52336G, S52336H, S52336J, S52336K, S52336M, S52336N, S52336P, S52336Q, S52336R, S52336S, S52341A, S52341B, S52341C, S52341D, S52341E, S52341F, S52341G, S52341H, S52341J, S52341K, S52341M, S52341N, S52341P, S52341Q, S52341R, S52341S, S52342A, S52342B, S52342C, S52342D, S52342E, S52342F, S52342G, S52342H, S52342J, S52342K, S52342M, S52342N, S52342P, S52342Q, S52342R, S52342S, S52343A, S52343B, S52343C, S52343D, S52343E, S52343F, S52343G, S52343H, S52343J, S52343K, S52343M, S52343N, S52343P, S52343Q, S52343R, S52343S, S52344A, S52344B, S52344C, S52344D, S52344E, S52344F, S52344G, S52344H, S52344J, S52344K, S52344M,

	S52344N, S52344P, S52344Q, S52344R, S52344S, S52345A, S52345B, S52345C, S52345D, S52345E, S52345F, S52345G, S52345H, S52345J, S52345K, S52345M, S52345N, S52345P, S52345Q, S52345R, S52345S, S52346A, S52346B, S52346C, S52346D, S52346E, S52346F, S52346G, S52346H, S52346J, S52346K, S52346M, S52346N, S52346P, S52346Q, S52346R, S52346S, S52351A, S52351B, S52351C, S52351D, S52351E, S52351F, S52351G, S52351H, S52351J, S52351K, S52351M, S52351N, S52351P, S52351Q, S52351R, S52351S, S52352A, S52352B, S52352C, S52352D, S52352E, S52352F, S52352G, S52352H, S52352J, S52352K, S52352M, S52352N, S52352P, S52352Q, S52352R, S52352S, S52353A, S52353B, S52353C, S52353D, S52353E, S52353F, S52353G, S52353H, S52353J, S52353K, S52353M, S52353N, S52353P, S52353Q, S52353R, S52353S, S52354A, S52354B, S52354C, S52354D, S52354E, S52354F, S52354G, S52354H, S52354J, S52354K, S52354M, S52354N, S52354P, S52354Q, S52354R, S52354S, S52355A, S52355B, S52355C, S52355D, S52355E, S52355F, S52355G, S52355H, S52355J, S52355K, S52355M, S52355N, S52355P, S52355Q, S52355R, S52355S, S52356A, S52356B, S52356C, S52356D, S52356E, S52356F, S52356G, S52356H, S52356J, S52356K, S52356M, S52356N, S52356P, S52356Q, S52356R, S52356S, S52361A, S52361B, S52361C, S52361D, S52361E, S52361F, S52361G, S52361H, S52361J, S52361K, S52361M, S52361N, S52361P, S52361Q, S52361R, S52361S, S52362A, S52362B, S52362C, S52362D, S52362E, S52362F, S52362G, S52362H, S52362J, S52362K, S52362M, S52362N, S52362P, S52362Q, S52362R, S52362S, S52363A, S52363B, S52363C, S52363D, S52363E, S52363F, S52363G, S52363H, S52363J, S52363K, S52363M, S52363N, S52363P, S52363Q, S52363R, S52363S, S52364A, S52364B, S52364C, S52364D, S52364E, S52364F, S52364G, S52364H, S52364J, S52364K, S52364M, S52364N, S52364P, S52364Q, S52364R, S52364S, S52365A, S52365B, S52365C, S52365D, S52365E, S52365F, S52365G, S52365H, S52365J, S52365K, S52365M, S52365N, S52365P, S52365Q, S52365R, S52365S, S52366A, S52366B, S52366C, S52366D, S52366E, S52366F, S52366G, S52366H, S52366J, S52366K, S52366M, S52366N, S52366P, S52366Q, S52366R, S52366S, S52371A, S52371B, S52371C, S52371D, S52371E, S52371F, S52371G, S52371H, S52371J, S52371K, S52371M, S52371N, S52371P, S52371Q, S52371R, S52371S, S52372A, S52372B, S52372C, S52372D, S52372E, S52372F, S52372G, S52372H, S52372J, S52372K, S52372M, S52372N, S52372P, S52372Q, S52372R, S52372S, S52379A, S52379B, S52379C, S52379D, S52379E, S52379F, S52379G, S52379H, S52379J, S52379K, S52379M, S52379N, S52379P, S52379Q, S52379R, S52379S, S52381A, S52381B, S52381C, S52381D, S52381E, S52381F, S52381G, S52381H, S52381J, S52381K, S52381M, S52381N, S52381P, S52381Q, S52381R, S52381S, S52382A, S52382B, S52382C, S52382D, S52382E, S52382F, S52382G, S52382H, S52382J, S52382K, S52382M, S52382N, S52382P, S52382Q, S52382R,

S52382S, S52389A, S52389B, S52389C, S52389D, S52389E, S52389F, S52389G, S52389H, S52389J, S52389K, S52389M, S52389N, S52389P, S52389Q, S52389R, S52389S, S52391A, S52391B, S52391C, S52391D, S52391E, S52391F, S52391G, S52391H, S52391J, S52391K, S52391M, S52391N, S52391P, S52391Q, S52391R, S52391S, S52392A, S52392B, S52392C, S52392D, S52392E, S52392F, S52392G, S52392H, S52392J, S52392K, S52392M, S52392N, S52392P, S52392Q, S52392R, S52392S, S52399A, S52399B, S52399C, S52399D, S52399E, S52399F, S52399G, S52399H, S52399J, S52399K, S52399M, S52399N, S52399P, S52399Q, S52399R, S52399S, S5290XA, S5290XB, S5290XC, S5290XD, S5290XE, S5290XF, S5290XG, S5290XH, S5290XJ, S5290XK, S5290XM, S5290XN, S5290XP, S5290XQ, S5290XR, S5290XS, S5291XA, S5291XB, S5291XC, S5291XD, S5291XE, S5291XF, S5291XG, S5291XH, S5291XJ, S5291XK, S5291XM, S5291XN, S5291XP, S5291XQ, S5291XR, S5291XS, S5292XA, S5292XB, S5292XC, S5292XD, S5292XE, S5292XF, S5292XG, S5292XH, S5292XJ, S5292XK, S5292XM, S5292XN, S5292XP, S5292XQ, S5292XR, S5292XS

Sternum, clavicle and ribs:

S2220XA, S2220XB, S2220XD, S2220XG, S2220XK, S2220XS, S2221XA, S2221XB, S2221XD, S2221XG, S2221XK, S2221XS, S2222XA, S2222XB, S2222XD, S2222XG, S2222XK, S2222XS, S2223XA, S2223XB, S2223XD, S2223XG, S2223XK, S2223XS, S2224XA, S2224XB, S2224XD, S2224XG, S2224XK, S2224XS, S2231XA, S2231XB, S2231XD, S2231XG, S2231XK, S2231XS, S2232XA, S2232XB, S2232XD, S2232XG, S2232XK, S2232XS, S2239XA, S2239XB, S2239XD, S2239XG, S2239XK, S2239XS, S2241XA, S2241XB, S2241XD, S2241XG, S2241XK, S2241XS, S2242XA, S2242XB, S2242XD, S2242XG, S2242XK, S2242XS, S2243XA, S2243XB, S2243XD, S2243XG, S2243XK, S2243XS, S2249XA, S2249XB, S2249XD, S2249XG, S2249XK, S2249XS, S42001A, S42001B, S42001D, S42001G, S42001K, S42001P, S42001S, S42002A, S42002B, S42002D, S42002G, S42002K, S42002P, S42002S, S42009A, S42009B, S42009D, S42009G, S42009K, S42009P, S42009S, S42011A, S42011B, S42011D, S42011G, S42011K, S42011P, S42011S, S42012A, S42012B, S42012D, S42012G, S42012K, S42012P, S42012S, S42013A, S42013B, S42013D, S42013G, S42013K, S42013P, S42013S, S42014A, S42014B, S42014D, S42014G, S42014K, S42014P, S42014S, S42015A, S42015B, S42015D, S42015G, S42015K, S42015P, S42015S, S42016A, S42016B, S42016D, S42016G, S42016K, S42016P, S42016S, S42017A, S42017B, S42017D, S42017G, S42017K, S42017P, S42017S, S42018A, S42018B, S42018D, S42018G, S42018K, S42018P, S42018S, S42019A, S42019B, S42019D, S42019G, S42019K, S42019P, S42019S, S42021A, S42021B, S42021D, S42021G, S42021K, S42021P, S42021S, S42022A, S42022B, S42022D, S42022G, S42022K, S42022P, S42022S, S42023A, S42023B, S42023D, S42023G, S42023K, S42023P, S42023S, S42024A, S42024B, S42024D, S42024G, S42024K,

S42024P, S42024S, S42025A, S42025B, S42025D, S42025G, S42025K, S42025P, S42025S, S42026A, S42026B, S42026D, S42026G, S42026K, S42026P, S42026S, S42031A, S42031B, S42031D, S42031G, S42031K, S42031P, S42031S, S42032A, S42032B, S42032D, S42032G, S42032K, S42032P, S42032S, S42033A, S42033B, S42033D, S42033G, S42033K, S42033P, S42033S, S42034A, S42034B, S42034D, S42034G, S42034K, S42034P, S42034S, S42035A, S42035B, S42035D, S42035G, S42035K, S42035P, S42035S, S42036A, S42036B, S42036D, S42036G, S42036K, S42036P, S42036S

Vertebrae:

S22000A, S22000B, S22000D, S22000G, S22000K, S22000S, S22001A, S22001B, S22001D, S22001G, S22001K, S22001S, S22002A, S22002B, S22002D, S22002G, S22002K, S22002S, S22008A, S22008B, S22008D, S22008G, S22008K, S22008S, S22009A, S22009B, S22009D, S22009G, S22009K, S22009S, S22010A, S22010B, S22010D, S22010G, S22010K, S22010S, S22011A, S22011B, S22011D, S22011G, S22011K, S22011S, S22012A, S22012B, S22012D, S22012G, S22012K, S22012S, S22018A, S22018B, S22018D, S22018G, S22018K, S22018S, S22019A, S22019B, S22019D, S22019G, S22019K, S22019S, S22020A, S22020B, S22020D, S22020G, S22020K, S22020S, S22021A, S22021B, S22021D, S22021G, S22021K, S22021S, S22022A, S22022B, S22022D, S22022G, S22022K, S22022S, S22028A, S22028B, S22028D, S22028G, S22028K, S22028S, S22029A, S22029B, S22029D, S22029G, S22029K, S22029S, S22030A, S22030B, S22030D, S22030G, S22030K, S22030S, S22031A, S22031B, S22031D, S22031G, S22031K, S22031S, S22032A, S22032B, S22032D, S22032G, S22032K, S22032S, S22038A, S22038B, S22038D, S22038G, S22038K, S22038S, S22039A, S22039B, S22039D, S22039G, S22039K, S22039S, S22040A, S22040B, S22040D, S22040G, S22040K, S22040S, S22041A, S22041B, S22041D, S22041G, S22041K, S22041S, S22042A, S22042B, S22042D, S22042G, S22042K, S22042S, S22048A, S22048B, S22048D, S22048G, S22048K, S22048S, S22049A, S22049B, S22049D, S22049G, S22049K, S22049S, S22050A, S22050B, S22050D, S22050G, S22050K, S22050S, S22051A, S22051B, S22051D, S22051G, S22051K, S22051S, S22052A, S22052B, S22052D, S22052G, S22052K, S22052S, S22058A, S22058B, S22058D, S22058G, S22058K, S22058S, S22059A, S22059B, S22059D, S22059G, S22059K, S22059S, S22060A, S22060B, S22060D, S22060G, S22060K, S22060S, S22061A, S22061B, S22061D, S22061G, S22061K, S22061S, S22062A, S22062B, S22062D, S22062G, S22062K, S22062S, S22068A, S22068B, S22068D, S22068G, S22068K, S22068S, S22069A, S22069B, S22069D, S22069G, S22069K, S22069S, S22070A, S22070B, S22070D, S22070G, S22070K, S22070S, S22071A, S22071B, S22071D, S22071G, S22071K, S22071S, S22072A, S22072B, S22072D, S22072G, S22072K, S22072S, S22078A, S22078B, S22078D, S22078G, S22078K, S22078S, S22079A, S22079B, S22079D, S22079G,

	S22079K, S22079S, S22080A, S22080B, S22080D, S22080G, S22080K, S22080S, S22081A, S22081B, S22081D, S22081G, S22081K, S22081S, S22082A, S22082B, S22082D, S22082G, S22082K, S22082S, S22088A, S22088B, S22088D, S22088G, S22088K, S22088S, S22089A, S22089B, S22089D, S22089G, S22089K, S22089S, S32000A, S32000B, S32000D, S32000G, S32000K, S32000S, S32001A, S32001B, S32001D, S32001G, S32001K, S32001S, S32002A, S32002B, S32002D, S32002G, S32002K, S32002S, S32008A, S32008B, S32008D, S32008G, S32008K, S32008S, S32009A, S32009B, S32009D, S32009G, S32009K, S32009S, S32010A, S32010B, S32010D, S32010G, S32010K, S32010S, S32011A, S32011B, S32011D, S32011G, S32011K, S32011S, S32012A, S32012B, S32012D, S32012G, S32012K, S32012S, S32018A, S32018B, S32018D, S32018G, S32018K, S32018S, S32019A, S32019B, S32019D, S32019G, S32019K, S32019S, S32020A, S32020B, S32020D, S32020G, S32020K, S32020S, S32021A, S32021B, S32021D, S32021G, S32021K, S32021S, S32022A, S32022B, S32022D, S32022G, S32022K, S32022S, S32028A, S32028B, S32028D, S32028G, S32028K, S32028S, S32029A, S32029B, S32029D, S32029G, S32029K, S32029S, S32030A, S32030B, S32030D, S32030G, S32030K, S32030S, S32031A, S32031B, S32031D, S32031G, S32031K, S32031S, S32032A, S32032B, S32032D, S32032G, S32032K, S32032S, S32038A, S32038B, S32038D, S32038G, S32038K, S32038S, S32039A, S32039B, S32039D, S32039G, S32039K, S32039S, S32040A, S32040B, S32040D, S32040G, S32040K, S32040S, S32041A, S32041B, S32041D, S32041G, S32041K, S32041S, S32042A, S32042B, S32042D, S32042G, S32042K, S32042S, S32048A, S32048B, S32048D, S32048G, S32048K, S32048S, S32049A, S32049B, S32049D, S32049G, S32049K, S32049S, S32050A, S32050B, S32050D, S32050G, S32050K, S32050S, S32051A, S32051B, S32051D, S32051G, S32051K, S32051S, S32052A, S32052B, S32052D, S32052G, S32052K, S32052S, S32058A, S32058B, S32058D, S32058G, S32058K, S32058S, S32059A, S32059B, S32059D, S32059G, S32059K, S32059S, M4840XA, M4840XD, M4840XG, M4840XS, M4841XA, M4841XD, M4841XG, M4841XS, M4842XA, M4842XD, M4842XG, M4842XS, M4843XA, M4843XD, M4843XG, M4843XS, M4844XA, M4844XD, M4844XG, M4844XS, M4845XA, M4845XD, M4845XG, M4845XS, M4846XA, M4846XD, M4846XG, M4846XS, M4847XA, M4847XD, M4847XG, M4847XS, M4848XA, M4848XD, M4848XG, M4848XS, M4850XA, M4850XD, M4850XG, M4850XS, M4851XA, M4851XD, M4851XG, M4851XS, M4852XA, M4852XD, M4852XG, M4852XS, M4853XA, M4853XD, M4853XG, M4853XS, M4854XA, M4854XD, M4854XG, M4854XS, M4855XA, M4855XD, M4855XG, M4855XS, M4856XA, M4856XD, M4856XG, M4856XS, M4857XA, M4857XD, M4857XG, M4857XS, M4858XA, M4858XD, M4858XG, M4858XS<br>Wrist:

	S52501A, S52501B, S52501C, S52501D, S52501E, S52501F, S52501G, S52501H, S52501J, S52501K, S52501M, S52501N, S52501P, S52501Q, S52501R, S52501S, S52502A, S52502B, S52502C, S52502D, S52502E, S52502F, S52502G, S52502H, S52502J, S52502K, S52502M, S52502N, S52502P, S52502Q, S52502R, S52502S, S52509A, S52509B, S52509C, S52509D, S52509E, S52509F, S52509G, S52509H, S52509J, S52509K, S52509M, S52509N, S52509P, S52509Q, S52509R, S52509S, S52511A, S52511B, S52511C, S52511D, S52511E, S52511F, S52511G, S52511H, S52511J, S52511K, S52511M, S52511N, S52511P, S52511Q, S52511R, S52511S, S52512A, S52512B, S52512C, S52512D, S52512E, S52512F, S52512G, S52512H, S52512J, S52512K, S52512M, S52512N, S52512P, S52512Q, S52512R, S52512S, S52513A, S52513B, S52513C, S52513D, S52513E, S52513F, S52513G, S52513H, S52513J, S52513K, S52513M, S52513N, S52513P, S52513Q, S52513R, S52513S, S52514A, S52514B, S52514C, S52514D, S52514E, S52514F, S52514G, S52514H, S52514J, S52514K, S52514M, S52514N, S52514P, S52514Q, S52514R, S52514S, S52515A, S52515B, S52515C, S52515D, S52515E, S52515F, S52515G, S52515H, S52515J, S52515K, S52515M, S52515N, S52515P, S52515Q, S52515R, S52515S, S52516A, S52516B, S52516C, S52516D, S52516E, S52516F, S52516G, S52516H, S52516J, S52516K, S52516M, S52516N, S52516P, S52516Q, S52516R, S52516S, S52521A, S52521D, S52521G, S52521K, S52521P, S52521S, S52522A, S52522D, S52522G, S52522K, S52522P, S52522S, S52529A, S52529D, S52529G, S52529K, S52529P, S52529S, S52531A, S52531B, S52531C, S52531D, S52531E, S52531F, S52531G, S52531H, S52531J, S52531K, S52531M, S52531N, S52531P, S52531Q, S52531R, S52531S, S52532A, S52532B, S52532C, S52532D, S52532E, S52532F, S52532G, S52532H, S52532J, S52532K, S52532M, S52532N, S52532P, S52532Q, S52532R, S52532S, S52539A, S52539B, S52539C, S52539D, S52539E, S52539F, S52539G, S52539H, S52539J, S52539K, S52539M, S52539N, S52539P, S52539Q, S52539R, S52539S, S52541A, S52541B, S52541C, S52541D, S52541E, S52541F, S52541G, S52541H, S52541J, S52541K, S52541M, S52541N, S52541P, S52541Q, S52541R, S52541S, S52542A, S52542B, S52542C, S52542D, S52542E, S52542F, S52542G, S52542H, S52542J, S52542K, S52542M, S52542N, S52542P, S52542Q, S52542R, S52542S, S52549A, S52549B, S52549C, S52549D, S52549E, S52549F, S52549G, S52549H, S52549J, S52549K, S52549M, S52549N, S52549P, S52549Q, S52549R, S52549S, S52551A, S52551B, S52551C, S52551D, S52551E, S52551F, S52551G, S52551H, S52551J, S52551K, S52551M, S52551N, S52551P, S52551Q, S52551R, S52551S, S52552A, S52552B, S52552C, S52552D, S52552E, S52552F, S52552G, S52552H, S52552J, S52552K, S52552M, S52552N, S52552P, S52552Q, S52552R, S52552S, S52559A, S52559B, S52559C, S52559D, S52559E, S52559F, S52559G, S52559H, S52559J, S52559K, S52559M, S52559N, S52559P, S52559Q, S52559R, S52559S, S52561A, S52561B,

	S52561C, S52561D, S52561E, S52561F, S52561G, S52561H, S52561J, S52561K, S52561M, S52561N, S52561P, S52561Q, S52561R, S52561S, S52562A, S52562B, S52562C, S52562D, S52562E, S52562F, S52562G, S52562H, S52562J, S52562K, S52562M, S52562N, S52562P, S52562Q, S52562R, S52562S, S52569A, S52569B, S52569C, S52569D, S52569E, S52569F, S52569G, S52569H, S52569J, S52569K, S52569M, S52569N, S52569P, S52569Q, S52569R, S52569S, S52571A, S52571B, S52571C, S52571D, S52571E, S52571F, S52571G, S52571H, S52571J, S52571K, S52571M, S52571N, S52571P, S52571Q, S52571R, S52571S, S52572A, S52572B, S52572C, S52572D, S52572E, S52572F, S52572G, S52572H, S52572J, S52572K, S52572M, S52572N, S52572P, S52572Q, S52572R, S52572S, S52579A, S52579B, S52579C, S52579D, S52579E, S52579F, S52579G, S52579H, S52579J, S52579K, S52579M, S52579N, S52579P, S52579Q, S52579R, S52579S, S52591A, S52591B, S52591C, S52591D, S52591E, S52591F, S52591G, S52591H, S52591J, S52591K, S52591M, S52591N, S52591P, S52591Q, S52591R, S52591S, S52592A, S52592B, S52592C, S52592D, S52592E, S52592F, S52592G, S52592H, S52592J, S52592K, S52592M, S52592N, S52592P, S52592Q, S52592R, S52592S, S52599A, S52599B, S52599C, S52599D, S52599E, S52599F, S52599G, S52599H, S52599J, S52599K, S52599M, S52599N, S52599P, S52599Q, S52599R, S52599S, S52601A, S52601B, S52601C, S52601D, S52601E, S52601F, S52601G, S52601H, S52601J, S52601K, S52601M, S52601N, S52601P, S52601Q, S52601R, S52601S, S52602A, S52602B, S52602C, S52602D, S52602E, S52602F, S52602G, S52602H, S52602J, S52602K, S52602M, S52602N, S52602P, S52602Q, S52602R, S52602S, S52609A, S52609B, S52609C, S52609D, S52609E, S52609F, S52609G, S52609H, S52609J, S52609K, S52609M, S52609N, S52609P, S52609Q, S52609R, S52609S, S52611A, S52611B, S52611C, S52611D, S52611E, S52611F, S52611G, S52611H, S52611J, S52611K, S52611M, S52611N, S52611P, S52611Q, S52611R, S52611S, S52612A, S52612B, S52612C, S52612D, S52612E, S52612F, S52612G, S52612H, S52612J, S52612K, S52612M, S52612N, S52612P, S52612Q, S52612R, S52612S, S52613A, S52613B, S52613C, S52613D, S52613E, S52613F, S52613G, S52613H, S52613J, S52613K, S52613M, S52613N, S52613P, S52613Q, S52613R, S52613S, S52614A, S52614B, S52614C, S52614D, S52614E, S52614F, S52614G, S52614H, S52614J, S52614K, S52614M, S52614N, S52614P, S52614Q, S52614R, S52614S, S52615A, S52615B, S52615C, S52615D, S52615E, S52615F, S52615G, S52615H, S52615J, S52615K, S52615M, S52615N, S52615P, S52615Q, S52615R, S52615S, S52616A, S52616B, S52616C, S52616D, S52616E, S52616F, S52616G, S52616H, S52616J, S52616K, S52616M, S52616N, S52616P, S52616Q, S52616R, S52616S, S52621A, S52621D, S52621G, S52621K, S52621P, S52621S, S52622A, S52622D, S52622G, S52622K, S52622P, S52622S, S52629A, S52629D, S52629G, S52629K, S52629P, S52629S, S52691A, S52691B, S52691C, S52691D,

	S52691E, S52691F, S52691G, S52691H, S52691J, S52691K, S52691M, S52691N, S52691P, S52691Q, S52691R, S52691S, S52692A, S52692B, S52692C, S52692D, S52692E, S52692F, S52692G, S52692H, S52692J, S52692K, S52692M, S52692N, S52692P, S52692Q, S52692R, S52692S, S52699A, S52699B, S52699C, S52699D, S52699E, S52699F, S52699G, S52699H, S52699J, S52699K, S52699M, S52699N, S52699P, S52699Q, S52699R, S52699S<br>Other (tibia, fibula, knee):<br>S82001A, S82001B, S82001C, S82001D, S82001E, S82001F, S82001G, S82001H, S82001J, S82001K, S82001M, S82001N, S82001P, S82001Q, S82001R, S82001S, S82002A, S82002B, S82002C, S82002D, S82002E, S82002F, S82002G, S82002H, S82002J, S82002K, S82002M, S82002N, S82002P, S82002Q, S82002R, S82002S, S82009A, S82009B, S82009C, S82009D, S82009E, S82009F, S82009G, S82009H, S82009J, S82009K, S82009M, S82009N, S82009P, S82009Q, S82009R, S82009S, S82011A, S82011B, S82011C, S82011D, S82011E, S82011F, S82011G, S82011H, S82011J, S82011K, S82011M, S82011N, S82011P, S82011Q, S82011R, S82011S, S82012A, S82012B, S82012C, S82012D, S82012E, S82012F, S82012G, S82012H, S82012J, S82012K, S82012M, S82012N, S82012P, S82012Q, S82012R, S82012S, S82013A, S82013B, S82013C, S82013D, S82013E, S82013F, S82013G, S82013H, S82013J, S82013K, S82013M, S82013N, S82013P, S82013Q, S82013R, S82013S, S82014A, S82014B, S82014C, S82014D, S82014E, S82014F, S82014G, S82014H, S82014J, S82014K, S82014M, S82014N, S82014P, S82014Q, S82014R, S82014S, S82015A, S82015B, S82015C, S82015D, S82015E, S82015F, S82015G, S82015H, S82015J, S82015K, S82015M, S82015N, S82015P, S82015Q, S82015R, S82015S, S82016A, S82016B, S82016C, S82016D, S82016E, S82016F, S82016G, S82016H, S82016J, S82016K, S82016M, S82016N, S82016P, S82016Q, S82016R, S82016S, S82021A, S82021B, S82021C, S82021D, S82021E, S82021F, S82021G, S82021H, S82021J, S82021K, S82021M, S82021N, S82021P, S82021Q, S82021R, S82021S, S82022A, S82022B, S82022C, S82022D, S82022E, S82022F, S82022G, S82022H, S82022J, S82022K, S82022M, S82022N, S82022P, S82022Q, S82022R, S82022S, S82023A, S82023B, S82023C, S82023D, S82023E, S82023F, S82023G, S82023H, S82023J, S82023K, S82023M, S82023N, S82023P, S82023Q, S82023R, S82023S, S82024A, S82024B, S82024C, S82024D, S82024E, S82024F, S82024G, S82024H, S82024J, S82024K, S82024M, S82024N, S82024P, S82024Q, S82024R, S82024S, S82025A, S82025B, S82025C, S82025D, S82025E, S82025F, S82025G, S82025H, S82025J, S82025K, S82025M, S82025N, S82025P, S82025Q, S82025R, S82025S, S82026A, S82026B, S82026C, S82026D, S82026E, S82026F, S82026G, S82026H, S82026J, S82026K, S82026M, S82026N, S82026P, S82026Q, S82026R, S82026S, S82031A, S82031B, S82031C, S82031D, S82031E, S82031F, S82031G, S82031H, S82031J, S82031K, S82031M, S82031N,

	S82031P, S82031Q, S82031R, S82031S, S82032A, S82032B, S82032C, S82032D, S82032E, S82032F, S82032G, S82032H, S82032J, S82032K, S82032M, S82032N, S82032P, S82032Q, S82032R, S82032S, S82033A, S82033B, S82033C, S82033D, S82033E, S82033F, S82033G, S82033H, S82033J, S82033K, S82033M, S82033N, S82033P, S82033Q, S82033R, S82033S, S82034A, S82034B, S82034C, S82034D, S82034E, S82034F, S82034G, S82034H, S82034J, S82034K, S82034M, S82034N, S82034P, S82034Q, S82034R, S82034S, S82035A, S82035B, S82035C, S82035D, S82035E, S82035F, S82035G, S82035H, S82035J, S82035K, S82035M, S82035N, S82035P, S82035Q, S82035R, S82035S, S82036A, S82036B, S82036C, S82036D, S82036E, S82036F, S82036G, S82036H, S82036J, S82036K, S82036M, S82036N, S82036P, S82036Q, S82036R, S82036S, S82041A, S82041B, S82041C, S82041D, S82041E, S82041F, S82041G, S82041H, S82041J, S82041K, S82041M, S82041N, S82041P, S82041Q, S82041R, S82041S, S82042A, S82042B, S82042C, S82042D, S82042E, S82042F, S82042G, S82042H, S82042J, S82042K, S82042M, S82042N, S82042P, S82042Q, S82042R, S82042S, S82043A, S82043B, S82043C, S82043D, S82043E, S82043F, S82043G, S82043H, S82043J, S82043K, S82043M, S82043N, S82043P, S82043Q, S82043R, S82043S, S82044A, S82044B, S82044C, S82044D, S82044E, S82044F, S82044G, S82044H, S82044J, S82044K, S82044M, S82044N, S82044P, S82044Q, S82044R, S82044S, S82045A, S82045B, S82045C, S82045D, S82045E, S82045F, S82045G, S82045H, S82045J, S82045K, S82045M, S82045N, S82045P, S82045Q, S82045R, S82045S, S82046A, S82046B, S82046C, S82046D, S82046E, S82046F, S82046G, S82046H, S82046J, S82046K, S82046M, S82046N, S82046P, S82046Q, S82046R, S82046S, S82091A, S82091B, S82091C, S82091D, S82091E, S82091F, S82091G, S82091H, S82091J, S82091K, S82091M, S82091N, S82091P, S82091Q, S82091R, S82091S, S82092A, S82092B, S82092C, S82092D, S82092E, S82092F, S82092G, S82092H, S82092J, S82092K, S82092M, S82092N, S82092P, S82092Q, S82092R, S82092S, S82099A, S82099B, S82099C, S82099D, S82099E, S82099F, S82099G, S82099H, S82099J, S82099K, S82099M, S82099N, S82099P, S82099Q, S82099R, S82099S, S82101A, S82101B, S82101C, S82101D, S82101E, S82101F, S82101G, S82101H, S82101J, S82101K, S82101M, S82101N, S82101P, S82101Q, S82101R, S82101S, S82102A, S82102B, S82102C, S82102D, S82102E, S82102F, S82102G, S82102H, S82102J, S82102K, S82102M, S82102N, S82102P, S82102Q, S82102R, S82102S, S82109A, S82109B, S82109C, S82109D, S82109E, S82109F, S82109G, S82109H, S82109J, S82109K, S82109M, S82109N, S82109P, S82109Q, S82109R, S82109S, S82111A, S82111B, S82111C, S82111D, S82111E, S82111F, S82111G, S82111H, S82111J, S82111K, S82111M, S82111N, S82111P, S82111Q, S82111R, S82111S, S82112A, S82112B, S82112C, S82112D, S82112E, S82112F, S82112G, S82112H, S82112J, S82112K, S82112M, S82112N, S82112P, S82112Q, S82112R, S82112S,

	S82113A, S82113B, S82113C, S82113D, S82113E, S82113F, S82113G, S82113H, S82113J, S82113K, S82113M, S82113N, S82113P, S82113Q, S82113R, S82113S, S82114A, S82114B, S82114C, S82114D, S82114E, S82114F, S82114G, S82114H, S82114J, S82114K, S82114M, S82114N, S82114P, S82114Q, S82114R, S82114S, S82115A, S82115B, S82115C, S82115D, S82115E, S82115F, S82115G, S82115H, S82115J, S82115K, S82115M, S82115N, S82115P, S82115Q, S82115R, S82115S, S82116A, S82116B, S82116C, S82116D, S82116E, S82116F, S82116G, S82116H, S82116J, S82116K, S82116M, S82116N, S82116P, S82116Q, S82116R, S82116S, S82121A, S82121B, S82121C, S82121D, S82121E, S82121F, S82121G, S82121H, S82121J, S82121K, S82121M, S82121N, S82121P, S82121Q, S82121R, S82121S, S82122A, S82122B, S82122C, S82122D, S82122E, S82122F, S82122G, S82122H, S82122J, S82122K, S82122M, S82122N, S82122P, S82122Q, S82122R, S82122S, S82123A, S82123B, S82123C, S82123D, S82123E, S82123F, S82123G, S82123H, S82123J, S82123K, S82123M, S82123N, S82123P, S82123Q, S82123R, S82123S, S82124A, S82124B, S82124C, S82124D, S82124E, S82124F, S82124G, S82124H, S82124J, S82124K, S82124M, S82124N, S82124P, S82124Q, S82124R, S82124S, S82125A, S82125B, S82125C, S82125D, S82125E, S82125F, S82125G, S82125H, S82125J, S82125K, S82125M, S82125N, S82125P, S82125Q, S82125R, S82125S, S82126A, S82126B, S82126C, S82126D, S82126E, S82126F, S82126G, S82126H, S82126J, S82126K, S82126M, S82126N, S82126P, S82126Q, S82126R, S82126S, S82131A, S82131B, S82131C, S82131D, S82131E, S82131F, S82131G, S82131H, S82131J, S82131K, S82131M, S82131N, S82131P, S82131Q, S82131R, S82131S, S82132A, S82132B, S82132C, S82132D, S82132E, S82132F, S82132G, S82132H, S82132J, S82132K, S82132M, S82132N, S82132P, S82132Q, S82132R, S82132S, S82133A, S82133B, S82133C, S82133D, S82133E, S82133F, S82133G, S82133H, S82133J, S82133K, S82133M, S82133N, S82133P, S82133Q, S82133R, S82133S, S82134A, S82134B, S82134C, S82134D, S82134E, S82134F, S82134G, S82134H, S82134J, S82134K, S82134M, S82134N, S82134P, S82134Q, S82134R, S82134S, S82135A, S82135B, S82135C, S82135D, S82135E, S82135F, S82135G, S82135H, S82135J, S82135K, S82135M, S82135N, S82135P, S82135Q, S82135R, S82135S, S82136A, S82136B, S82136C, S82136D, S82136E, S82136F, S82136G, S82136H, S82136J, S82136K, S82136M, S82136N, S82136P, S82136Q, S82136R, S82136S, S82141A, S82141B, S82141C, S82141D, S82141E, S82141F, S82141G, S82141H, S82141J, S82141K, S82141M, S82141N, S82141P, S82141Q, S82141R, S82141S, S82142A, S82142B, S82142C, S82142D, S82142E, S82142F, S82142G, S82142H, S82142J, S82142K, S82142M, S82142N, S82142P, S82142Q, S82142R, S82142S, S82143A, S82143B, S82143C, S82143D, S82143E, S82143F, S82143G, S82143H, S82143J, S82143K, S82143M, S82143N, S82143P, S82143Q, S82143R, S82143S, S82144A, S82144B, S82144C, S82144D,

	S82144E, S82144F, S82144G, S82144H, S82144J, S82144K, S82144M, S82144N, S82144P, S82144Q, S82144R, S82144S, S82145A, S82145B, S82145C, S82145D, S82145E, S82145F, S82145G, S82145H, S82145J, S82145K, S82145M, S82145N, S82145P, S82145Q, S82145R, S82145S, S82146A, S82146B, S82146C, S82146D, S82146E, S82146F, S82146G, S82146H, S82146J, S82146K, S82146M, S82146N, S82146P, S82146Q, S82146R, S82146S, S82151A, S82151B, S82151C, S82151D, S82151E, S82151F, S82151G, S82151H, S82151J, S82151K, S82151M, S82151N, S82151P, S82151Q, S82151R, S82151S, S82152A, S82152B, S82152C, S82152D, S82152E, S82152F, S82152G, S82152H, S82152J, S82152K, S82152M, S82152N, S82152P, S82152Q, S82152R, S82152S, S82153A, S82153B, S82153C, S82153D, S82153E, S82153F, S82153G, S82153H, S82153J, S82153K, S82153M, S82153N, S82153P, S82153Q, S82153R, S82153S, S82154A, S82154B, S82154C, S82154D, S82154E, S82154F, S82154G, S82154H, S82154J, S82154K, S82154M, S82154N, S82154P, S82154Q, S82154R, S82154S, S82155A, S82155B, S82155C, S82155D, S82155E, S82155F, S82155G, S82155H, S82155J, S82155K, S82155M, S82155N, S82155P, S82155Q, S82155R, S82155S, S82156A, S82156B, S82156C, S82156D, S82156E, S82156F, S82156G, S82156H, S82156J, S82156K, S82156M, S82156N, S82156P, S82156Q, S82156R, S82156S, S82161A, S82161D, S82161G, S82161K, S82161P, S82161S, S82162A, S82162D, S82162G, S82162K, S82162P, S82162S, S82169A, S82169D, S82169G, S82169K, S82169P, S82169S, S82191A, S82191B, S82191C, S82191D, S82191E, S82191F, S82191G, S82191H, S82191J, S82191K, S82191M, S82191N, S82191P, S82191Q, S82191R, S82191S, S82192A, S82192B, S82192C, S82192D, S82192E, S82192F, S82192G, S82192H, S82192J, S82192K, S82192M, S82192N, S82192P, S82192Q, S82192R, S82192S, S82199A, S82199B, S82199C, S82199D, S82199E, S82199F, S82199G, S82199H, S82199J, S82199K, S82199M, S82199N, S82199P, S82199Q, S82199R, S82199S, S82201A, S82201B, S82201C, S82201D, S82201E, S82201F, S82201G, S82201H, S82201J, S82201K, S82201M, S82201N, S82201P, S82201Q, S82201R, S82201S, S82202A, S82202B, S82202C, S82202D, S82202E, S82202F, S82202G, S82202H, S82202J, S82202K, S82202M, S82202N, S82202P, S82202Q, S82202R, S82202S, S82209A, S82209B, S82209C, S82209D, S82209E, S82209F, S82209G, S82209H, S82209J, S82209K, S82209M, S82209N, S82209P, S82209Q, S82209R, S82209S, S82221A, S82221B, S82221C, S82221D, S82221E, S82221F, S82221G, S82221H, S82221J, S82221K, S82221M, S82221N, S82221P, S82221Q, S82221R, S82221S, S82222A, S82222B, S82222C, S82222D, S82222E, S82222F, S82222G, S82222H, S82222J, S82222K, S82222M, S82222N, S82222P, S82222Q, S82222R, S82222S, S82223A, S82223B, S82223C, S82223D, S82223E, S82223F, S82223G, S82223H, S82223J, S82223K, S82223M, S82223N, S82223P, S82223Q, S82223R, S82223S, S82224A, S82224B, S82224C, S82224D, S82224E, S82224F,

S82224G, S82224H, S82224J, S82224K, S82224M, S82224N, S82224P, S82224Q, S82224R, S82224S, S82225A, S82225B, S82225C, S82225D, S82225E, S82225F, S82225G, S82225H, S82225J, S82225K, S82225M, S82225N, S82225P, S82225Q, S82225R, S82225S, S82226A, S82226B, S82226C, S82226D, S82226E, S82226F, S82226G, S82226H, S82226J, S82226K, S82226M, S82226N, S82226P, S82226Q, S82226R, S82226S, S82231A, S82231B, S82231C, S82231D, S82231E, S82231F, S82231G, S82231H, S82231J, S82231K, S82231M, S82231N, S82231P, S82231Q, S82231R, S82231S, S82232A, S82232B, S82232C, S82232D, S82232E, S82232F, S82232G, S82232H, S82232J, S82232K, S82232M, S82232N, S82232P, S82232Q, S82232R, S82232S, S82233A, S82233B, S82233C, S82233D, S82233E, S82233F, S82233G, S82233H, S82233J, S82233K, S82233M, S82233N, S82233P, S82233Q, S82233R, S82233S, S82234A, S82234B, S82234C, S82234D, S82234E, S82234F, S82234G, S82234H, S82234J, S82234K, S82234M, S82234N, S82234P, S82234Q, S82234R, S82234S, S82235A, S82235B, S82235C, S82235D, S82235E, S82235F, S82235G, S82235H, S82235J, S82235K, S82235M, S82235N, S82235P, S82235Q, S82235R, S82235S, S82236A, S82236B, S82236C, S82236D, S82236E, S82236F, S82236G, S82236H, S82236J, S82236K, S82236M, S82236N, S82236P, S82236Q, S82236R, S82236S, S82241A, S82241B, S82241C, S82241D, S82241E, S82241F, S82241G, S82241H, S82241J, S82241K, S82241M, S82241N, S82241P, S82241Q, S82241R, S82241S, S82242A, S82242B, S82242C, S82242D, S82242E, S82242F, S82242G, S82242H, S82242J, S82242K, S82242M, S82242N, S82242P, S82242Q, S82242R, S82242S, S82243A, S82243B, S82243C, S82243D, S82243E, S82243F, S82243G, S82243H, S82243J, S82243K, S82243M, S82243N, S82243P, S82243Q, S82243R, S82243S, S82244A, S82244B, S82244C, S82244D, S82244E, S82244F, S82244G, S82244H, S82244J, S82244K, S82244M, S82244N, S82244P, S82244Q, S82244R, S82244S, S82245A, S82245B, S82245C, S82245D, S82245E, S82245F, S82245G, S82245H, S82245J, S82245K, S82245M, S82245N, S82245P, S82245Q, S82245R, S82245S, S82246A, S82246B, S82246C, S82246D, S82246E, S82246F, S82246G, S82246H, S82246J, S82246K, S82246M, S82246N, S82246P, S82246Q, S82246R, S82246S, S82251A, S82251B, S82251C, S82251D, S82251E, S82251F, S82251G, S82251H, S82251J, S82251K, S82251M, S82251N, S82251P, S82251Q, S82251R, S82251S, S82252A, S82252B, S82252C, S82252D, S82252E, S82252F, S82252G, S82252H, S82252J, S82252K, S82252M, S82252N, S82252P, S82252Q, S82252R, S82252S, S82253A, S82253B, S82253C, S82253D, S82253E, S82253F, S82253G, S82253H, S82253J, S82253K, S82253M, S82253N, S82253P, S82253Q, S82253R, S82253S, S82254A, S82254B, S82254C, S82254D, S82254E, S82254F, S82254G, S82254H, S82254J, S82254K, S82254M, S82254N, S82254P, S82254Q, S82254R, S82254S, S82255A, S82255B, S82255C, S82255D, S82255E, S82255F, S82255G, S82255H, S82255J, S82255K,

	S82255M, S82255N, S82255P, S82255Q, S82255R, S82255S, S82256A, S82256B, S82256C, S82256D, S82256E, S82256F, S82256G, S82256H, S82256J, S82256K, S82256M, S82256N, S82256P, S82256Q, S82256R, S82256S, S82261A, S82261B, S82261C, S82261D, S82261E, S82261F, S82261G, S82261H, S82261J, S82261K, S82261M, S82261N, S82261P, S82261Q, S82261R, S82261S, S82262A, S82262B, S82262C, S82262D, S82262E, S82262F, S82262G, S82262H, S82262J, S82262K, S82262M, S82262N, S82262P, S82262Q, S82262R, S82262S, S82263A, S82263B, S82263C, S82263D, S82263E, S82263F, S82263G, S82263H, S82263J, S82263K, S82263M, S82263N, S82263P, S82263Q, S82263R, S82263S, S82264A, S82264B, S82264C, S82264D, S82264E, S82264F, S82264G, S82264H, S82264J, S82264K, S82264M, S82264N, S82264P, S82264Q, S82264R, S82264S, S82265A, S82265B, S82265C, S82265D, S82265E, S82265F, S82265G, S82265H, S82265J, S82265K, S82265M, S82265N, S82265P, S82265Q, S82265R, S82265S, S82266A, S82266B, S82266C, S82266D, S82266E, S82266F, S82266G, S82266H, S82266J, S82266K, S82266M, S82266N, S82266P, S82266Q, S82266R, S82266S, S82291A, S82291B, S82291C, S82291D, S82291E, S82291F, S82291G, S82291H, S82291J, S82291K, S82291M, S82291N, S82291P, S82291Q, S82291R, S82291S, S82292A, S82292B, S82292C, S82292D, S82292E, S82292F, S82292G, S82292H, S82292J, S82292K, S82292M, S82292N, S82292P, S82292Q, S82292R, S82292S, S82299A, S82299B, S82299C, S82299D, S82299E, S82299F, S82299G, S82299H, S82299J, S82299K, S82299M, S82299N, S82299P, S82299Q, S82299R, S82299S, S82301A, S82301B, S82301C, S82301D, S82301E, S82301F, S82301G, S82301H, S82301J, S82301K, S82301M, S82301N, S82301P, S82301Q, S82301R, S82301S, S82302A, S82302B, S82302C, S82302D, S82302E, S82302F, S82302G, S82302H, S82302J, S82302K, S82302M, S82302N, S82302P, S82302Q, S82302R, S82302S, S82309A, S82309B, S82309C, S82309D, S82309E, S82309F, S82309G, S82309H, S82309J, S82309K, S82309M, S82309N, S82309P, S82309Q, S82309R, S82309S, S82311A, S82311D, S82311G, S82311K, S82311P, S82311S, S82312A, S82312D, S82312G, S82312K, S82312P, S82312S, S82319A, S82319D, S82319G, S82319K, S82319P, S82319S, S82391A, S82391B, S82391C, S82391D, S82391E, S82391F, S82391G, S82391H, S82391J, S82391K, S82391M, S82391N, S82391P, S82391Q, S82391R, S82391S, S82392A, S82392B, S82392C, S82392D, S82392E, S82392F, S82392G, S82392H, S82392J, S82392K, S82392M, S82392N, S82392P, S82392Q, S82392R, S82392S, S82399A, S82399B, S82399C, S82399D, S82399E, S82399F, S82399G, S82399H, S82399J, S82399K, S82399M, S82399N, S82399P, S82399Q, S82399R, S82399S, S82401A, S82401B, S82401C, S82401D, S82401E, S82401F, S82401G, S82401H, S82401J, S82401K, S82401M, S82401N, S82401P, S82401Q, S82401R, S82401S, S82402A, S82402B, S82402C, S82402D, S82402E, S82402F, S82402G, S82402H, S82402J, S82402K, S82402M, S82402N,

	S82402P, S82402Q, S82402R, S82402S, S82409A, S82409B, S82409C, S82409D, S82409E, S82409F, S82409G, S82409H, S82409J, S82409K, S82409M, S82409N, S82409P, S82409Q, S82409R, S82409S, S82421A, S82421B, S82421C, S82421D, S82421E, S82421F, S82421G, S82421H, S82421J, S82421K, S82421M, S82421N, S82421P, S82421Q, S82421R, S82421S, S82422A, S82422B, S82422C, S82422D, S82422E, S82422F, S82422G, S82422H, S82422J, S82422K, S82422M, S82422N, S82422P, S82422Q, S82422R, S82422S, S82423A, S82423B, S82423C, S82423D, S82423E, S82423F, S82423G, S82423H, S82423J, S82423K, S82423M, S82423N, S82423P, S82423Q, S82423R, S82423S, S82424A, S82424B, S82424C, S82424D, S82424E, S82424F, S82424G, S82424H, S82424J, S82424K, S82424M, S82424N, S82424P, S82424Q, S82424R, S82424S, S82425A, S82425B, S82425C, S82425D, S82425E, S82425F, S82425G, S82425H, S82425J, S82425K, S82425M, S82425N, S82425P, S82425Q, S82425R, S82425S, S82426A, S82426B, S82426C, S82426D, S82426E, S82426F, S82426G, S82426H, S82426J, S82426K, S82426M, S82426N, S82426P, S82426Q, S82426R, S82426S, S82431A, S82431B, S82431C, S82431D, S82431E, S82431F, S82431G, S82431H, S82431J, S82431K, S82431M, S82431N, S82431P, S82431Q, S82431R, S82431S, S82432A, S82432B, S82432C, S82432D, S82432E, S82432F, S82432G, S82432H, S82432J, S82432K, S82432M, S82432N, S82432P, S82432Q, S82432R, S82432S, S82433A, S82433B, S82433C, S82433D, S82433E, S82433F, S82433G, S82433H, S82433J, S82433K, S82433M, S82433N, S82433P, S82433Q, S82433R, S82433S, S82434A, S82434B, S82434C, S82434D, S82434E, S82434F, S82434G, S82434H, S82434J, S82434K, S82434M, S82434N, S82434P, S82434Q, S82434R, S82434S, S82435A, S82435B, S82435C, S82435D, S82435E, S82435F, S82435G, S82435H, S82435J, S82435K, S82435M, S82435N, S82435P, S82435Q, S82435R, S82435S, S82436A, S82436B, S82436C, S82436D, S82436E, S82436F, S82436G, S82436H, S82436J, S82436K, S82436M, S82436N, S82436P, S82436Q, S82436R, S82436S, S82441A, S82441B, S82441C, S82441D, S82441E, S82441F, S82441G, S82441H, S82441J, S82441K, S82441M, S82441N, S82441P, S82441Q, S82441R, S82441S, S82442A, S82442B, S82442C, S82442D, S82442E, S82442F, S82442G, S82442H, S82442J, S82442K, S82442M, S82442N, S82442P, S82442Q, S82442R, S82442S, S82443A, S82443B, S82443C, S82443D, S82443E, S82443F, S82443G, S82443H, S82443J, S82443K, S82443M, S82443N, S82443P, S82443Q, S82443R, S82443S, S82444A, S82444B, S82444C, S82444D, S82444E, S82444F, S82444G, S82444H, S82444J, S82444K, S82444M, S82444N, S82444P, S82444Q, S82444R, S82444S, S82445A, S82445B, S82445C, S82445D, S82445E, S82445F, S82445G, S82445H, S82445J, S82445K, S82445M, S82445N, S82445P, S82445Q, S82445R, S82445S, S82446A, S82446B, S82446C, S82446D, S82446E, S82446F, S82446G, S82446H, S82446J, S82446K, S82446M, S82446N, S82446P, S82446Q, S82446R, S82446S,

	S82451A, S82451B, S82451C, S82451D, S82451E, S82451F, S82451G, S82451H, S82451J, S82451K, S82451M, S82451N, S82451P, S82451Q, S82451R, S82451S, S82452A, S82452B, S82452C, S82452D, S82452E, S82452F, S82452G, S82452H, S82452J, S82452K, S82452M, S82452N, S82452P, S82452Q, S82452R, S82452S, S82453A, S82453B, S82453C, S82453D, S82453E, S82453F, S82453G, S82453H, S82453J, S82453K, S82453M, S82453N, S82453P, S82453Q, S82453R, S82453S, S82454A, S82454B, S82454C, S82454D, S82454E, S82454F, S82454G, S82454H, S82454J, S82454K, S82454M, S82454N, S82454P, S82454Q, S82454R, S82454S, S82455A, S82455B, S82455C, S82455D, S82455E, S82455F, S82455G, S82455H, S82455J, S82455K, S82455M, S82455N, S82455P, S82455Q, S82455R, S82455S, S82456A, S82456B, S82456C, S82456D, S82456E, S82456F, S82456G, S82456H, S82456J, S82456K, S82456M, S82456N, S82456P, S82456Q, S82456R, S82456S, S82461A, S82461B, S82461C, S82461D, S82461E, S82461F, S82461G, S82461H, S82461J, S82461K, S82461M, S82461N, S82461P, S82461Q, S82461R, S82461S, S82462A, S82462B, S82462C, S82462D, S82462E, S82462F, S82462G, S82462H, S82462J, S82462K, S82462M, S82462N, S82462P, S82462Q, S82462R, S82462S, S82463A, S82463B, S82463C, S82463D, S82463E, S82463F, S82463G, S82463H, S82463J, S82463K, S82463M, S82463N, S82463P, S82463Q, S82463R, S82463S, S82464A, S82464B, S82464C, S82464D, S82464E, S82464F, S82464G, S82464H, S82464J, S82464K, S82464M, S82464N, S82464P, S82464Q, S82464R, S82464S, S82465A, S82465B, S82465C, S82465D, S82465E, S82465F, S82465G, S82465H, S82465J, S82465K, S82465M, S82465N, S82465P, S82465Q, S82465R, S82465S, S82466A, S82466B, S82466C, S82466D, S82466E, S82466F, S82466G, S82466H, S82466J, S82466K, S82466M, S82466N, S82466P, S82466Q, S82466R, S82466S, S82491A, S82491B, S82491C, S82491D, S82491E, S82491F, S82491G, S82491H, S82491J, S82491K, S82491M, S82491N, S82491P, S82491Q, S82491R, S82491S, S82492A, S82492B, S82492C, S82492D, S82492E, S82492F, S82492G, S82492H, S82492J, S82492K, S82492M, S82492N, S82492P, S82492Q, S82492R, S82492S, S82499A, S82499B, S82499C, S82499D, S82499E, S82499F, S82499G, S82499H, S82499J, S82499K, S82499M, S82499N, S82499P, S82499Q, S82499R, S82499S, S8251XA, S8251XB, S8251XC, S8251XD, S8251XE, S8251XF, S8251XG, S8251XH, S8251XJ, S8251XK, S8251XM, S8251XN, S8251XP, S8251XQ, S8251XR, S8251XS, S8252XA, S8252XB, S8252XC, S8252XD, S8252XE, S8252XF, S8252XG, S8252XH, S8252XJ, S8252XK, S8252XM, S8252XN, S8252XP, S8252XQ, S8252XR, S8252XS, S8253XA, S8253XB, S8253XC, S8253XD, S8253XE, S8253XF, S8253XG, S8253XH, S8253XJ, S8253XK, S8253XM, S8253XN, S8253XP, S8253XQ, S8253XR, S8253XS, S8254XA, S8254XB, S8254XC, S8254XD, S8254XE, S8254XF, S8254XG, S8254XH, S8254XJ, S8254XK, S8254XM, S8254XN, S8254XP, S8254XQ, S8254XR, S8254XS, S8255XA, S8255XB, S8255XC, S8255XD,

	S8255XE, S8255XF, S8255XG, S8255XH, S8255XJ, S8255XK, S8255XM, S8255XN, S8255XP, S8255XQ, S8255XR, S8255XS, S8256XA, S8256XB, S8256XC, S8256XD, S8256XE, S8256XF, S8256XG, S8256XH, S8256XJ, S8256XK, S8256XM, S8256XN, S8256XP, S8256XQ, S8256XR, S8256XS, S8261XA, S8261XB, S8261XC, S8261XD, S8261XE, S8261XF, S8261XG, S8261XH, S8261XJ, S8261XK, S8261XM, S8261XN, S8261XP, S8261XQ, S8261XR, S8261XS, S8262XA, S8262XB, S8262XC, S8262XD, S8262XE, S8262XF, S8262XG, S8262XH, S8262XJ, S8262XK, S8262XM, S8262XN, S8262XP, S8262XQ, S8262XR, S8262XS, S8263XA, S8263XB, S8263XC, S8263XD, S8263XE, S8263XF, S8263XG, S8263XH, S8263XJ, S8263XK, S8263XM, S8263XN, S8263XP, S8263XQ, S8263XR, S8263XS, S8264XA, S8264XB, S8264XC, S8264XD, S8264XE, S8264XF, S8264XG, S8264XH, S8264XJ, S8264XK, S8264XM, S8264XN, S8264XP, S8264XQ, S8264XR, S8264XS, S8265XA, S8265XB, S8265XC, S8265XD, S8265XE, S8265XF, S8265XG, S8265XH, S8265XJ, S8265XK, S8265XM, S8265XN, S8265XP, S8265XQ, S8265XR, S8265XS, S8266XA, S8266XB, S8266XC, S8266XD, S8266XE, S8266XF, S8266XG, S8266XH, S8266XJ, S8266XK, S8266XM, S8266XN, S8266XP, S8266XQ, S8266XR, S8266XS, S82811A, S82811D, S82811G, S82811K, S82811P, S82811S, S82812A, S82812D, S82812G, S82812K, S82812P, S82812S, S82819A, S82819D, S82819G, S82819K, S82819P, S82819S, S82821A, S82821D, S82821G, S82821K, S82821P, S82821S, S82822A, S82822D, S82822G, S82822K, S82822P, S82822S, S82829A, S82829D, S82829G, S82829K, S82829P, S82829S, S82831A, S82831B, S82831C, S82831D, S82831E, S82831F, S82831G, S82831H, S82831J, S82831K, S82831M, S82831N, S82831P, S82831Q, S82831R, S82831S, S82832A, S82832B, S82832C, S82832D, S82832E, S82832F, S82832G, S82832H, S82832J, S82832K, S82832M, S82832N, S82832P, S82832Q, S82832R, S82832S, S82839A, S82839B, S82839C, S82839D, S82839E, S82839F, S82839G, S82839H, S82839J, S82839K, S82839M, S82839N, S82839P, S82839Q, S82839R, S82839S, S82841A, S82841B, S82841C, S82841D, S82841E, S82841F, S82841G, S82841H, S82841J, S82841K, S82841M, S82841N, S82841P, S82841Q, S82841R, S82841S, S82842A, S82842B, S82842C, S82842D, S82842E, S82842F, S82842G, S82842H, S82842J, S82842K, S82842M, S82842N, S82842P, S82842Q, S82842R, S82842S, S82843A, S82843B, S82843C, S82843D, S82843E, S82843F, S82843G, S82843H, S82843J, S82843K, S82843M, S82843N, S82843P, S82843Q, S82843R, S82843S, S82844A, S82844B, S82844C, S82844D, S82844E, S82844F, S82844G, S82844H, S82844J, S82844K, S82844M, S82844N, S82844P, S82844Q, S82844R, S82844S, S82845A, S82845B, S82845C, S82845D, S82845E, S82845F, S82845G, S82845H, S82845J, S82845K, S82845M, S82845N, S82845P, S82845Q, S82845R, S82845S, S82846A, S82846B, S82846C, S82846D, S82846E, S82846F, S82846G, S82846H, S82846J, S82846K, S82846M, S82846N, S82846P, S82846Q, S82846R, S82846S, S82851A, S82851B, S82851C, S82851D,

	S82851E, S82851F, S82851G, S82851H, S82851J, S82851K, S82851M, S82851N, S82851P, S82851Q, S82851R, S82851S, S82852A, S82852B, S82852C, S82852D, S82852E, S82852F, S82852G, S82852H, S82852J, S82852K, S82852M, S82852N, S82852P, S82852Q, S82852R, S82852S, S82853A, S82853B, S82853C, S82853D, S82853E, S82853F, S82853G, S82853H, S82853J, S82853K, S82853M, S82853N, S82853P, S82853Q, S82853R, S82853S, S82854A, S82854B, S82854C, S82854D, S82854E, S82854F, S82854G, S82854H, S82854J, S82854K, S82854M, S82854N, S82854P, S82854Q, S82854R, S82854S, S82855A, S82855B, S82855C, S82855D, S82855E, S82855F, S82855G, S82855H, S82855J, S82855K, S82855M, S82855N, S82855P, S82855Q, S82855R, S82855S, S82856A, S82856B, S82856C, S82856D, S82856E, S82856F, S82856G, S82856H, S82856J, S82856K, S82856M, S82856N, S82856P, S82856Q, S82856R, S82856S, S82861A, S82861B, S82861C, S82861D, S82861E, S82861F, S82861G, S82861H, S82861J, S82861K, S82861M, S82861N, S82861P, S82861Q, S82861R, S82861S, S82862A, S82862B, S82862C, S82862D, S82862E, S82862F, S82862G, S82862H, S82862J, S82862K, S82862M, S82862N, S82862P, S82862Q, S82862R, S82862S, S82863A, S82863B, S82863C, S82863D, S82863E, S82863F, S82863G, S82863H, S82863J, S82863K, S82863M, S82863N, S82863P, S82863Q, S82863R, S82863S, S82864A, S82864B, S82864C, S82864D, S82864E, S82864F, S82864G, S82864H, S82864J, S82864K, S82864M, S82864N, S82864P, S82864Q, S82864R, S82864S, S82865A, S82865B, S82865C, S82865D, S82865E, S82865F, S82865G, S82865H, S82865J, S82865K, S82865M, S82865N, S82865P, S82865Q, S82865R, S82865S, S82866A, S82866B, S82866C, S82866D, S82866E, S82866F, S82866G, S82866H, S82866J, S82866K, S82866M, S82866N, S82866P, S82866Q, S82866R, S82866S, S82871A, S82871B, S82871C, S82871D, S82871E, S82871F, S82871G, S82871H, S82871J, S82871K, S82871M, S82871N, S82871P, S82871Q, S82871R, S82871S, S82872A, S82872B, S82872C, S82872D, S82872E, S82872F, S82872G, S82872H, S82872J, S82872K, S82872M, S82872N, S82872P, S82872Q, S82872R, S82872S, S82873A, S82873B, S82873C, S82873D, S82873E, S82873F, S82873G, S82873H, S82873J, S82873K, S82873M, S82873N, S82873P, S82873Q, S82873R, S82873S, S82874A, S82874B, S82874C, S82874D, S82874E, S82874F, S82874G, S82874H, S82874J, S82874K, S82874M, S82874N, S82874P, S82874Q, S82874R, S82874S, S82875A, S82875B, S82875C, S82875D, S82875E, S82875F, S82875G, S82875H, S82875J, S82875K, S82875M, S82875N, S82875P, S82875Q, S82875R, S82875S, S82876A, S82876B, S82876C, S82876D, S82876E, S82876F, S82876G, S82876H, S82876J, S82876K, S82876M, S82876N, S82876P, S82876Q, S82876R, S82876S, S82891A, S82891B, S82891C, S82891D, S82891E, S82891F, S82891G, S82891H, S82891J, S82891K, S82891M, S82891N, S82891P, S82891Q, S82891R, S82891S, S82892A, S82892B, S82892C, S82892D, S82892E, S82892F, S82892G, S82892H,

S82892J, S82892K, S82892M, S82892N, S82892P, S82892Q, S82892R, S82892S, S82899A, S82899B, S82899C, S82899D, S82899E, S82899F, S82899G, S82899H, S82899J, S82899K, S82899M, S82899N, S82899P, S82899Q, S82899R, S82899S, S8290XA, S8290XB, S8290XC, S8290XD, S8290XE, S8290XF, S8290XG, S8290XH, S8290XJ, S8290XK, S8290XM, S8290XN, S8290XP, S8290XQ, S8290XR, S8290XS, S8291XA, S8291XB, S8291XC, S8291XD, S8291XE, S8291XF, S8291XG, S8291XH, S8291XJ, S8291XK, S8291XM, S8291XN, S8291XP, S8291XQ, S8291XR, S8291XS, S8292XA, S8292XB, S8292XC, S8292XD, S8292XE, S8292XF, S8292XG, S8292XH, S8292XJ, S8292XK, S8292XM, S8292XN, S8292XP, S8292XQ, S8292XR, S8292XS

Osteoporosis with fracture:

M8000XA, M8000XD, M8000XG, M8000XK, M8000XP, M8000XS, M80011A, M80011D, M80011G, M80011K, M80011P, M80011S, M80012A, M80012D, M80012G, M80012K, M80012P, M80012S, M80019A, M80019D, M80019G, M80019K, M80019P, M80019S, M80021A, M80021D, M80021G, M80021K, M80021P, M80021S, M80022A, M80022D, M80022G, M80022K, M80022P, M80022S, M80029A, M80029D, M80029G, M80029K, M80029P, M80029S, M80031A, M80031D, M80031G, M80031K, M80031P, M80031S, M80032A, M80032D, M80032G, M80032K, M80032P, M80032S, M80039A, M80039D, M80039G, M80039K, M80039P, M80039S, M80041A, M80041D, M80041G, M80041K, M80041P, M80041S, M80042A, M80042D, M80042G, M80042K, M80042P, M80042S, M80049A, M80049D, M80049G, M80049K, M80049P, M80049S, M80051A, M80051D, M80051G, M80051K, M80051P, M80051S, M80052A, M80052D, M80052G, M80052K, M80052P, M80052S, M80059A, M80059D, M80059G, M80059K, M80059P, M80059S, M80061A, M80061D, M80061G, M80061K, M80061P, M80061S, M80062A, M80062D, M80062G, M80062K, M80062P, M80062S, M80069A, M80069D, M80069G, M80069K, M80069P, M80069S, M80071A, M80071D, M80071G, M80071K, M80071P, M80071S, M80072A, M80072D, M80072G, M80072K, M80072P, M80072S, M80079A, M80079D, M80079G, M80079K, M80079P, M80079S, M8008XA, M8008XD, M8008XG, M8008XK, M8008XP, M8008XS, M800AXA, M800AXD, M800AXG, M800AXK, M800AXP, M800AXS, M8080XA, M8080XD, M8080XG, M8080XK, M8080XP, M8080XS, M80811A, M80811D, M80811G, M80811K, M80811P, M80811S, M80812A, M80812D, M80812G, M80812K, M80812P, M80812S, M80819A, M80819D, M80819G, M80819K, M80819P, M80819S, M80821A, M80821D, M80821G, M80821K, M80821P, M80821S, M80822A, M80822D, M80822G, M80822K, M80822P, M80822S, M80829A, M80829D, M80829G, M80829K, M80829P, M80829S, M80831A, M80831D, M80831G, M80831K, M80831P, M80831S, M80832A, M80832D,

	M80832G, M80832K, M80832P, M80832S, M80839A, M80839D, M80839G, M80839K, M80839P, M80839S, M80841A, M80841D, M80841G, M80841K, M80841P, M80841S, M80842A, M80842D, M80842G, M80842K, M80842P, M80842S, M80849A, M80849D, M80849G, M80849K, M80849P, M80849S, M80851A, M80851D, M80851G, M80851K, M80851P, M80851S, M80852A, M80852D, M80852G, M80852K, M80852P, M80852S, M80859A, M80859D, M80859G, M80859K, M80859P, M80859S, M80861A, M80861D, M80861G, M80861K, M80861P, M80861S, M80862A, M80862D, M80862G, M80862K, M80862P, M80862S, M80869A, M80869D, M80869G, M80869K, M80869P, M80869S, M80871A, M80871D, M80871G, M80871K, M80871P, M80871S, M80872A, M80872D, M80872G, M80872K, M80872P, M80872S, M80879A, M80879D, M80879G, M80879K, M80879P, M80879S, M8088XA, M8088XD, M8088XG, M8088XK, M8088XP, M8088XS, M808AXA, M808AXD, M808AXG, M808AXK, M808AXP, M808AXS
**Fragility fracture (Exclusion)**	Accident:<br>V0001XA, V0001XD, V0001XS, V0002XA, V0002XD, V0002XS, V00031A, V00031D, V00031S, V00038A, V00038D, V00038S, V0009XA, V0009XD, V0009XS, V00111A, V00111D, V00111S, V00112A, V00112D, V00112S, V00118A, V00118D, V00118S, V00121A, V00121D, V00121S, V00122A, V00122D, V00122S, V00128A, V00128D, V00128S, V00131A, V00131D, V00131S, V00132A, V00132D, V00132S, V00138A, V00138D, V00138S, V00141A, V00141D, V00141S, V00142A, V00142D, V00142S, V00148A, V00148D, V00148S, V00151A, V00151D, V00151S, V00152A, V00152D, V00152S, V00158A, V00158D, V00158S, V00181A, V00181D, V00181S, V00182A, V00182D, V00182S, V00188A, V00188D, V00188S, V00211A, V00211D, V00211S, V00212A, V00212D, V00212S, V00218A, V00218D, V00218S, V00221A, V00221D, V00221S, V00222A, V00222D, V00222S, V00228A, V00228D, V00228S, V00281A, V00281D, V00281S, V00282A, V00282D, V00282S, V00288A, V00288D, V00288S, V00311A, V00311D, V00311S, V00312A, V00312D, V00312S, V00318A, V00318D, V00318S, V00321A, V00321D, V00321S, V00322A, V00322D, V00322S, V00328A, V00328D, V00328S, V00381A, V00381D, V00381S, V00382A, V00382D, V00382S, V00388A, V00388D, V00388S, V00811A, V00811D, V00811S, V00812A, V00812D, V00812S, V00818A, V00818D, V00818S, V00821A, V00821D, V00821S, V00822A, V00822D, V00822S, V00828A, V00828D, V00828S, V00831A, V00831D, V00831S, V00832A, V00832D, V00832S, V00838A, V00838D, V00838S, V00841A, V00841D, V00841S, V00842A, V00842D, V00842S, V00848A, V00848D, V00848S, V00891A, V00891D, V00891S, V00892A, V00892D, V00892S, V00898A, V00898D, V00898S, V0100XA, V0100XD, V0100XS, V0101XA, V0101XD, V0101XS, V0102XA, V0102XD, V0102XS, V01031A, V01031D, V01031S, V01038A, V01038D, V01038S, V0109XA, V0109XD, V0109XS, V0110XA, V0110XD, V0110XS,

	V0111XA, V0111XD, V0111XS, V0112XA, V0112XD, V0112XS, V01131A, V01131D, V01131S, V01138A, V01138D, V01138S, V0119XA, V0119XD, V0119XS, V0190XA, V0190XD, V0190XS, V0191XA, V0191XD, V0191XS, V0192XA, V0192XD, V0192XS, V01931A, V01931D, V01931S, V01938A, V01938D, V01938S, V0199XA, V0199XD, V0199XS, V0200XA, V0200XD, V0200XS, V0201XA, V0201XD, V0201XS, V0202XA, V0202XD, V0202XS, V02031A, V02031D, V02031S, V02038A, V02038D, V02038S, V0209XA, V0209XD, V0209XS, V0210XA, V0210XD, V0210XS, V0211XA, V0211XD, V0211XS, V0212XA, V0212XD, V0212XS, V02131A, V02131D, V02131S, V02138A, V02138D, V02138S, V0219XA, V0219XD, V0219XS, V0290XA, V0290XD, V0290XS, V0291XA, V0291XD, V0291XS, V0292XA, V0292XD, V0292XS, V02931A, V02931D, V02931S, V02938A, V02938D, V02938S, V0299XA, V0299XD, V0299XS, V0300XA, V0300XD, V0300XS, V0301XA, V0301XD, V0301XS, V0302XA, V0302XD, V0302XS, V03031A, V03031D, V03031S, V03038A, V03038D, V03038S, V0309XA, V0309XD, V0309XS, V0310XA, V0310XD, V0310XS, V0311XA, V0311XD, V0311XS, V0312XA, V0312XD, V0312XS, V03131A, V03131D, V03131S, V03138A, V03138D, V03138S, V0319XA, V0319XD, V0319XS, V0390XA, V0390XD, V0390XS, V0391XA, V0391XD, V0391XS, V0392XA, V0392XD, V0392XS, V03931A, V03931D, V03931S, V03938A, V03938D, V03938S, V0399XA, V0399XD, V0399XS, V0400XA, V0400XD, V0400XS, V0401XA, V0401XD, V0401XS, V0402XA, V0402XD, V0402XS, V04031A, V04031D, V04031S, V04038A, V04038D, V04038S, V0409XA, V0409XD, V0409XS, V0410XA, V0410XD, V0410XS, V0411XA, V0411XD, V0411XS, V0412XA, V0412XD, V0412XS, V04131A, V04131D, V04131S, V04138A, V04138D, V04138S, V0419XA, V0419XD, V0419XS, V0490XA, V0490XD, V0490XS, V0491XA, V0491XD, V0491XS, V0492XA, V0492XD, V0492XS, V04931A, V04931D, V04931S, V04938A, V04938D, V04938S, V0499XA, V0499XD, V0499XS, V0500XA, V0500XD, V0500XS, V0501XA, V0501XD, V0501XS, V0502XA, V0502XD, V0502XS, V05031A, V05031D, V05031S, V05038A, V05038D, V05038S, V0509XA, V0509XD, V0509XS, V0510XA, V0510XD, V0510XS, V0511XA, V0511XD, V0511XS, V0512XA, V0512XD, V0512XS, V05131A, V05131D, V05131S, V05138A, V05138D, V05138S, V0519XA, V0519XD, V0519XS, V0590XA, V0590XD, V0590XS, V0591XA, V0591XD, V0591XS, V0592XA, V0592XD, V0592XS, V05931A, V05931D, V05931S, V05938A, V05938D, V05938S, V0599XA, V0599XD, V0599XS, V0600XA, V0600XD, V0600XS, V0601XA, V0601XD, V0601XS, V0602XA, V0602XD, V0602XS, V06031A, V06031D, V06031S, V06038A, V06038D, V06038S, V0609XA, V0609XD, V0609XS, V0610XA, V0610XD, V0610XS, V0611XA, V0611XD, V0611XS, V0612XA, V0612XD, V0612XS, V06131A, V06131D, V06131S, V06138A, V06138D, V06138S, V0619XA, V0619XD, V0619XS, V0690XA, V0690XD, V0690XS, V0691XA, V0691XD, V0691XS, V0692XA, V0692XD, V0692XS, V06931A, V06931D, V06931S, V06938A, V06938D, V06938S, V0699XA, V0699XD, V0699XS, V0900XA, V0900XD, V0900XS, V0901XA, V0901XD,

	V0901XS, V0909XA, V0909XD, V0909XS, V091XXA, V091XXD, V091XXS, V0920XA, V0920XD, V0920XS, V0921XA, V0921XD, V0921XS, V0929XA, V0929XD, V0929XS, V093XXA, V093XXD, V093XXS, V099XXA, V099XXD, V099XXS, V100XXA, V100XXD, V100XXS, V101XXA, V101XXD, V101XXS, V102XXA, V102XXD, V102XXS, V103XXA, V103XXD, V103XXS, V104XXA, V104XXD, V104XXS, V105XXA, V105XXD, V105XXS, V109XXA, V109XXD, V109XXS, V110XXA, V110XXD, V110XXS, V111XXA, V111XXD, V111XXS, V112XXA, V112XXD, V112XXS, V113XXA, V113XXD, V113XXS, V114XXA, V114XXD, V114XXS, V115XXA, V115XXD, V115XXS, V119XXA, V119XXD, V119XXS, V120XXA, V120XXD, V120XXS, V121XXA, V121XXD, V121XXS, V122XXA, V122XXD, V122XXS, V123XXA, V123XXD, V123XXS, V124XXA, V124XXD, V124XXS, V125XXA, V125XXD, V125XXS, V129XXA, V129XXD, V129XXS, V130XXA, V130XXD, V130XXS, V131XXA, V131XXD, V131XXS, V132XXA, V132XXD, V132XXS, V133XXA, V133XXD, V133XXS, V134XXA, V134XXD, V134XXS, V135XXA, V135XXD, V135XXS, V139XXA, V139XXD, V139XXS, V140XXA, V140XXD, V140XXS, V141XXA, V141XXD, V141XXS, V142XXA, V142XXD, V142XXS, V143XXA, V143XXD, V143XXS, V144XXA, V144XXD, V144XXS, V145XXA, V145XXD, V145XXS, V149XXA, V149XXD, V149XXS, V150XXA, V150XXD, V150XXS, V151XXA, V151XXD, V151XXS, V152XXA, V152XXD, V152XXS, V153XXA, V153XXD, V153XXS, V154XXA, V154XXD, V154XXS, V155XXA, V155XXD, V155XXS, V159XXA, V159XXD, V159XXS, V160XXA, V160XXD, V160XXS, V161XXA, V161XXD, V161XXS, V162XXA, V162XXD, V162XXS, V163XXA, V163XXD, V163XXS, V164XXA, V164XXD, V164XXS, V165XXA, V165XXD, V165XXS, V169XXA, V169XXD, V169XXS, V170XXA, V170XXD, V170XXS, V171XXA, V171XXD, V171XXS, V172XXA, V172XXD, V172XXS, V173XXA, V173XXD, V173XXS, V174XXA, V174XXD, V174XXS, V175XXA, V175XXD, V175XXS, V179XXA, V179XXD, V179XXS, V180XXA, V180XXD, V180XXS, V181XXA, V181XXD, V181XXS, V182XXA, V182XXD, V182XXS, V183XXA, V183XXD, V183XXS, V184XXA, V184XXD, V184XXS, V185XXA, V185XXD, V185XXS, V189XXA, V189XXD, V189XXS, V1900XA, V1900XD, V1900XS, V1909XA, V1909XD, V1909XS, V1910XA, V1910XD, V1910XS, V1919XA, V1919XD, V1919XS, V1920XA, V1920XD, V1920XS, V1929XA, V1929XD, V1929XS, V193XXA, V193XXD, V193XXS, V1940XA, V1940XD, V1940XS, V1949XA, V1949XD, V1949XS, V1950XA, V1950XD, V1950XS, V1959XA, V1959XD, V1959XS, V1960XA, V1960XD, V1960XS, V1969XA, V1969XD, V1969XS, V1981XA, V1981XD, V1981XS, V1988XA, V1988XD, V1988XS, V199XXA, V199XXD, V199XXS, V200XXA, V200XXD, V200XXS, V201XXA, V201XXD, V201XXS, V202XXA, V202XXD, V202XXS, V203XXA, V203XXD, V203XXS,

	V204XXA, V204XXD, V204XXS, V205XXA, V205XXD, V205XXS, V209XXA, V209XXD, V209XXS, V210XXA, V210XXD, V210XXS, V211XXA, V211XXD, V211XXS, V212XXA, V212XXD, V212XXS, V213XXA, V213XXD, V213XXS, V214XXA, V214XXD, V214XXS, V215XXA, V215XXD, V215XXS, V219XXA, V219XXD, V219XXS, V220XXA, V220XXD, V220XXS, V221XXA, V221XXD, V221XXS, V222XXA, V222XXD, V222XXS, V223XXA, V223XXD, V223XXS, V224XXA, V224XXD, V224XXS, V225XXA, V225XXD, V225XXS, V229XXA, V229XXD, V229XXS, V230XXA, V230XXD, V230XXS, V231XXA, V231XXD, V231XXS, V232XXA, V232XXD, V232XXS, V233XXA, V233XXD, V233XXS, V234XXA, V234XXD, V234XXS, V235XXA, V235XXD, V235XXS, V239XXA, V239XXD, V239XXS, V240XXA, V240XXD, V240XXS, V241XXA, V241XXD, V241XXS, V242XXA, V242XXD, V242XXS, V243XXA, V243XXD, V243XXS, V244XXA, V244XXD, V244XXS, V245XXA, V245XXD, V245XXS, V249XXA, V249XXD, V249XXS, V250XXA, V250XXD, V250XXS, V251XXA, V251XXD, V251XXS, V252XXA, V252XXD, V252XXS, V253XXA, V253XXD, V253XXS, V254XXA, V254XXD, V254XXS, V255XXA, V255XXD, V255XXS, V259XXA, V259XXD, V259XXS, V260XXA, V260XXD, V260XXS, V261XXA, V261XXD, V261XXS, V262XXA, V262XXD, V262XXS, V263XXA, V263XXD, V263XXS, V264XXA, V264XXD, V264XXS, V265XXA, V265XXD, V265XXS, V269XXA, V269XXD, V269XXS, V270XXA, V270XXD, V270XXS, V271XXA, V271XXD, V271XXS, V272XXA, V272XXD, V272XXS, V273XXA, V273XXD, V273XXS, V274XXA, V274XXD, V274XXS, V275XXA, V275XXD, V275XXS, V279XXA, V279XXD, V279XXS, V280XXA, V280XXD, V280XXS, V281XXA, V281XXD, V281XXS, V282XXA, V282XXD, V282XXS, V283XXA, V283XXD, V283XXS, V284XXA, V284XXD, V284XXS, V285XXA, V285XXD, V285XXS, V289XXA, V289XXD, V289XXS, V2900XA, V2900XD, V2900XS, V2909XA, V2909XD, V2909XS, V2910XA, V2910XD, V2910XS, V2919XA, V2919XD, V2919XS, V2920XA, V2920XD, V2920XS, V2929XA, V2929XD, V2929XS, V293XXA, V293XXD, V293XXS, V2940XA, V2940XD, V2940XS, V2949XA, V2949XD, V2949XS, V2950XA, V2950XD, V2950XS, V2959XA, V2959XD, V2959XS, V2960XA, V2960XD, V2960XS, V2969XA, V2969XD, V2969XS, V2981XA, V2981XD, V2981XS, V2988XA, V2988XD, V2988XS, V299XXA, V299XXD, V299XXS, V300XXA, V300XXD, V300XXS, V301XXA, V301XXD, V301XXS, V302XXA, V302XXD, V302XXS, V303XXA, V303XXD, V303XXS, V304XXA, V304XXD, V304XXS, V305XXA, V305XXD, V305XXS, V306XXA, V306XXD, V306XXS, V307XXA, V307XXD, V307XXS, V309XXA, V309XXD, V309XXS, V310XXA, V310XXD, V310XXS, V311XXA, V311XXD, V311XXS, V312XXA, V312XXD, V312XXS, V313XXA, V313XXD, V313XXS, V314XXA, V314XXD, V314XXS, V315XXA, V315XXD,

	V315XXS, V316XXA, V316XXD, V316XXS, V317XXA, V317XXD, V317XXS, V319XXA, V319XXD, V319XXS, V320XXA, V320XXD, V320XXS, V321XXA, V321XXD, V321XXS, V322XXA, V322XXD, V322XXS, V323XXA, V323XXD, V323XXS, V324XXA, V324XXD, V324XXS, V325XXA, V325XXD, V325XXS, V326XXA, V326XXD, V326XXS, V327XXA, V327XXD, V327XXS, V329XXA, V329XXD, V329XXS, V330XXA, V330XXD, V330XXS, V331XXA, V331XXD, V331XXS, V332XXA, V332XXD, V332XXS, V333XXA, V333XXD, V333XXS, V334XXA, V334XXD, V334XXS, V335XXA, V335XXD, V335XXS, V336XXA, V336XXD, V336XXS, V337XXA, V337XXD, V337XXS, V339XXA, V339XXD, V339XXS, V340XXA, V340XXD, V340XXS, V341XXA, V341XXD, V341XXS, V342XXA, V342XXD, V342XXS, V343XXA, V343XXD, V343XXS, V344XXA, V344XXD, V344XXS, V345XXA, V345XXD, V345XXS, V346XXA, V346XXD, V346XXS, V347XXA, V347XXD, V347XXS, V349XXA, V349XXD, V349XXS, V350XXA, V350XXD, V350XXS, V351XXA, V351XXD, V351XXS, V352XXA, V352XXD, V352XXS, V353XXA, V353XXD, V353XXS, V354XXA, V354XXD, V354XXS, V355XXA, V355XXD, V355XXS, V356XXA, V356XXD, V356XXS, V357XXA, V357XXD, V357XXS, V359XXA, V359XXD, V359XXS, V360XXA, V360XXD, V360XXS, V361XXA, V361XXD, V361XXS, V362XXA, V362XXD, V362XXS, V363XXA, V363XXD, V363XXS, V364XXA, V364XXD, V364XXS, V365XXA, V365XXD, V365XXS, V366XXA, V366XXD, V366XXS, V367XXA, V367XXD, V367XXS, V369XXA, V369XXD, V369XXS, V370XXA, V370XXD, V370XXS, V371XXA, V371XXD, V371XXS, V372XXA, V372XXD, V372XXS, V373XXA, V373XXD, V373XXS, V374XXA, V374XXD, V374XXS, V375XXA, V375XXD, V375XXS, V376XXA, V376XXD, V376XXS, V377XXA, V377XXD, V377XXS, V379XXA, V379XXD, V379XXS, V380XXA, V380XXD, V380XXS, V381XXA, V381XXD, V381XXS, V382XXA, V382XXD, V382XXS, V383XXA, V383XXD, V383XXS, V384XXA, V384XXD, V384XXS, V385XXA, V385XXD, V385XXS, V386XXA, V386XXD, V386XXS, V387XXA, V387XXD, V387XXS, V389XXA, V389XXD, V389XXS, V3900XA, V3900XD, V3900XS, V3909XA, V3909XD, V3909XS, V3910XA, V3910XD, V3910XS, V3919XA, V3919XD, V3919XS, V3920XA, V3920XD, V3920XS, V3929XA, V3929XD, V3929XS, V393XXA, V393XXD, V393XXS, V3940XA, V3940XD, V3940XS, V3949XA, V3949XD, V3949XS, V3950XA, V3950XD, V3950XS, V3959XA, V3959XD, V3959XS, V3960XA, V3960XD, V3960XS, V3969XA, V3969XD, V3969XS, V3981XA, V3981XD, V3981XS, V3989XA, V3989XD, V3989XS, V399XXA, V399XXD, V399XXS, V400XXA, V400XXD, V400XXS, V401XXA, V401XXD, V401XXS, V402XXA, V402XXD, V402XXS, V403XXA, V403XXD, V403XXS, V404XXA, V404XXD, V404XXS, V405XXA, V405XXD, V405XXS, V406XXA, V406XXD, V406XXS, V407XXA,

	V407XXD, V407XXS, V409XXA, V409XXD, V409XXS, V410XXA, V410XXD, V410XXS, V411XXA, V411XXD, V411XXS, V412XXA, V412XXD, V412XXS, V413XXA, V413XXD, V413XXS, V414XXA, V414XXD, V414XXS, V415XXA, V415XXD, V415XXS, V416XXA, V416XXD, V416XXS, V417XXA, V417XXD, V417XXS, V419XXA, V419XXD, V419XXS, V420XXA, V420XXD, V420XXS, V421XXA, V421XXD, V421XXS, V422XXA, V422XXD, V422XXS, V423XXA, V423XXD, V423XXS, V424XXA, V424XXD, V424XXS, V425XXA, V425XXD, V425XXS, V426XXA, V426XXD, V426XXS, V427XXA, V427XXD, V427XXS, V429XXA, V429XXD, V429XXS, V4301XA, V4301XD, V4301XS, V4302XA, V4302XD, V4302XS, V4303XA, V4303XD, V4303XS, V4304XA, V4304XD, V4304XS, V4311XA, V4311XD, V4311XS, V4312XA, V4312XD, V4312XS, V4313XA, V4313XD, V4313XS, V4314XA, V4314XD, V4314XS, V4321XA, V4321XD, V4321XS, V4322XA, V4322XD, V4322XS, V4323XA, V4323XD, V4323XS, V4324XA, V4324XD, V4324XS, V4331XA, V4331XD, V4331XS, V4332XA, V4332XD, V4332XS, V4333XA, V4333XD, V4333XS, V4334XA, V4334XD, V4334XS, V4341XA, V4341XD, V4341XS, V4342XA, V4342XD, V4342XS, V4343XA, V4343XD, V4343XS, V4344XA, V4344XD, V4344XS, V4351XA, V4351XD, V4351XS, V4352XA, V4352XD, V4352XS, V4353XA, V4353XD, V4353XS, V4354XA, V4354XD, V4354XS, V4361XA, V4361XD, V4361XS, V4362XA, V4362XD, V4362XS, V4363XA, V4363XD, V4363XS, V4364XA, V4364XD, V4364XS, V4371XA, V4371XD, V4371XS, V4372XA, V4372XD, V4372XS, V4373XA, V4373XD, V4373XS, V4374XA, V4374XD, V4374XS, V4391XA, V4391XD, V4391XS, V4392XA, V4392XD, V4392XS, V4393XA, V4393XD, V4393XS, V4394XA, V4394XD, V4394XS, V440XXA, V440XXD, V440XXS, V441XXA, V441XXD, V441XXS, V442XXA, V442XXD, V442XXS, V443XXA, V443XXD, V443XXS, V444XXA, V444XXD, V444XXS, V445XXA, V445XXD, V445XXS, V446XXA, V446XXD, V446XXS, V447XXA, V447XXD, V447XXS, V449XXA, V449XXD, V449XXS, V450XXA, V450XXD, V450XXS, V451XXA, V451XXD, V451XXS, V452XXA, V452XXD, V452XXS, V453XXA, V453XXD, V453XXS, V454XXA, V454XXD, V454XXS, V455XXA, V455XXD, V455XXS, V456XXA, V456XXD, V456XXS, V457XXA, V457XXD, V457XXS, V459XXA, V459XXD, V459XXS, V460XXA, V460XXD, V460XXS, V461XXA, V461XXD, V461XXS, V462XXA, V462XXD, V462XXS, V463XXA, V463XXD, V463XXS, V464XXA, V464XXD, V464XXS, V465XXA, V465XXD, V465XXS, V466XXA, V466XXD, V466XXS, V467XXA, V467XXD, V467XXS, V469XXA, V469XXD, V469XXS, V4701XA, V4701XD, V4701XS, V4702XA, V4702XD, V4702XS, V470XXA, V470XXD, V470XXS, V4711XA, V4711XD, V4711XS, V4712XA, V4712XD, V4712XS, V471XXA, V471XXD, V471XXS, V472XXA, V472XXD, V472XXS, V4731XA, V4731XD, V4731XS, V4732XA, V4732XD, V4732XS, V473XXA, V473XXD, V473XXS, V474XXA, V474XXD, V474XXS,

	V4751XA, V4751XD, V4751XS, V4752XA, V4752XD, V4752XS, V475XXA, V475XXD, V475XXS, V4761XA, V4761XD, V4761XS, V4762XA, V4762XD, V4762XS, V476XXA, V476XXD, V476XXS, V477XXA, V477XXD, V477XXS, V4791XA, V4791XD, V4791XS, V4792XA, V4792XD, V4792XS, V479XXA, V479XXD, V479XXS, V480XXA, V480XXD, V480XXS, V481XXA, V481XXD, V481XXS, V482XXA, V482XXD, V482XXS, V483XXA, V483XXD, V483XXS, V484XXA, V484XXD, V484XXS, V485XXA, V485XXD, V485XXS, V486XXA, V486XXD, V486XXS, V487XXA, V487XXD, V487XXS, V489XXA, V489XXD, V489XXS, V4900XA, V4900XD, V4900XS, V4909XA, V4909XD, V4909XS, V4910XA, V4910XD, V4910XS, V4919XA, V4919XD, V4919XS, V4920XA, V4920XD, V4920XS, V4929XA, V4929XD, V4929XS, V493XXA, V493XXD, V493XXS, V4940XA, V4940XD, V4940XS, V4949XA, V4949XD, V4949XS, V4950XA, V4950XD, V4950XS, V4959XA, V4959XD, V4959XS, V4960XA, V4960XD, V4960XS, V4969XA, V4969XD, V4969XS, V4981XA, V4981XD, V4981XS, V4988XA, V4988XD, V4988XS, V499XXA, V499XXD, V499XXS, V500XXA, V500XXD, V500XXS, V501XXA, V501XXD, V501XXS, V502XXA, V502XXD, V502XXS, V503XXA, V503XXD, V503XXS, V504XXA, V504XXD, V504XXS, V505XXA, V505XXD, V505XXS, V506XXA, V506XXD, V506XXS, V507XXA, V507XXD, V507XXS, V509XXA, V509XXD, V509XXS, V510XXA, V510XXD, V510XXS, V511XXA, V511XXD, V511XXS, V512XXA, V512XXD, V512XXS, V513XXA, V513XXD, V513XXS, V514XXA, V514XXD, V514XXS, V515XXA, V515XXD, V515XXS, V516XXA, V516XXD, V516XXS, V517XXA, V517XXD, V517XXS, V519XXA, V519XXD, V519XXS, V520XXA, V520XXD, V520XXS, V521XXA, V521XXD, V521XXS, V522XXA, V522XXD, V522XXS, V523XXA, V523XXD, V523XXS, V524XXA, V’24XXD, V524XXS, V525XXA, V’25XXD, V525XXS, V526XXA, V’26XXD, V526XXS, V527XXA, V527XXD, V527XXS, V529XXA, V529XXD, V529XXS, V530XXA, V530XXD, V530XXS, V531XXA, V531XXD, V531XXS, V532XXA, V532XXD, V532XXS, V533XXA, V533XXD, V533XXS, V534XXA, V534XXD, V534XXS, V535XXA, V535XXD, V535XXS, V536XXA, V536XXD, V536XXS, V537XXA, V537XXD, V537XXS, V539XXA, V539XXD, V539XXS, V540XXA, V540XXD, V540XXS, V541XXA, V541XXD, V541XXS, V542XXA, V542XXD, V542XXS, V543XXA, V543XXD, V543XXS, V544XXA, V544XXD, V544XXS, V545XXA, V545XXD, V545XXS, V546XXA, V546XXD, V546XXS, V547XXA, V547XXD, V547XXS, V549XXA, V549XXD, V549XXS, V550XXA, V550XXD, V550XXS, V551XXA, V551XXD, V551XXS, V552XXA, V552XXD, V552XXS, V553XXA, V553XXD, V553XXS, V554XXA, V554XXD, V554XXS, V555XXA, V555XXD, V555XXS, V556XXA, V556XXD, V556XXS, V557XXA, V557XXD, V557XXS, V559XXA, V559XXD, V559XXS, V560XXA, V560XXD, V560XXS, V561XXA,

	V561XXD, V561XXS, V562XXA, V562XXD, V562XXS, V563XXA, V563XXD, V563XXS, V564XXA, V564XXD, V564XXS, V565XXA, V565XXD, V565XXS, V566XXA, V566XXD, V566XXS, V567XXA, V567XXD, V567XXS, V569XXA, V569XXD, V569XXS, V570XXA, V570XXD, V570XXS, V571XXA, V571XXD, V571XXS, V572XXA, V572XXD, V572XXS, V573XXA, V573XXD, V573XXS, V574XXA, V574XXD, V574XXS, V575XXA, V575XXD, V575XXS, V576XXA, V576XXD, V576XXS, V577XXA, V577XXD, V577XXS, V579XXA, V579XXD, V579XXS, V580XXA, V580XXD, V580XXS, V581XXA, V581XXD, V581XXS, V582XXA, V582XXD, V582XXS, V583XXA, V583XXD, V583XXS, V584XXA, V584XXD, V584XXS, V585XXA, V585XXD, V585XXS, V586XXA, V586XXD, V586XXS, V587XXA, V587XXD, V587XXS, V589XXA, V589XXD, V589XXS, V5900XA, V5900XD, V5900XS, V5909XA, V5909XD, V5909XS, V5910XA, V5910XD, V5910XS, V5919XA, V5919XD, V5919XS, V5920XA, V5920XD, V5920XS, V5929XA, V5929XD, V5929XS, V593XXA, V593XXD, V593XXS, V5940XA, V5940XD, V5940XS, V5949XA, V5949XD, V5949XS, V5950XA, V5950XD, V5950XS, V5959XA, V5959XD, V5959XS, V5960XA, V5960XD, V5960XS, V5969XA, V5969XD, V5969XS, V5981XA, V5981XD, V5981XS, V5988XA, V5988XD, V5988XS, V599XXA, V599XXD, V599XXS, V600XXA, V600XXD, V600XXS, V601XXA, V601XXD, V601XXS, V602XXA, V602XXD, V602XXS, V603XXA, V603XXD, V603XXS, V604XXA, V604XXD, V604XXS, V605XXA, V605XXD, V605XXS, V606XXA, V606XXD, V606XXS, V607XXA, V607XXD, V607XXS, V609XXA, V609XXD, V609XXS, V610XXA, V610XXD, V610XXS, V611XXA, V611XXD, V611XXS, V612XXA, V612XXD, V612XXS, V613XXA, V613XXD, V613XXS, V614XXA, V614XXD, V614XXS, V615XXA, V615XXD, V615XXS, V616XXA, V616XXD, V616XXS, V617XXA, V617XXD, V617XXS, V619XXA, V619XXD, V619XXS, V620XXA, V620XXD, V620XXS, V621XXA, V621XXD, V621XXS, V622XXA, V622XXD, V622XXS, V623XXA, V623XXD, V623XXS, V624XXA, V624XXD, V624XXS, V625XXA, V625XXD, V625XXS, V626XXA, V626XXD, V626XXS, V627XXA, V627XXD, V627XXS, V629XXA, V629XXD, V629XXS, V630XXA, V630XXD, V630XXS, V631XXA, V631XXD, V631XXS, V632XXA, V632XXD, V632XXS, V633XXA, V633XXD, V633XXS, V634XXA, V634XXD, V634XXS, V635XXA, V635XXD, V635XXS, V636XXA, V636XXD, V636XXS, V637XXA, V637XXD, V637XXS, V639XXA, V639XXD, V639XXS, V640XXA, V640XXD, V640XXS, V641XXA, V641XXD, V641XXS, V642XXA, V642XXD, V642XXS, V643XXA, V643XXD, V643XXS, V644XXA, V644XXD, V644XXS, V645XXA, V645XXD, V645XXS, V646XXA, V646XXD, V646XXS, V647XXA, V647XXD, V647XXS, V649XXA, V649XXD, V649XXS, V650XXA, V650XXD, V650XXS, V651XXA, V651XXD, V651XXS, V652XXA, V652XXD, V652XXS,

	V653XXA, V653XXD, V653XXS, V654XXA, V654XXD, V654XXS, V655XXA, V655XXD, V655XXS, V656XXA, V656XXD, V656XXS, V657XXA, V657XXD, V657XXS, V659XXA, V659XXD, V659XXS, V660XXA, V660XXD, V660XXS, V661XXA, V661XXD, V661XXS, V662XXA, V662XXD, V662XXS, V663XXA, V663XXD, V663XXS, V664XXA, V664XXD, V664XXS, V665XXA, V665XXD, V665XXS, V666XXA, V666XXD, V666XXS, V667XXA, V667XXD, V667XXS, V669XXA, V669XXD, V669XXS, V670XXA, V670XXD, V670XXS, V671XXA, V671XXD, V671XXS, V672XXA, V672XXD, V672XXS, V673XXA, V673XXD, V673XXS, V674XXA, V674XXD, V674XXS, V675XXA, V675XXD, V675XXS, V676XXA, V676XXD, V676XXS, V677XXA, V677XXD, V677XXS, V679XXA, V679XXD, V679XXS, V680XXA, V680XXD, V680XXS, V681XXA, V681XXD, V681XXS, V682XXA, V682XXD, V682XXS, V683XXA, V683XXD, V683XXS, V684XXA, V684XXD, V684XXS, V685XXA, V685XXD, V685XXS, V686XXA, V686XXD, V686XXS, V687XXA, V687XXD, V687XXS, V689XXA, V689XXD, V689XXS, V6900XA, V6900XD, V6900XS, V6909XA, V6909XD, V6909XS, V6910XA, V6910XD, V6910XS, V6919XA, V6919XD, V6919XS, V6920XA, V6920XD, V6920XS, V6929XA, V6929XD, V6929XS, V693XXA, V693XXD, V693XXS, V6940XA, V6940XD, V6940XS, V6949XA, V6949XD, V6949XS, V6950XA, V6950XD, V6950XS, V6959XA, V6959XD, V6959XS, V6960XA, V6960XD, V6960XS, V6969XA, V6969XD, V6969XS, V6981XA, V6981XD, V6981XS, V6988XA, V6988XD, V6988XS, V699XXA, V699XXD, V699XXS, V700XXA, V700XXD, V700XXS, V701XXA, V701XXD, V701XXS, V702XXA, V702XXD, V702XXS, V703XXA, V703XXD, V703XXS, V704XXA, V704XXD, V704XXS, V705XXA, V705XXD, V705XXS, V706XXA, V706XXD, V706XXS, V707XXA, V707XXD, V707XXS, V709XXA, V709XXD, V709XXS, V710XXA, V710XXD, V710XXS, V711XXA, V711XXD, V711XXS, V712XXA, V712XXD, V712XXS, V713XXA, V713XXD, V713XXS, V714XXA, V714XXD, V714XXS, V715XXA, V715XXD, V715XXS, V716XXA, V716XXD, V716XXS, V717XXA, V717XXD, V717XXS, V719XXA, V719XXD, V719XXS, V720XXA, V720XXD, V720XXS, V721XXA, V721XXD, V721XXS, V722XXA, V722XXD, V722XXS, V723XXA, V723XXD, V723XXS, V724XXA, V724XXD, V724XXS, V725XXA, V725XXD, V725XXS, V726XXA, V726XXD, V726XXS, V727XXA, V727XXD, V727XXS, V729XXA, V729XXD, V729XXS, V730XXA, V730XXD, V730XXS, V731XXA, V731XXD, V731XXS, V732XXA, V732XXD, V732XXS, V733XXA, V733XXD, V733XXS, V734XXA, V734XXD, V734XXS, V735XXA, V735XXD, V735XXS, V736XXA, V736XXD, V736XXS, V737XXA, V737XXD, V737XXS, V739XXA, V739XXD, V739XXS, V740XXA, V740XXD, V740XXS, V741XXA, V741XXD, V741XXS, V742XXA, V742XXD, V742XXS, V743XXA, V743XXD, V743XXS, V744XXA, V744XXD,

	V744XXS, V745XXA, V745XXD, V745XXS, V746XXA, V746XXD, V746XXS, V747XXA, V747XXD, V747XXS, V749XXA, V749XXD, V749XXS, V750XXA, V750XXD, V750XXS, V751XXA, V751XXD, V751XXS, V752XXA, V752XXD, V752XXS, V753XXA, V753XXD, V753XXS, V754XXA, V754XXD, V754XXS, V755XXA, V755XXD, V755XXS, V756XXA, V756XXD, V756XXS, V757XXA, V757XXD, V757XXS, V759XXA, V759XXD, V759XXS, V760XXA, V760XXD, V760XXS, V761XXA, V761XXD, V761XXS, V762XXA, V762XXD, V762XXS, V763XXA, V763XXD, V763XXS, V764XXA, V764XXD, V764XXS, V765XXA, V765XXD, V765XXS, V766XXA, V766XXD, V766XXS, V767XXA, V767XXD, V767XXS, V769XXA, V769XXD, V769XXS, V770XXA, V770XXD, V770XXS, V771XXA, V771XXD, V771XXS, V772XXA, V772XXD, V772XXS, V773XXA, V773XXD, V773XXS, V774XXA, V774XXD, V774XXS, V775XXA, V775XXD, V775XXS, V776XXA, V776XXD, V776XXS, V777XXA, V777XXD, V777XXS, V779XXA, V779XXD, V779XXS, V780XXA, V780XXD, V780XXS, V781XXA, V781XXD, V781XXS, V782XXA, V782XXD, V782XXS, V783XXA, V783XXD, V783XXS, V784XXA, V784XXD, V784XXS, V785XXA, V785XXD, V785XXS, V786XXA, V786XXD, V786XXS, V787XXA, V787XXD, V787XXS, V789XXA, V789XXD, V789XXS, V7900XA, V7900XD, V7900XS, V7909XA, V7909XD, V7909XS, V7910XA, V7910XD, V7910XS, V7919XA, V7919XD, V7919XS, V7920XA, V7920XD, V7920XS, V7929XA, V7929XD, V7929XS, V793XXA, V793XXD, V793XXS, V7940XA, V7940XD, V7940XS, V7949XA, V7949XD, V7949XS, V7950XA, V7950XD, V7950XS, V7959XA, V7959XD, V7959XS, V7960XA, V7960XD, V7960XS, V7969XA, V7969XD, V7969XS, V7981XA, V7981XD, V7981XS, V7988XA, V7988XD, V7988XS, V799XXA, V799XXD, V799XXS, V80010A, V80010D, V80010S, V80018A, V80018D, V80018S, V8002XA, V8002XD, V8002XS, V8011XA, V8011XD, V8011XS, V8012XA, V8012XD, V8012XS, V8021XA, V8021XD, V8021XS, V8022XA, V8022XD, V8022XS, V8031XA, V8031XD, V8031XS, V8032XA, V8032XD, V8032XS, V8041XA, V8041XD, V8041XS, V8042XA, V8042XD, V8042XS, V8051XA, V8051XD, V8051XS, V8052XA, V8052XD, V8052XS, V8061XA, V8061XD, V8061XS, V8062XA, V8062XD, V8062XS, V80710A, V80710D, V80710S, V80711A, V80711D, V80711S, V80720A, V80720D, V80720S, V80721A, V80721D, V80721S, V80730A, V80730D, V80730S, V80731A, V80731D, V80731S, V80790A, V80790D, V80790S, V80791A, V80791D, V80791S, V8081XA, V8081XD, V8081XS, V8082XA, V8082XD, V8082XS, V80910A, V80910D, V80910S, V80918A, V80918D, V80918S, V80919A, V80919D, V80919S, V80920A, V80920D, V80920S, V80928A, V80928D, V80928S, V80929A, V80929D, V80929S, V810XXA, V810XXD, V810XXS, V811XXA, V811XXD, V811XXS, V812XXA, V812XXD, V812XXS, V813XXA, V813XXD, V813XXS, V814XXA, V814XXD, V814XXS, V815XXA, V815XXD, V815XXS, V816XXA, V816XXD, V816XXS,

V817XXA, V817XXD, V817XXS, V8181XA, V8181XD, V8181XS, V8182XA, V8182XD, V8182XS, V8183XA, V8183XD, V8183XS, V8189XA, V8189XD, V8189XS, V819XXA, V819XXD, V819XXS, V820XXA, V820XXD, V820XXS, V821XXA, V821XXD, V821XXS, V822XXA, V822XXD, V822XXS, V823XXA, V823XXD, V823XXS, V824XXA, V824XXD, V824XXS, V825XXA, V825XXD, V825XXS, V826XXA, V826XXD, V826XXS, V827XXA, V827XXD, V827XXS, V828XXA, V828XXD, V828XXS, V829XXA, V829XXD, V829XXS, V830XXA, V830XXD, V830XXS, V831XXA, V831XXD, V831XXS, V832XXA, V832XXD, V832XXS, V833XXA, V833XXD, V833XXS, V834XXA, V834XXD, V834XXS, V835XXA, V835XXD, V835XXS, V836XXA, V836XXD, V836XXS, V837XXA, V837XXD, V837XXS, V839XXA, V839XXD, V839XXS, V840XXA, V840XXD, V840XXS, V841XXA, V841XXD, V841XXS, V842XXA, V842XXD, V842XXS, V843XXA, V843XXD, V843XXS, V844XXA, V844XXD, V844XXS, V845XXA, V845XXD, V845XXS, V846XXA, V846XXD, V846XXS, V847XXA, V847XXD, V847XXS, V849XXA, V849XXD, V849XXS, V850XXA, V850XXD, V850XXS, V851XXA, V851XXD, V851XXS, V852XXA, V852XXD, V852XXS, V853XXA, V853XXD, V853XXS, V854XXA, V854XXD, V854XXS, V855XXA, V855XXD, V855XXS, V856XXA, V856XXD, V856XXS, V857XXA, V857XXD, V857XXS, V859XXA, V859XXD, V859XXS, V8601XA, V8601XD, V8601XS, V8602XA, V8602XD, V8602XS, V8603XA, V8603XD, V8603XS, V8604XA, V8604XD, V8604XS, V8605XA, V8605XD, V8605XS, V8606XA, V8606XD, V8606XS, V8609XA, V8609XD, V8609XS, V8611XA, V8611XD, V8611XS, V8612XA, V8612XD, V8612XS, V8613XA, V8613XD, V8613XS, V8614XA, V8614XD, V8614XS, V8615XA, V8615XD, V8615XS, V8616XA, V8616XD, V8616XS, V8619XA, V8619XD, V8619XS, V8621XA, V8621XD, V8621XS, V8622XA, V8622XD, V8622XS, V8623XA, V8623XD, V8623XS, V8624XA, V8624XD, V8624XS, V8625XA, V8625XD, V8625XS, V8626XA, V8626XD, V8626XS, V8629XA, V8629XD, V8629XS, V8631XA, V8631XD, V8631XS, V8632XA, V8632XD, V8632XS, V8633XA, V8633XD, V8633XS, V8634XA, V8634XD, V8634XS, V8635XA, V8635XD, V8635XS, V8636XA, V8636XD, V8636XS, V8639XA, V8639XD, V8639XS, V8641XA, V8641XD, V8641XS, V8642XA, V8642XD, V8642XS, V8643XA, V8643XD, V8643XS, V8644XA, V8644XD, V8644XS, V8645XA, V8645XD, V8645XS, V8646XA, V8646XD, V8646XS, V8649XA, V8649XD, V8649XS, V8651XA, V8651XD, V8651XS, V8652XA, V8652XD, V8652XS, V8653XA, V8653XD, V8653XS, V8654XA, V8654XD, V8654XS, V8655XA, V8655XD, V8655XS, V8656XA, V8656XD, V8656XS, V8659XA, V8659XD, V8659XS, V8661XA, V8661XD, V8661XS, V8662XA, V8662XD, V8662XS, V8663XA, V8663XD, V8663XS, V8664XA, V8664XD, V8664XS, V8665XA, V8665XD, V8665XS, V8666XA, V8666XD, V8666XS, V8669XA, V8669XD, V8669XS, V8671XA, V8671XD, V8671XS, V8672XA, V8672XD, V8672XS, V8673XA, V8673XD, V8673XS, V8674XA, V8674XD,

	V8674XS, V8675XA, V8675XD, V8675XS, V8676XA, V8676XD, V8676XS, V8679XA, V8679XD, V8679XS, V8691XA, V8691XD, V8691XS, V8692XA, V8692XD, V8692XS, V8693XA, V8693XD, V8693XS, V8694XA, V8694XD, V8694XS, V8695XA, V8695XD, V8695XS, V8696XA, V8696XD, V8696XS, V8699XA, V8699XD, V8699XS, V870XXA, V870XXD, V870XXS, V871XXA, V871XXD, V871XXS, V872XXA, V872XXD, V872XXS, V873XXA, V873XXD, V873XXS, V874XXA, V874XXD, V874XXS, V875XXA, V875XXD, V875XXS, V876XXA, V876XXD, V876XXS, V877XXA, V877XXD, V877XXS, V878XXA, V878XXD, V878XXS, V879XXA, V879XXD, V879XXS, V880XXA, V880XXD, V880XXS, V881XXA, V881XXD, V881XXS, V882XXA, V882XXD, V882XXS, V883XXA, V883XXD, V883XXS, V884XXA, V884XXD, V884XXS, V885XXA, V885XXD, V885XXS, V886XXA, V886XXD, V886XXS, V887XXA, V887XXD, V887XXS, V888XXA, V888XXD, V888XXS, V889XXA, V889XXD, V889XXS, V890XXA, V890XXD, V890XXS, V891XXA, V891XXD, V891XXS, V892XXA, V892XXD, V892XXS, V893XXA, V893XXD, V893XXS, V899XXA, V899XXD, V899XXS, V9000XA, V9000XD, V9000XS, V9001XA, V9001XD, V9001XS, V9002XA, V9002XD, V9002XS, V9003XA, V9003XD, V9003XS, V9004XA, V9004XD, V9004XS, V9005XA, V9005XD, V9005XS, V9006XA, V9006XD, V9006XS, V9008XA, V9008XD, V9008XS, V9009XA, V9009XD, V9009XS, V9010XA, V9010XD, V9010XS, V9011XA, V9011XD, V9011XS, V9012XA, V9012XD, V9012XS, V9013XA, V9013XD, V9013XS, V9014XA, V9014XD, V9014XS, V9015XA, V9015XD, V9015XS, V9016XA, V9016XD, V9016XS, V9018XA, V9018XD, V9018XS, V9019XA, V9019XD, V9019XS, V9020XA, V9020XD, V9020XS, V9021XA, V9021XD, V9021XS, V9022XA, V9022XD, V9022XS, V9023XA, V9023XD, V9023XS, V9024XA, V9024XD, V9024XS, V9025XA, V9025XD, V9025XS, V9026XA, V9026XD, V9026XS, V9027XA, V9027XD, V9027XS, V9028XA, V9028XD, V9028XS, V9029XA, V9029XD, V9029XS, V9030XA, V9030XD, V9030XS, V9031XA, V9031XD, V9031XS, V9032XA, V9032XD, V9032XS, V9033XA, V9033XD, V9033XS, V9034XA, V9034XD, V9034XS, V9035XA, V9035XD, V9035XS, V9036XA, V9036XD, V9036XS, V9037XA, V9037XD, V9037XS, V9038XA, V9038XD, V9038XS, V9039XA, V9039XD, V9039XS, V9080XA, V9080XD, V9080XS, V9081XA, V9081XD, V9081XS, V9082XA, V9082XD, V9082XS, V9083XA, V9083XD, V9083XS, V9084XA, V9084XD, V9084XS, V9085XA, V9085XD, V9085XS, V9086XA, V9086XD, V9086XS, V9087XA, V9087XD, V9087XS, V9088XA, V9088XD, V9088XS, V9089XA, V9089XD, V9089XS, V9100XA, V9100XD, V9100XS, V9101XA, V9101XD, V9101XS, V9102XA, V9102XD, V9102XS, V9103XA, V9103XD, V9103XS, V9104XA, V9104XD, V9104XS, V9105XA, V9105XD, V9105XS, V9106XA, V9106XD, V9106XS, V9107XA, V9107XD, V9107XS, V9108XA, V9108XD, V9108XS, V9109XA, V9109XD, V9109XS, V9110XA, V9110XD, V9110XS, V9111XA, V9111XD, V9111XS, V9112XA, V9112XD, V9112XS, V9113XA, V9113XD, V9113XS, V9114XA, V9114XD, V9114XS,

	V9115XA, V9115XD, V9115XS, V9116XA, V9116XD, V9116XS, V9118XA, V9118XD, V9118XS, V9119XA, V9119XD, V9119XS, V9120XA, V9120XD, V9120XS, V9121XA, V9121XD, V9121XS, V9122XA, V9122XD, V9122XS, V9123XA, V9123XD, V9123XS, V9124XA, V9124XD, V9124XS, V9125XA, V9125XD, V9125XS, V9126XA, V9126XD, V9126XS, V9129XA, V9129XD, V9129XS, V9130XA, V9130XD, V9130XS, V9131XA, V9131XD, V9131XS, V9132XA, V9132XD, V9132XS, V9133XA, V9133XD, V9133XS, V9134XA, V9134XD, V9134XS, V9135XA, V9135XD, V9135XS, V9136XA, V9136XD, V9136XS, V9137XA, V9137XD, V9137XS, V9138XA, V9138XD, V9138XS, V9139XA, V9139XD, V9139XS, V9180XA, V9180XD, V9180XS, V9181XA, V9181XD, V9181XS, V9182XA, V9182XD, V9182XS, V9183XA, V9183XD, V9183XS, V9184XA, V9184XD, V9184XS, V9185XA, V9185XD, V9185XS, V9186XA, V9186XD, V9186XS, V9187XA, V9187XD, V9187XS, V9188XA, V9188XD, V9188XS, V9189XA, V9189XD, V9189XS, V9200XA, V9200XD, V9200XS, V9201XA, V9201XD, V9201XS, V9202XA, V9202XD, V9202XS, V9203XA, V9203XD, V9203XS, V9204XA, V9204XD, V9204XS, V9205XA, V9205XD, V9205XS, V9206XA, V9206XD, V9206XS, V9207XA, V9207XD, V9207XS, V9208XA, V9208XD, V9208XS, V9209XA, V9209XD, V9209XS, V9210XA, V9210XD, V9210XS, V9211XA, V9211XD, V9211XS, V9212XA, V9212XD, V9212XS, V9213XA, V9213XD, V9213XS, V9214XA, V9214XD, V9214XS, V9215XA, V9215XD, V9215XS, V9216XA, V9216XD, V9216XS, V9219XA, V9219XD, V9219XS, V9220XA, V9220XD, V9220XS, V9221XA, V9221XD, V9221XS, V9222XA, V9222XD, V9222XS, V9223XA, V9223XD, V9223XS, V9224XA, V9224XD, V9224XS, V9225XA, V9225XD, V9225XS, V9226XA, V9226XD, V9226XS, V9227XA, V9227XD, V9227XS, V9228XA, V9228XD, V9228XS, V9229XA, V9229XD, V9229XS, V9300XA, V9300XD, V9300XS, V9301XA, V9301XD, V9301XS, V9302XA, V9302XD, V9302XS, V9303XA, V9303XD, V9303XS, V9304XA, V9304XD, V9304XS, V9309XA, V9309XD, V9309XS, V9310XA, V9310XD, V9310XS, V9311XA, V9311XD, V9311XS, V9312XA, V9312XD, V9312XS, V9313XA, V9313XD, V9313XS, V9314XA, V9314XD, V9314XS, V9319XA, V9319XD, V9319XS, V9320XA, V9320XD, V9320XS, V9321XA, V9321XD, V9321XS, V9322XA, V9322XD, V9322XS, V9323XA, V9323XD, V9323XS, V9324XA, V9324XD, V9324XS, V9329XA, V9329XD, V9329XS, V9330XA, V9330XD, V9330XS, V9331XA, V9331XD, V9331XS, V9332XA, V9332XD, V9332XS, V9333XA, V9333XD, V9333XS, V9334XA, V9334XD, V9334XS, V9335XA, V9335XD, V9335XS, V9336XA, V9336XD, V9336XS, V9338XA, V9338XD, V9338XS, V9339XA, V9339XD, V9339XS, V9340XA, V9340XD, V9340XS, V9341XA, V9341XD, V9341XS, V9342XA, V9342XD, V9342XS, V9343XA, V9343XD, V9343XS, V9344XA, V9344XD, V9344XS, V9348XA, V9348XD, V9348XS, V9349XA, V9349XD, V9349XS, V9350XA, V9350XD, V9350XS, V9351XA, V9351XD, V9351XS, V9352XA, V9352XD, V9352XS, V9353XA, V9353XD, V9353XS, V9354XA, V9354XD, V9354XS, V9359XA, V9359XD, V9359XS, V9360XA, V9360XD, V9360XS, V9361XA, V9361XD, V9361XS, V9362XA, V9362XD,

	V9362XS, V9363XA, V9363XD, V9363XS, V9364XA, V9364XD, V9364XS, V9369XA, V9369XD, V9369XS, V9380XA, V9380XD, V9380XS, V9381XA, V9381XD, V9381XS, V9382XA, V9382XD, V9382XS, V9383XA, V9383XD, V9383XS, V9384XA, V9384XD, V9384XS, V9385XA, V9385XD, V9385XS, V9386XA, V9386XD, V9386XS, V9387XA, V9387XD, V9387XS, V9388XA, V9388XD, V9388XS, V9389XA, V9389XD, V9389XS, V940XXA, V940XXD, V940XXS, V9411XA, V9411XD, V9411XS, V9412XA, V9412XD, V9412XS, V9421XA, V9421XD, V9421XS, V9422XA, V9422XD, V9422XS, V9431XA, V9431XD, V9431XS, V9432XA, V9432XD, V9432XS, V944XXA, V944XXD, V944XXS, V94810A, V94810D, V94810S, V94811A, V94811D, V94811S, V94818A, V94818D, V94818S, V9489XA, V9489XD, V9489XS, V949XXA, V949XXD, V949XXS, V9500XA, V9500XD, V9500XS, V9501XA, V9501XD, V9501XS, V9502XA, V9502XD, V9502XS, V9503XA, V9503XD, V9503XS, V9504XA, V9504XD, V9504XS, V9505XA, V9505XD, V9505XS, V9509XA, V9509XD, V9509XS, V9510XA, V9510XD, V9510XS, V9511XA, V9511XD, V9511XS, V9512XA, V9512XD, V9512XS, V9513XA, V9513XD, V9513XS, V9514XA, V9514XD, V9514XS, V9515XA, V9515XD, V9515XS, V9519XA, V9519XD, V9519XS, V9520XA, V9520XD, V9520XS, V9521XA, V9521XD, V9521XS, V9522XA, V9522XD, V9522XS, V9523XA, V9523XD, V9523XS, V9524XA, V9524XD, V9524XS, V9525XA, V9525XD, V9525XS, V9529XA, V9529XD, V9529XS, V9530XA, V9530XD, V9530XS, V9531XA, V9531XD, V9531XS, V9532XA, V9532XD, V9532XS, V9533XA, V9533XD, V9533XS, V9534XA, V9534XD, V9534XS, V9535XA, V9535XD, V9535XS, V9539XA, V9539XD, V9539XS, V9540XA, V9540XD, V9540XS, V9541XA, V9541XD, V9541XS, V9542XA, V9542XD, V9542XS, V9543XA, V9543XD, V9543XS, V9544XA, V9544XD, V9544XS, V9545XA, V9545XD, V9545XS, V9549XA, V9549XD, V9549XS, V958XXA, V958XXD, V958XXS, V959XXA, V959XXD, V959XXS, V9600XA, V9600XD, V9600XS, V9601XA, V9601XD, V9601XS, V9602XA, V9602XD, V9602XS, V9603XA, V9603XD, V9603XS, V9604XA, V9604XD, V9604XS, V9605XA, V9605XD, V9605XS, V9609XA, V9609XD, V9609XS, V9610XA, V9610XD, V9610XS, V9611XA, V9611XD, V9611XS, V9612XA, V9612XD, V9612XS, V9613XA, V9613XD, V9613XS, V9614XA, V9614XD, V9614XS, V9615XA, V9615XD, V9615XS, V9619XA, V9619XD, V9619XS, V9620XA, V9620XD, V9620XS, V9621XA, V9621XD, V9621XS, V9622XA, V9622XD, V9622XS, V9623XA, V9623XD, V9623XS, V9624XA, V9624XD, V9624XS, V9625XA, V9625XD, V9625XS, V9629XA, V9629XD, V9629XS, V968XXA, V968XXD, V968XXS, V969XXA, V969XXD, V969XXS, V970XXA, V970XXD, V970XXS, V971XXA, V971XXD, V971XXS, V9721XA, V9721XD, V9721XS, V9722XA, V9722XD, V9722XS, V9729XA, V9729XD, V9729XS, V9731XA, V9731XD, V9731XS, V9732XA, V9732XD, V9732XS, V9733XA, V9733XD, V9733XS, V9739XA, V9739XD, V9739XS, V97810A, V97810D, V97810S, V97811A, V97811D, V97811S, V97818A, V97818D, V97818S, V9789XA, V9789XD, V9789XS, V980XXA, V980XXD, V980XXS,

	V981XXA, V981XXD, V981XXS, V982XXA, V982XXD, V982XXS, V983XXA, V983XXD, V983XXS, V988XXA, V988XXD, V988XXS, V99XXXA, V99XXXD, V99XXXS, W1840XA, W1840XD, W1840XS, W1841XA, W1841XD, W1841XS, W1842XA, W1842XD, W1842XS, W1843XA, W1843XD, W1843XS, W1849XA, W1849XD, W1849XS, W200XXA, W200XXD, W200XXS, W201XXA, W201XXD, W201XXS, W208XXA, W208XXD, W208XXS, W2100XA, W2100XD, W2100XS, W2101XA, W2101XD, W2101XS, W2102XA, W2102XD, W2102XS, W2103XA, W2103XD, W2103XS, W2104XA, W2104XD, W2104XS, W2105XA, W2105XD, W2105XS, W2106XA, W2106XD, W2106XS, W2107XA, W2107XD, W2107XS, W2109XA, W2109XD, W2109XS, W2111XA, W2111XD, W2111XS, W2112XA, W2112XD, W2112XS, W2113XA, W2113XD, W2113XS, W2119XA, W2119XD, W2119XS, W21210A, W21210D, W21210S, W21211A, W21211D, W21211S, W21220A, W21220D, W21220S, W21221A, W21221D, W21221S, W2131XA, W2131XD, W2131XS, W2132XA, W2132XD, W2132XS, W2139XA, W2139XD, W2139XS, W214XXA, W214XXD, W214XXS, W2181XA, W2181XD, W2181XS, W2189XA, W2189XD, W2189XS, W219XXA, W219XXD, W219XXS, W2201XA, W2201XD, W2201XS, W2202XA, W2202XD, W2202XS, W2203XA, W2203XD, W2203XS, W22041A, W22041D, W22041S, W22042A, W22042D, W22042S, W2209XA, W2209XD, W2209XS, W2210XA, W2210XD, W2210XS, W2211XA, W2211XD, W2211XS, W2212XA, W2212XD, W2212XS, W2219XA, W2219XD, W2219XS, W228XXA, W228XXD, W228XXS, W230XXA, W230XXD, W230XXS, W231XXA, W231XXD, W231XXS, W240XXA, W240XXD, W240XXS, W241XXA, W241XXD, W241XXS, W25XXXA, W25XXXD, W25XXXS, W260XXA, W260XXD, W260XXS, W261XXA, W261XXD, W261XXS, W262XXA, W262XXD, W262XXS, W268XXA, W268XXD, W268XXS, W269XXA, W269XXD, W269XXS, W270XXA, W270XXD, W270XXS, W271XXA, W271XXD, W271XXS, W272XXA, W272XXD, W272XXS, W273XXA, W273XXD, W273XXS, W274XXA, W274XXD, W274XXS, W275XXA, W275XXD, W275XXS, W278XXA, W278XXD, W278XXS, W28XXXA, W28XXXD, W28XXXS, W290XXA, W290XXD, W290XXS, W291XXA, W291XXD, W291XXS, W292XXA, W292XXD, W292XXS, W293XXA, W293XXD, W293XXS, W294XXA, W294XXD, W294XXS, W298XXA, W298XXD, W298XXS, W300XXA, W300XXD, W300XXS, W301XXA, W301XXD, W301XXS, W302XXA, W302XXD, W302XXS, W303XXA, W303XXD, W303XXS, W3081XA, W3081XD, W3081XS, W3089XA, W3089XD, W3089XS, W309XXA, W309XXD, W309XXS, W310XXA, W310XXD, W310XXS, W311XXA, W311XXD, W311XXS, W312XXA, W312XXD, W312XXS, W313XXA, W313XXD, W313XXS, W3181XA, W3181XD, W3181XS, W3182XA, W3182XD, W3182XS, W3183XA, W3183XD, W3183XS, W3189XA, W3189XD, W3189XS, W319XXA, W319XXD, W319XXS,

	W320XXA, W320XXD, W320XXS, W321XXA, W321XXD, W321XXS, W3300XA, W3300XD, W3300XS, W3301XA, W3301XD, W3301XS, W3302XA, W3302XD, W3302XS, W3303XA, W3303XD, W3303XS, W3309XA, W3309XD, W3309XS, W3310XA, W3310XD, W3310XS, W3311XA, W3311XD, W3311XS, W3312XA, W3312XD, W3312XS, W3313XA, W3313XD, W3313XS, W3319XA, W3319XD, W3319XS, W3400XA, W3400XD, W3400XS, W34010A, W34010D, W34010S, W34011A, W34011D, W34011S, W34018A, W34018D, W34018S, W3409XA, W3409XD, W3409XS, W3410XA, W3410XD, W3410XS, W34110A, W34110D, W34110S, W34111A, W34111D, W34111S, W34118A, W34118D, W34118S, W3419XA, W3419XD, W3419XS, W35XXXA, W35XXXD, W35XXXS, W361XXA, W361XXD, W361XXS, W362XXA, W362XXD, W362XXS, W363XXA, W363XXD, W363XXS, W368XXA, W368XXD, W368XXS, W369XXA, W369XXD, W369XXS, W370XXA, W370XXD, W370XXS, W378XXA, W378XXD, W378XXS, W38XXXA, W38XXXD, W38XXXS, W39XXXA, W39XXXD, W39XXXS, W400XXA, W400XXD, W400XXS, W401XXA, W401XXD, W401XXS, W408XXA, W408XXD, W408XXS, W409XXA, W409XXD, W409XXS, W420XXA, W420XXD, W420XXS, W429XXA, W429XXD, W429XXS, W450XXA, W450XXD, W450XXS, W451XXA, W451XXD, W451XXS, W452XXA, W452XXD, W452XXS, W458XXA, W458XXD, W458XXS, W460XXA, W460XXD, W460XXS, W461XXA, W461XXD, W461XXS, W4901XA, W4901XD, W4901XS, W4902XA, W4902XD, W4902XS, W4903XA, W4903XD, W4903XS, W4904XA, W4904XD, W4904XS, W4909XA, W4909XD, W4909XS, W499XXA, W499XXD, W499XXS, W500XXA, W500XXD, W500XXS, W501XXA, W501XXD, W501XXS, W502XXA, W502XXD, W502XXS, W503XXA, W503XXD, W503XXS, W504XXA, W504XXD, W504XXS, W51XXXA, W51XXXD, W51XXXS, W52XXXA, W52XXXD, W52XXXS, W5301XA, W5301XD, W5301XS, W5309XA, W5309XD, W5309XS, W5311XA, W5311XD, W5311XS, W5319XA, W5319XD, W5319XS, W5321XA, W5321XD, W5321XS, W5329XA, W5329XD, W5329XS, W5381XA, W5381XD, W5381XS, W5389XA, W5389XD, W5389XS, W540XXA, W540XXD, W540XXS, W541XXA, W541XXD, W541XXS, W548XXA, W548XXD, W548XXS, W5501XA, W5501XD, W5501XS, W5503XA, W5503XD, W5503XS, W5509XA, W5509XD, W5509XS, W5511XA, W5511XD, W5511XS, W5512XA, W5512XD, W5512XS, W5519XA, W5519XD, W5519XS, W5521XA, W5521XD, W5521XS, W5522XA, W5522XD, W5522XS, W5529XA, W5529XD, W5529XS, W5531XA, W5531XD, W5531XS, W5532XA, W5532XD, W5532XS, W5539XA, W5539XD, W5539XS, W5541XA, W5541XD, W5541XS, W5542XA, W5542XD, W5542XS, W5549XA, W5549XD, W5549XS, W5551XA, W5551XD, W5551XS, W5552XA, W5552XD, W5552XS, W5559XA, W5559XD, W5559XS, W5581XA, W5581XD, W5581XS, W5582XA, W5582XD, W5582XS,

	W5589XA, W5589XD, W5589XS, W5601XA, W5601XD, W5601XS, W5602XA, W5602XD, W5602XS, W5609XA, W5609XD, W5609XS, W5611XA, W5611XD, W5611XS, W5612XA, W5612XD, W5612XS, W5619XA, W5619XD, W5619XS, W5621XA, W5621XD, W5621XS, W5622XA, W5622XD, W5622XS, W5629XA, W5629XD, W5629XS, W5631XA, W5631XD, W5631XS, W5632XA, W5632XD, W5632XS, W5639XA, W5639XD, W5639XS, W5641XA, W5641XD, W5641XS, W5642XA, W5642XD, W5642XS, W5649XA, W5649XD, W5649XS, W5651XA, W5651XD, W5651XS, W5652XA, W5652XD, W5652XS, W5659XA, W5659XD, W5659XS, W5681XA, W5681XD, W5681XS, W5682XA, W5682XD, W5682XS, W5689XA, W5689XD, W5689XS, W57XXXA, W57XXXD, W57XXXS, W5801XA, W5801XD, W5801XS, W5802XA, W5802XD, W5802XS, W5803XA, W5803XD, W5803XS, W5809XA, W5809XD, W5809XS, W5811XA, W5811XD, W5811XS, W5812XA, W5812XD, W5812XS, W5813XA, W5813XD, W5813XS, W5819XA, W5819XD, W5819XS, W5901XA, W5901XD, W5901XS, W5902XA, W5902XD, W5902XS, W5909XA, W5909XD, W5909XS, W5911XA, W5911XD, W5911XS, W5912XA, W5912XD, W5912XS, W5913XA, W5913XD, W5913XS, W5919XA, W5919XD, W5919XS, W5921XA, W5921XD, W5921XS, W5922XA, W5922XD, W5922XS, W5929XA, W5929XD, W5929XS, W5981XA, W5981XD, W5981XS, W5982XA, W5982XD, W5982XS, W5983XA, W5983XD, W5983XS, W5989XA, W5989XD, W5989XS, W60XXXA, W60XXXD, W60XXXS, W6101XA, W6101XD, W6101XS, W6102XA, W6102XD, W6102XS, W6109XA, W6109XD, W6109XS, W6111XA, W6111XD, W6111XS, W6112XA, W6112XD, W6112XS, W6119XA, W6119XD, W6119XS, W6121XA, W6121XD, W6121XS, W6122XA, W6122XD, W6122XS, W6129XA, W6129XD, W6129XS, W6132XA, W6132XD, W6132XS, W6133XA, W6133XD, W6133XS, W6139XA, W6139XD, W6139XS, W6142XA, W6142XD, W6142XS, W6143XA, W6143XD, W6143XS, W6149XA, W6149XD, W6149XS, W6151XA, W6151XD, W6151XS, W6152XA, W6152XD, W6152XS, W6159XA, W6159XD, W6159XS, W6161XA, W6161XD, W6161XS, W6162XA, W6162XD, W6162XS, W6169XA, W6169XD, W6169XS, W6191XA, W6191XD, W6191XS, W6192XA, W6192XD, W6192XS, W6199XA, W6199XD, W6199XS, W620XXA, W620XXD, W620XXS, W621XXA, W621XXD, W621XXS, W629XXA, W629XXD, W629XXS, W64XXXA, W64XXXD, W64XXXS, W65XXXA, W65XXXD, W65XXXS, W67XXXA, W67XXXD, W67XXXS, W69XXXA, W69XXXD, W69XXXS, W73XXXA, W73XXXD, W73XXXS, W74XXXA, W74XXXD, W74XXXS, W85XXXA, W85XXXD, W85XXXS, W860XXA, W860XXD, W860XXS, W861XXA, W861XXD, W861XXS, W868XXA, W868XXD, W868XXS, W880XXA, W880XXD, W880XXS, W881XXA, W881XXD, W881XXS, W888XXA, W888XXD, W888XXS, W890XXA, W890XXD, W890XXS, W891XXA, W891XXD, W891XXS,

W898XXA, W898XXD, W898XXS, W899XXA, W899XXD, W899XXS, W900XXA, W900XXD, W900XXS, W901XXA, W901XXD, W901XXS, W902XXA, W902XXD, W902XXS, W908XXA, W908XXD, W908XXS, W92XXXA, W92XXXD, W92XXXS, W9301XA, W9301XD, W9301XS, W9302XA, W9302XD, W9302XS, W9311XA, W9311XD, W9311XS, W9312XA, W9312XD, W9312XS, W932XXA, W932XXD, W932XXS, W938XXA, W938XXD, W938XXS, W940XXA, W940XXD, W940XXS, W9411XA, W9411XD, W9411XS, W9412XA, W9412XD, W9412XS, W9421XA, W9421XD, W9421XS, W9422XA, W9422XD, W9422XS, W9423XA, W9423XD, W9423XS, W9429XA, W9429XD, W9429XS, W9431XA, W9431XD, W9431XS, W9432XA, W9432XD, W9432XS, W9439XA, W9439XD, W9439XS, W99XXXA, W99XXXD, W99XXXS, X002XXA, X002XXD, X002XXS, X003XXA, X003XXD, X003XXS, X004XXA, X004XXD, X004XXS, X005XXA, X005XXD, X005XXS, X022XXA, X022XXD, X022XXS, X025XXA, X025XXD, X025XXS, X34XXXA, X34XXXD, X34XXXS, X35XXXA, X35XXXD, X35XXXS, X360XXA, X360XXD, X360XXS, X361XXA, X361XXD, X361XXS, X370XXA, X370XXD, X370XXS, X371XXA, X371XXD, X371XXS, X372XXA, X372XXD, X372XXS, X373XXA, X373XXD, X373XXS, X3741XA, X3741XD, X3741XS, X3742XA, X3742XD, X3742XS, X3743XA, X3743XD, X3743XS, X378XXA, X378XXD, X378XXS, X379XXA, X379XXD, X379XXS, X38XXXA, X38XXXD, X38XXXS, X3901XA, X3901XD, X3901XS, X3908XA, X3908XD, X3908XS, X398XXA, X398XXD, X398XXS, X500XXA, X500XXD, X500XXS, X501XXA, X501XXD, X501XXS, X503XXA, X503XXD, X503XXS, X509XXA, X509XXD, X509XXS, X52XXXA, X52XXXD, X52XXXS, X58XXXA, X58XXXD, X58XXXS, X80XXXA, X80XXXD, X80XXXS, X810XXA, X810XXD, X810XXS, X811XXA, X811XXD, X811XXS, X818XXA, X818XXD, X818XXS, X820XXA, X820XXD, X820XXS, X821XXA, X821XXD, X821XXS, X822XXA, X822XXD, X822XXS, X828XXA, X828XXD, X828XXS, X830XXA, X830XXD, X830XXS

Falls:

W000XXA, W000XXD, W000XXS, W001XXA, W001XXD, W001XXS, W002XXA, W002XXD, W002XXS, W009XXA, W009XXD, W009XXS, W010XXA, W010XXD, W010XXS, W0110XA, W0110XD, W0110XS, W01110A, W01110D, W01110S, W01111A, W01111D, W01111S, W01118A, W01118D, W01118S, W01119A, W01119D, W01119S, W01190A, W01190D, W01190S, W01198A, W01198D, W01198S, W03XXXA, W03XXXD, W03XXXS, W04XXXA, W04XXXD, W04XXXS, W050XXA, W050XXD, W050XXS, W051XXA, W051XXD, W051XXS, W052XXA, W052XXD, W052XXS, W06XXXA, W06XXXD, W06XXXS, W07XXXA, W07XXXD, W07XXXS, W08XXXA, W08XXXD, W08XXXS, W090XXA, W090XXD, W090XXS, W091XXA, W091XXD, W091XXS, W092XXA, W092XXD, W092XXS, W098XXA, W098XXD, W098XXS, W100XXA,

	W100XXD, W100XXS, W101XXA, W101XXD, W101XXS, W102XXA, W102XXD, W102XXS, W108XXA, W108XXD, W108XXS, W109XXA, W109XXD, W109XXS, W11XXXA, W11XXXD, W11XXXS, W12XXXA, W12XXXD, W12XXXS, W130XXA, W130XXD, W130XXS, W131XXA, W131XXD, W131XXS, W132XXA, W132XXD, W132XXS, W133XXA, W133XXD, W133XXS, W134XXA, W134XXD, W134XXS, W138XXA, W138XXD, W138XXS, W139XXA, W139XXD, W139XXS, W14XXXA, W14XXXD, W14XXXS, W15XXXA, W15XXXD, W15XXXS, W170XXA, W170XXD, W170XXS, W171XXA, W171XXD, W171XXS, W172XXA, W172XXD, W172XXS, W173XXA, W173XXD, W173XXS, W174XXA, W174XXD, W174XXS, W1781XA, W1781XD, W1781XS, W1782XA, W1782XD, W1782XS, W1789XA, W1789XD, W1789XS, W1800XA, W1800XD, W1800XS, W1801XA, W1801XD, W1801XS, W1802XA, W1802XD, W1802XS, W1809XA, W1809XD, W1809XS, W1811XA, W1811XD, W1811XS, W1812XA, W1812XD, W1812XS, W182XXA, W182XXD, W182XXS, W1830XA, W1830XD, W1830XS, W1831XA, W1831XD, W1831XS, W1839XA, W1839XD, W1839XS, W19XXXA, W19XXXD, W19XXXS, X013XXA, X013XXD, X013XXS, X023XXA, X023XXD, X023XXS, X033XXA, X033XXD, X033XXS<br>Multiple injuries:<br>T07, T07XXXA, T07XXXD, T07XXXS

ICD-10-CM: International Classification of Diseases, Tenth revision, Clinical Modification, T1DM: Type 1 diabetes mellitus, T2DM: Type 2 diabetes mellitus

Supplementary Table S2. Baseline characteristics of study population according to different metabolic obesity phenotypes (30-day readmission).

	Non-obese		Obese		
	Metabolically healthy (MHNO)	Metabolically unhealthy (MUNO)	Metabolically healthy (MHO)	Metabolically unhealthy (MUO)	P value*
Participants (n)	(n= 122,176)	(n=130,708)	(n = 10,798)	(n = 21,914)	—
Age (years)	78 (17)	81 (14)	71 (14)	74 (12)	<0.001
Length of stay (LOS)(days)	3 (4)	3 (4)	4 (4)	4 (4)	<0.001
Total charges (TOTCG) (dollars)	40,350 (50372)	40,296 (50719)	50,396 (61112)	47,199 (59210)	<0.001
Elective admission (n/%)					<0.001
Non-elective	96,604 (79.1)	108,925 (83.3)	7,155 (66.3)	16,094 (73.4)	
Elective	25,572 (20.9)	21,783 (16.7)	3,643 (33.7)	5,820 (26.6)	
Median household income national quartile for patient ZIP code (%)					<0.001
0 - 25%	24,648 (20.2)	28,949 (22.1)	2,535 (23.5)	5,410 (24.7)	
26 -50% (median)	31,940 (26.1)	35,068 (26.8)	2,952 (27.3)	6,241 (28.5)	
51 -75%	33,006 (27.0)	34,453 (26.4)	2,953 (27.3)	5,781 (26.4)	
76 -100%	32,582 (26.7)	32,238 (24.7)	2,358 (21.8)	4,482 (20.5)	
Smokers(%)	573 (0.5)	461 (0.4)	45 (0.4)	53 (0.2)	<0.001
Alcohol abuse(%)	1,185 (1.0)	804 (0.6)	86 (0.8)	99 (0.5)	<0.001
Lack of physical exercise	8 (0.0)	3 (0.0)	0 (0.0)	3 (0.0)	0.091
HCUP_ED[a] (value) (n/%)					<0.001
0	35,529 (29.1)	31,935 (24.4)	4,379 (40.6)	7,424 (33.9)	
1	56,530 (46.3)	60,237 (46.1)	4,111 (38.1)	8,408 (38.4)	
2	29,835 (24.4)	38,235 (29.3)	2,271 (21.0)	6,011 (27.4)	
3	2 (0.0)	0 (0.0)	1 (0.0)	0 (0.0)	
4	280 (0.2)	301 (0.2)	36 (0.3)	71 (0.3)	
Expected primary payer (%)					<0.001
Medicare	104,431	118,755	8,669	18,806	

	(85.5)	(90.9)	(80.3)	(85.8)	
Medicaid	3,359 (2.7)	2,840 (2.2)	441 (4.1)	885 (4.0)	
Private insurance	12,646 (10.4)	7,764 (5.9)	1,511 (14)	1,917 (8.7)	
Self-pay	462 (0.4)	320 (0.2)	43 (0.4)	74 (0.3)	
No charge	69 (0.1)	69 (0.1)	10 (0.1)	10 (0.0)	
Other	1,209 (1.0)	960 (0.7)	124 (1.1)	222 (1.0)	
Patient location (%)					<0.001
"Central" counties of metro areas of ≥ 1 million population	33,025 (27.0)	39,336 (30.1)	2,802 (25.9)	6,623 (30.2)	
"Fringe" counties of metro areas of ≥ 1 million population	31,484 (25.8)	33,399 (25.6)	2,748 (25.4)	5,350 (24.4)	
Counties in metro areas of 250,000 - 999,999 population	28111(23.0)	28588(21.9)	2460(22.8)	4786(21.8)	
Counties in metro areas of 50,000 - 249,999 population	12,275 (10.0)	12,482 (9.5)	1,100 (10.2)	2,191 (10.0)	
Micropolitan counties	10,010 (8.2)	9,791 (7.5)	1,011 (9.4)	1,793 (8.2)	
Not metropolitan or micropolitan counties	7,271 (6.0)	7,112 (5.4)	677 (6.3)	1,171 (5.3)	
REHABTRANSFER[b] (value) (%)					<0.001
0	119,818 (98.1)	127,785 (97.8)	10,514 (97.4)	21,255 (97.0)	
1	2,358 (1.9)	2,923 (2.2)	284 (2.6)	659 (3.0)	
Patient state					<0.001
Non-resident	5,240 (4.3)	4,445 (3.4)	570 (5.3)	812 (3.7)	
Resident	116,936 (95.7)	126,263 (96.6)	10,228 (94.7)	21,102 (96.3)	

The data are expressed as the median (interquartile range) for continuous variables, or as numberes (proportion) for Categorical variables. The superscript letter a in this table refers to HCUP indicator of emergency department record, the value 0 refers to record does not meet any HCUP Emergency Department (ED) criteria, value 1 refers to ED revenue code on record, value 2 refers to positive ED charge (when revenue center codes are not available), value 3 refers to ED CPT procedure code on record, and value 4 refers to condition P7 indication of ED admission, point of origin of ED, or admission source of ED. The superscript letter b in this table refers to a combined record involving transfer to rehabilitation, evaluation, or other aftercare, value 0 refers to not a combined record or a combined record not involving rehabilitation, evaluation, or other aftercare, and value 1 refers to combined record involving transfer to rehabilitation, evaluation, or other aftercare. *P values of Kruskal–Wallis test or χ^2 test across the four categories of metabolic obesity phenotypes are listed in the last column.

Supplementary Table S3. Baseline characteristics of study population according to different metabolic obesity phenotypes (90-day readmission).

	Non-obese		Obese		
	Metabolically healthy (MHNO)	Metabolically unhealthy (MUNO)	Metabolically healthy (MHO)	Metabolically unhealthy (MUO)	P value*
Participants (n)	(n= 102,053)	(n = 108,843)	(n = 9,084)	(n = 18,439)	—
Age (years)	78 (17)	81 (14)	71 (14)	74 (12)	<0.001
Length of stay (LOS)(days)	3(4)	4(4)	4(4)	4(4)	<0.001
Total charges (TOTCG) (dollars)	39,936 (49,951)	40,006 (50,357)	50,025 (60,146)	46,750 (58,647)	<0.001
Elective admission (n/%)					<0.001
Non-elective	80,945 (79.3)	90,932 (83.5)	6,028 (66.4)	13,621 (73.9)	
Elective	21,108 (20.7)	17,911 (16.5)	3,056 (33.6)	4,818 (26.1)	
Median household income national quartile for patient ZIP code (%)					<0.001
0 - 25%	20,576 (20.2)	24,200 (22.2)	2,149 (23.7)	4,521 (24.5)	
26 -50% (median)	26,718 (26.2)	29,217 (26.8)	2,493 (27.4)	5,238 (28.4)	
51 -75%	27,676 (27.1)	28,695 (26.4)	2,485 (27.4)	4,873 (26.4)	
76 -100%	27,083 (26.5)	26,731 (24.6)	1,957 (21.5)	3,807 (20.6)	
Smokers(%)	489 (0.5)	386 (0.4)	38 (0.4)	48 (0.3)	<0.001
Alcohol abuse(%)	1,000 (1.0)	661 (0.6)	77 (0.8)	84 (0.5)	<0.001
Lack of physical exercise	5 (0.0)	3 (0.0)	0 (0.0)	3 (0.0)	0.083
HCUP_ED[a] (value) (n/%)					<0.001
0	29,417 (28.8)	26,395 (24.3)	3,677 (40.5)	6,168 (33.5)	
1	47,294 (46.3)	50,144 (46.1)	3,469 (38.2)	7,106 (38.5)	
2	25,109 (24.6)	32,064 (29.5)	1,906 (21.0)	5,116 (27.7)	
3	1 (0.0)	0 (0.0)	1 (0.0)	0 (0.0)	
4	232 (0.2)	240 (0.2)	31 (0.3)	49 (0.3)	
Expected primary payer (%)					<0.001
Medicare	87,442	98,976 (90.9)	7,296 (80.3)	15,846	

	(85.7)			(85.9)	
Medicaid	2,816 (2.8)	2,365 (2.2)	377 (4.2)	751 (4.1)	
Private insurance	10,366 (10.2)	6,391 (5.9)	1,263 (13.9)	1,594 (8.6)	
Self-pay	388 (0.4)	271 (0.2)	35 (0.4)	58 (0.3)	
No charge	55 (0.1)	55 (0.1)	9 (0.1)	8 (0)	
Other	986 (1.0)	785 (0.7)	104 (1.1)	182 (1.0)	
Patient location (%)					<0.001
"Central" counties of metro areas of ≥ 1 million population	27,730 (27.2)	32,915 (30.2)	2,344 (25.8)	5,626 (30.5)	
"Fringe" counties of metro areas of ≥ 1 million population	26,333 (25.8)	27,778 (25.5)	2,291 (25.2)	4,535 (24.6)	
Counties in metro areas of 250,000 - 999,999 population	23,388 (22.9)	23,732 (21.8)	2,091 (23.0)	4,021 (21.8)	
Counties in metro areas of 50,000 - 249,999 population	10,204 (10.0)	10,334 (9.5)	925 (10.2)	1,822 (9.9)	
Micropolitan counties	8,333 (8.2)	8,179 (7.5)	857 (9.4)	1,468 (8.0)	
Not metropolitan or micropolitan counties	6,065 (5.9)	5905 (5.4)	576 (6.3)	967 (5.2)	
REHABTRANSFER[b] (value) (%)					<0.001
0	100,136 (98.1)	106,473 (97.8)	8,844 (97.4)	17,901 (97.1)	
1	1,917 (1.9)	2,370 (2.2)	240 (2.6)	538 (2.9)	
Patient state					<0.001
Non-resident	4,383 (4.3)	3,673 (3.4)	463 (5.1)	684 (3.7)	
Resident	97,670 (95.7)	105,170 (96.6)	8,621 (94.9)	17,755 (96.3)	

HCUP indicator of emergency department record, the value 0 refers to record does not meet any HCUP Emergency Department (ED) criteria, value 1 refers to ED revenue code on record, value 2 refers to positive ED charge (when revenue center codes are not available), value 3 refers to ED CPT procedure code on record, and value 4 refers to condition P7 indication of ED admission, point of origin of ED, or admission source of ED. The superscript letter b in this table refers to a combined record involving transfer to rehabilitation, evaluation, or other aftercare, value 0 refers to not a combined record or a combined record not involving rehabilitation, evaluation, or other aftercare, and value 1 refers to combined record involving transfer to rehabilitation, evaluation, or other aftercare. *P values of Kruskal–Wallis test or χ^2 test across the four categories of metabolic obesity phenotypes are listed in the last column.

Supplementary Table S4. Associations between metabolic obesity phenotypes with 30- and 90-day readmission.

Variable	Group	30-day readmission[1]		90-day readmission[2]	
		HR (95% CI)	*p* value	HR (95% CI)	*p* value
Metabolic obesity phenotypes	MHNO	Reference	-	Reference	-
	MUNO	1.110 (1.073-1.149)	<0.001	1.134 (1.104-1.166)	<0.001
	MHO	1.145(1.050-1.250)	0.002	1.093 (1.018-1.174)	0.014
	MUO	1.238 (1.166-1.316)	<0.001	1.263 (1.204-1.325)	<0.001

[1]The model was adjusted for age, elective admission, HCUP_ED, a combined record involving rehab transfer, primary expected payer, length of stay, patient location, patient state, median household income national quartile for patient ZIP code, smoking. [2]The model was adjusted for age, elective admission, HCUP_ED, a combined record involving rehab transfer, primary expected payer, length of stay, total charges, patient location, patient state, median household income national quartile for patient ZIP code. MHNO: metabolically healthy non-obese, MUNO: metabolically unhealthy non-obese, MHO: metabolically healthy obese, MUO: metabolically unhealthy obese.

Supplementary Table S5. Associations between single metabolic disorder phenotypes with 30-day and 90-day readmission.

Variable	Group	30-day readmission[1]		90-day readmission[2]	
		HR (95% CI)	*p* value	HR (95% CI)	*p* value
Non-obese	No metabolic disorder	Reference	-	Reference	-
	Dyslipidemia	1.093 (1.006-1.187)	0.035	1.079 (1.009-1.155)	0.027
	Hypertension	1.140 (1.072-1.212)	<0.001	1.220 (1.162-1.282)	<0.001
	Hyperglycemia	1.317 (1.141-1.520)	<0.001	1.368 (1.220-1.535)	<0.001
Obese	No metabolic disorder	1.260 (1.051-1.510)	0.012	1.183 (1.016-1.378)	0.031
	Dyslipidemia	1.038 (0.809-1.334)	0.768	1.111 (0.910-1.356)	0.301
	Hypertension	1.274 (1.131-1.435)	<0.001	1.290 (1.172-1.420)	<0.001
	Hyperglycemia	1.423 (1.035-1.955)	0.030	1.209 (0.916-1.596)	0.180

[1] The model was adjusted for age, elective admission, HCUP_ED, a combined record involving rehab transfer, primary expected payer, length of stay, patient location, patient state, median household income national quartile for patient ZIP code, smoking. [2] The model was adjusted for age, elective admission, HCUP_ED, a combined record involving rehab transfer, primary expected payer, length of stay, total charges, patient location, patient state, median household income national quartile for patient ZIP code.

Supplementary Table S6. Associations between the number of metabolic risk factors with 30-day and 90-day readmission.

Variable	Group	30-day readmission[1]		90-day readmission[2]	
		HR (95% CI)	*p* value	HR (95% CI)	*p* value
Non-obese	No metabolic risk	Reference	-	Reference	-
	1 risk	1.132 (1.068-1.199)	<0.001	1.192 (1.137-1.249)	<0.001
	2 risks	1.169 (1.104-1.238)	<0.001	1.256 (1.199-1.315)	<0.001
	3 risks	1.339 (1.255-1.429)	<0.001	1.386 (1.315-1.460)	<0.001
Obese	No metabolic risk	1.267 (1.057-1.518)	0.010	1.187 (1.019-1.383)	0.027
	1 risk	1.247 (1.121-1.387)	<0.001	1.254 (1.150-1.367)	<0.001
	2 risks	1.289 (1.179-1.409)	<0.001	1.383 (1.289-1.485)	<0.001
	3 risks	1.440 (1.311-1.583)	<0.001	1.500 (1.391-1.617)	<0.001

[1] The model was adjusted for age, elective admission, HCUP_ED, a combined record involving rehab transfer, primary expected payer, length of stay, patient location, patient state, median household income national quartile for patient ZIP code, smoking. [2] The model was adjusted for age, elective admission, HCUP_ED, a combined record involving rehab transfer, primary expected payer, length of stay, total charges, patient location, patient state, median household income national quartile for patient ZIP code.

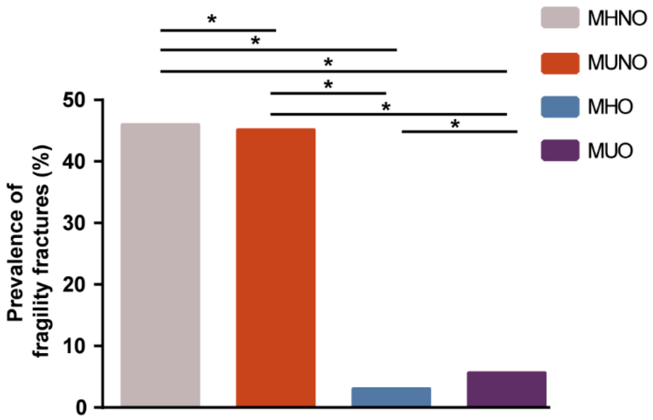

Figure S1: Prevalence of fragility fractures among different metabolic obesity phenotypes at initial admission. MHNO: metabolically healthy non-obese, MUNO: metabolically unhealthy non-obese, MHO: metabolically healthy obese, MUO: metabolically unhealthy obese.
